# Supplementary material for: What is current care for people with Long COVID in England? A qualitative interview study
Source: BMJ Open. 2024 May 16;14(5):e080967. doi: 10.1136/bmjopen-2023-080967 (PMC11107429; doi:10.1136/bmjopen-2023-080967)
Supplement: Supplementary data [file bmjopen-2023-080967supp001.pdf]

STIMULATE-ICP - WP1

Interview Schedule – Patients

Introduction

1. Review of Participant Information Sheet
2. Consent form
3. Timing and confidentiality
4. My name is
5. I am a researcher, I'm not a doctor or a psychologist and I don't have any clinical contact with your team.
6. I'm interested in finding out from you what your experiences have been with your care for Long-COVID
7. Overview of sections: your experiences, care you have received, your experiences of the service
8. There are no right answers
9. Appreciate symptoms include fatigue and brain fog, so let me know if you need to take a break etc.

**10. Precipitating events**	**Take me back, can you tell me about when you were first unwell with COVID-19 up to when you felt that you had Long Covid?**
Prompts	• Can you remember feeling unwell?<br>• Was there anything else you think may have contributed to how you were feeling? *What else going on at that time?*<br>• How do you think that experience relates to how you feel today?<br>• **Can you talk me through the support you received during this time?**<br>o E.g. family, friends, GP?<br>o How did you find out you had COVID-19? E.g. Who told you? When?<br>o How did you feel when you found out it was COVID-19?<br>o COVID-19 and other chronic conditions. Exacerbates other chronic conditions and affects management of it?<br>• When did you realise your symptoms weren't going away?<br>• **How did you find out about the post-COVID/Long Covid clinic**?
**11. Symptoms**	**Can you tell me about a time when you felt your Long-COVID was at its worst?**
Prompts	• How did it feel?<br>• How long did it last?<br>• Can you tell me how most days are for you at the moment?<br>• What's a good day for you?<br>• How long have you been feeling this way?<br>• Are there times when you don't feel this way? How long does this last?<br>• How has this impacted on your work and personal life

Patient Flow diagram

I would like to move on to think about the care you have received for your long-COVID. I'd like you to look at what some other people have experienced on their care journey at [name of clinic].

**Diagram discussion**	**When was it confirmed you had long covid? GP or after referral into clinic?**<br><br>**Has this been your experience of care?**

Sunkersing D, *et al. BMJ Open* 2024; 14:e080967. doi: 10.1136/bmjopen-2023-080967

	**Do you recognise all the parts to this care diagram?**<br>**Are any parts of your experience missing?**<br>**What part of this care pathway do you think has been the most important to your experience?**

**12. Management of Long COVID**	**What treatment are you receiving for your long-COVID at the moment?**
Prompts	• Can you tell me about the care you have received/are receiving for your Long-COVID from doctors?<br>*e.g. who (GP, clinic nurse/doctor/ physiotherapist/ occupational therapist)*<br>*e.g. where (GP clinic, hospital clinic, community/third sector/private doctor)*<br>*e.g. when (routine/booked appointments, length of appointments)*<br>*e.g. what (medications/treatment/lifestyle advice/social prescriptions/physiotherapy/ vocational rehab/ psychology)*<br>• How did you find the treatment?<br>• How helpful has this treatment been? E.g. what was most helpful and least helpful?<br>• Diagnosis/attending appointments when you're not feeling well? *Better to go and show what you're like at your worst or not be able to make it at all.*
**13. Service evaluation**	• **Can you talk me through anything you would change about the long-COVID care you have received?**
Prompts	• How long did you have to wait to be seen by your GP/ by the PCAS service/ by onward rehabilitation services?<br>• Have any parts of your care been particularly helpful or unhelpful?<br>• Have you received the care you expected to when you were referred?<br>• Did you feel listened to and understood by your GP/ clinic doctors/ any therapist you have met<br>• What would you like to happen in the future?<br>• Were there any specific types of doctor you would have liked to have seen?<br>• Would you have like to have seen a psychologist<br>• Did you receive support form a physiotherapist or occupational therapist?<br>• Would you like to go back to the clinic in the future?<br>• Would you recommend the PCAS clinic / rehabilitation service to other patients<br>• **Where do you go to for information about long covid?**<br>• Do you feel like HCPs are knowledgeable / open to new evidence about LC?

Wrapping up

14. Is there anything else you would like to tell me?
15. How have you found this interview?
16. Do you have any questions?

17. Thank you for your time.
18. Give sources of further support handout.
19. Give baseline questionnaire.

STOP RECORDING

Baseline questions to complete at end of interview.

If they have already answered this during the interview, can move on to the next question, or confirm.

Script:

Thank you for participating in this interview. We need to collect some background information about you, so that we can describe which groups of people participated in our study. This will be stored separately from the transcript of this conversation and will not include identifying information, such as your name or date of birth. If there is any question you would prefer not to answer we can move on.

- What is your age?
- What is your gender?
- How would you describe your ethnic group?
- What languages do you speak?
- What is your highest level of educational qualification?
- What is the nature of your employment? (If you're no longer employed due to LC symptoms, what was your previous job)
- Aside from your Long Covid symptoms, do you have any longstanding illnesses, diseases or medical conditions? These can include conditions relating to your physical and mental health (e.g. asthma, depression, diabetes, previous heart attack, osteoporosis).

Information for interviewers

Do not recommend that patients should complain. However if they ask where to complain or raise concerns, you can direct them to their local PALS. Information for that is in the participant information sheet, or can offer to resend the details.

If patient is very distressed, you can recommend that they get in touch with their GP or post-covid service. Similarly, you can remind them that they can let the GP know if anything has changed. You can let them know that the post-covid service has offered that we can help them connect with them, if they would like to see them.

If you have an immediate concern that there is a risk of harm to the patient or to others, e.g. they express suicidal thoughts without prompting, you have a duty to report this. In this instance, you should phone the site clinical lead. You can inform the patient that it is your responsibility to report this, and go ahead without their consent. However you should only do this if you have a compelling reason to do so; otherwise do not breach confidentiality, suggest that the patient follows up with the GP or post-covid service.

Sunkersing D, *et al. BMJ Open* 2024; 14:e080967. doi: 10.1136/bmjopen-2023-080967
