# Supplementary material for: What is current care for people with Long COVID in England? A qualitative interview study
Source: BMJ Open. 2024 May 16;14(5):e080967. doi: 10.1136/bmjopen-2023-080967 (PMC11107429; doi:10.1136/bmjopen-2023-080967)
Supplement: Supplementary data [file bmjopen-2023-080967supp002.pdf]

Workforce experience –Interview Guide

Interview guide – Interview with HCP

n=24, up to four health professional categories will be nominated at each site, and recruitment will take place within those professionals. Max four interviews per site across the six sites.

The questions below are a guide but will be adapted based on HCP type and service delivery model.

**1. Introduction**	• Introductions<br>• Purpose of discussion is to understand experience of HCPs within each site to enable us to understand how usual care is being delivered.
**2. Individual background**	**Can you tell me about your clinical background?**
Prompts	• Background, speciality, grade, years since qualification
**3. Employment in current service**	**How did you come to work in the Post COVID service?**
Prompts	• E.g. applied, seconded (where did you come from?)<br>• How long is your contract or secondment?<br>• Do you work from home or on site?<br>• Did you feel that you had previous skills or experience that was relevant to this setting?<br>• Do you want to continue working in this field in the long term?<br>• Have you experienced any long COVID symptoms yourself?
**4. Training**	**Can you tell us about the induction, training and support you have received in this role?**
	• Were you offered any training when you entered the role? [*relative to what you would expect moving into another new department, specific to LC*]<br>• Are there resources available, and have you used them?<br>• For example, have you accessed the NHS Futures site for Long COVID, attended webinars?<br>• Do you feel able to keep up to date with developments?<br>• What additional training in Long-COVID care would you like? (How would you like it to be delivered)
**5. HCP experience**	**What are the main opportunities or challenges of your role?**
Prompts	• Do you feel you have adequate time in your job plan to do this work?<br>• Managing patient disappointments (waiting list, no clear treatment)

Sunkersing D, *et al. BMJ Open* 2024; 14:e080967. doi: 10.1136/bmjopen-2023-080967

Workforce experience –Interview Guide

	• Keeping up with patient research/knowledge / responding to it / non-validated interventions and home remedies / patient safety "*This is a rapidly moving area, with patients doing a lot of their own research. What are your concerns and/or strategies for handling this?"*
**6. Patient presentation**	**What is the typical presentation of patients for the services you deliver?**
Prompts	• What symptoms do patients present with most frequently?<br>• What underlying comorbidities are typically seen?<br>• How has this changed over the course of the pandemic? (e.g. different waves, variants, vaccination, etc.)?<br>• *[pick up differences from service lead answers]*
**7. Patient experience of care**	**How would you describe a typical patient pathway in this service?**
Prompts	• **Introduce map.**<br>• What is the typical experience of referral, experience in clinic, and onward referral.<br>• What are factors that lead to a different experience?<br>• What would you describe as a good outcome or a poor outcome for a patient on the pathway? [audit, challenge]<br>• How would you describe patient access to rehab or psychology support?<br>• NHS Staff?<br>• Community vs previously hospitalised Long covid patients? Has this changed?
**8. Quality of care**	**Where do you feel the service delivers good care for patients?**
Prompts	• Have you had feedback from patients on what works well, or what they would like to improve?<br>• What do you feel are the barriers to delivering good care (if any)? E.g. high vacancy rate, high demand/low capacity?<br>• Can you identify any barriers to access to care?<br>• What are the opportunities to improve patient experience of care?<br>• How do you think patient outcomes might be improved?<br>*What do we mean? Quality vs effectiveness.*
**9. Inequalities**	**In your experience, has there been equitable uptake of the service across different groups in society e.g. those from socially disadvantaged backgrounds?**
Prompts	- Who is referred vs who turns up? How do you manage DNAs?<br>- Who is accessing the clinic?

Sunkersing D, *et al. BMJ Open* 2024; 14:e080967. doi: 10.1136/bmjopen-2023-080967

Workforce experience –Interview Guide

	- Why do you think this may be?<br>- Do you think those most at need are accessing the service?<br>- Are there particular socio-demographic groups who struggle to access the clinic?<br>- What do you think may be causing the equal/unequal uptake?<br>- Is the clinic doing anything to try and actively access those most in need?
**10.**	**What kind of immediate improvements do you want to see in the service?**
	- Funding<br>- Staffing<br>- Evidence for change might lead to overloaded capacity
11. **Wrapping up**	**Opportunity to ask questions or add in further detail**

Stop recording

Baseline questions to complete at end of interview.

If they have already answered this during the interview, can move on to the next question, or just confirm details.

Script:

Thank you for participating in this interview. We need to collect some background information about you, so that we can describe which groups of people participated in our study. This will be stored separately from the transcript of this conversation and will not include identifying information, such as your name or date of birth. If there is any question you would prefer not to answer we can move on.

- What is your age?
- What is your gender?
- How would you describe your ethnic group?
- Can you provide the following details about your role:
    - Background -
    - Speciality –
    - Grade –
    - Years since qualification
- Have you had Long Covid yourself?

Sunkersing D, *et al. BMJ Open* 2024; 14:e080967. doi: 10.1136/bmjopen-2023-080967
