# Supplementary material for: What is current care for people with Long COVID in England? A qualitative interview study
Source: BMJ Open. 2024 May 16;14(5):e080967. doi: 10.1136/bmjopen-2023-080967 (PMC11107429; doi:10.1136/bmjopen-2023-080967)
Supplement: Author's manuscript [file bmjopen-2023-080967.draft_revisions.pdf]

BMJ Open is committed to open peer review. As part of this commitment we make the peer review history of every article we publish publicly available.

When an article is published we post the peer reviewers' comments and the authors' responses online. We also post the versions of the paper that were used during peer review. These are the versions that the peer review comments apply to.

The versions of the paper that follow are the versions that were submitted during the peer review process. They are not the versions of record or the final published versions. They should not be cited or distributed as the published version of this manuscript.

BMJ Open is an open access journal and the full, final, typeset and author-corrected version of record of the manuscript is available on our site with no access controls, subscription charges or pay-per-view fees (http://bmjopen.bmj.com).

If you have any questions on BMJ Open's open peer review process please email info.bmjopen@bmj.com

BMJ Open

"Exploring Current UK Care for Long COVID: Perspectives From People Living With Long COVID and Healthcare Professionals in Dedicated Post-COVID Services".

Journal:	*BMJ Open*
Manuscript ID	bmjopen-2023-080967
Article Type:	Original research
Date Submitted by the Author:	15-Oct-2023
Complete List of Authors:	Sunkersing, David; University College London, Institute of Health Informatics<br>Ramasawmy, Mel ; University College London, Institute of Health Informatics<br>Alwan, Nisreen; University of Southampton, Academic unit of Primary Care and Population Sciences, Faculty of Medicine; University of Southampton and University Hospital Southampton NHS Foundation Trust, NIHR Southampton Biomedical Research Centre<br>Clutterbuck, Donna ; University of Southampton, Academic unit of Primary Care and Population Sciences, Faculty of Medicine<br>Mu, Yi ; University College London, Institute of Health Informatics<br>Horstmanshof, Kim; University College London<br>Banerjee, Amitava; University College London, Institute of Health Informatics<br>Heightman, Melissa; University College London Hospitals NHS Foundation Trust
Keywords:	COVID-19, QUALITATIVE RESEARCH, PUBLIC HEALTH, Post-Acute COVID-19 Syndrome

SCHOLARONE™
Manuscripts

I, the Submitting Author has the right to grant and does grant on behalf of all authors of the Work (as defined in the below author licence), an exclusive licence and/or a non-exclusive licence for contributions from authors who are: i) UK Crown employees; ii) where BMJ has agreed a CC-BY licence shall apply, and/or iii) in accordance with the terms applicable for US Federal Government officers or employees acting as part of their official duties; on a worldwide, perpetual, irrevocable, royalty-free basis to BMJ Publishing Group Ltd ("BMJ") its licensees and where the relevant Journal is co-owned by BMJ to the co-owners of the Journal, to publish the Work in this journal and any other BMJ products and to exploit all rights, as set out in our licence.

The Submitting Author accepts and understands that any supply made under these terms is made by BMJ to the Submitting Author unless you are acting as an employee on behalf of your employer or a postgraduate student of an affiliated institution which is paying any applicable article publishing charge ("APC") for Open Access articles. Where the Submitting Author wishes to make the Work available on an Open Access basis (and intends to pay the relevant APC), the terms of reuse of such Open Access shall be governed by a Creative Commons licence – details of these licences and which Creative Commons licence will apply to this Work are set out in our licence referred to above.

Other than as permitted in any relevant BMJ Author's Self Archiving Policies, I confirm this Work has not been accepted for publication elsewhere, is not being considered for publication elsewhere and does not duplicate material already published. I confirm all authors consent to publication of this Work and authorise the granting of this licence.

For peer review only

Title: "Exploring Current UK Care for Long COVID: Perspectives From People Living With Long COVID and Healthcare Professionals in Dedicated Post-COVID Services".

Abstract

Introduction: Long COVID is characterised by persistent symptoms and functional disability lasting beyond four weeks after an acute COVID-19 infection. It presents a significant challenge to global populations and healthcare systems. Evaluating current Long-COVID care where dedicated services have been developed could improve management, outcomes and health service planning.

Objective: To conduct a qualitative analysis of current care for Long COVID in England.

Design: In-depth, semi-structured interviews with people living with Long COVID and Long COVID healthcare professionals.

Setting: NHS Post-COVID services in England, from November 2022 to July 2023.

Participants: 15 healthcare professionals from six NHS England (NHSE) commissioned post-COVID services and 21 people living with Long COVID currently attending or discharged (18 female; 3 male).

Results: Our study illuminated the multifaceted nature of Long COVID, emphasising its varied symptoms, their impact on peoples' lives and the complexity involved in managing this condition. We also highlight that effective communication and robust support systems are essential for an individual with Long COVID's recovery and well-being. Lastly, though there was acknowledgement that healthcare providers initially faced challenges, growing expertise in diagnosing and treating Long COVID has led to more refined patient care approaches.

Conclusion: We highlight the diverse and interconnected aspects that constitute current care for people living with Long COVID in England. This study provides valuable insights for healthcare providers, policymakers and researchers, offering a roadmap for targeted interventions and support mechanisms to enhance the quality of care and long-term outcomes of people living with Long COVID. Future research should explore the effectiveness of individual components of Long COVID care to further refine and optimise care delivery for this population.

Data availability statement

Data are available on reasonable request. Anonymised data can be made available on request.

STRENGTHS AND LIMITATIONS OF THIS STUDY

- To our knowledge, this is the first qualitative study to explore current UK care for Long COVID using perspectives from both people with Long COVID and healthcare professionals.
- This study used in-depth, semi-structured interviews to provide a rich account of current care, from participants across 6 NHSE commissioned post-COVID services.
- A limitation of the study is the absence of perspectives of Long COVID care from extreme age groups (i.e., very young and very old).

For peer review only

Introduction

In the wake of the COVID-19 pandemic, a new and unknown medical phenomenon emerged that has challenged healthcare systems and clinicians worldwide. While much of the research focus during the early stages of the pandemic was directed towards acute COVID-19 cases [1–3], a growing number of individuals began reporting persistent and debilitating symptoms long after their initial infection, a condition now recognised as "Long COVID" or "Post-Acute Sequelae of SARS-CoV-2 infection" (PASC) [4,5]. Long COVID represents a complex and poorly understood set of symptoms that can persist for weeks, months, or even longer, substantially impacting the quality of life of those affected [4,6].

As the recognition of Long COVID increased, so did the realisation of the severity of symptoms experienced by a significant proportion of individuals [7]. People living with Long COVID have reported a wide array of persistent and recurrent symptoms, including but not limited to: extreme fatigue, dyspnoea, chest pain, cognitive impairments (colloquially referred to as "brain fog"), sleep disturbances, gastrointestinal issues, joint and muscle pain and mental health challenges such as anxiety and depression [4,8,9]. These symptoms often fluctuate and manifest in a heterogeneous manner, making diagnosis and treatment particularly challenging for healthcare providers [4,8,10]. For some people, Long COVID can result in a prolonged recovery, severely impacting their ability to work, perform daily activities and maintain social relationships [11].

Given the complexities and variations in Long COVID symptoms, the development of effective management approaches remains a pressing concern. Currently, there is limited evidence on the most appropriate care pathways for people living with Long COVID and the treatment options available often rely on extrapolating from other medical conditions or providing supportive care to alleviate specific symptoms. In the UK, guidance regarding managing the effects of COVID-19 was released by NICE and funding for dedicated post-COVID services was released in October 2020[12]. Commissioning guidance for these services was developed in October 2020 and updated in July 2022[13]. Post-COVID services were set up to treat the symptoms of Long COVID and are required to deliver a holistic assessment of individuals with a multidisciplinary team, medical support and access to diagnostics and onward referral and access to rehabilitation where needed[13].

To effectively manage and improve the outcomes of people living with Long COVID, it is crucial to evaluate current healthcare approaches for this specific patient group and their health professionals.

This study aims to explore current care through structured interviews with staff and patients in a sample of 6 of the newly formed UK Post-COVID services.

Methods

People living with Long COVID, either discharged or under follow up from 6 sites (London, Derbyshire, Liverpool, Exeter, Leicester, Hull) were invited to participate virtually over Microsoft Teams in either semi-structured interviews or focus groups (from November 2022 to July 2023). Healthcare professionals providing Long COVID care at each of the 6 sites were also invited to participate. In addition to the above, the study inclusion criteria were >18 years old, English speaker and competent to consent.

For peer review only

The service manager and clinical lead for each post-COVID service, which were identified as part of a previous study [14], were asked to review their database of people living with Long COVID, including those that may have signed up to take part in research. Depending on the most appropriate site-specific means to approach people living with Long COVID we contacted (via email, via an online Long COVID group at one site (led by the post-COVID service) and/or via an internal hospital communication app at another site (MyCare application)) them to advertise the project and request their involvement. Each service manager and clinical lead additionally identified Long COVID healthcare professionals who were willing to participate in the study. We chose this approach as we considered it the most pragmatic and efficient method for collecting data.

Each participant was asked to describe their experience of care, including referral, assessment, diagnosis and treatment (see supplementary material for full interview guides). For people living with Long COVID, the aim of the semi-structured interviews/focus groups was to gain a greater understanding of their perception of care including any barriers to accessing care, their experience of the care pathway and any outcomes they experienced due to treatment. For healthcare professionals providing care for people living with Long COVID, we wanted to understand the delivery of Long COVID care from their perspective, including any challenges, historical or current. Semi-structured interviews/focus groups were selected as the data collection approach because they can provide comprehensive, profound, and detailed narratives of participants' experiences, viewpoints, and thoughts. The interviews were conducted by DS and MR via Microsoft Teams, who have both been trained in qualitative research methods and qualitative interview techniques. Interviews were conducted with both people with Long COVID and healthcare professionals until saturation was achieved and no new information was forthcoming.

Data Analysis

All semi-structured interviews were (with participant consent) audio recorded and automatically transcribed verbatim in Microsoft Teams using the 'transcribe feature', which converts speech to a text transcript with each speaker individually separated. Each transcript was reviewed for accuracy, coded and thematically analysed by DS and MR using support from the qualitative analysis software programs ATLAS.ti (version 5.8.0)[15] and NVivo V.12[16]. All participants in this study opted to take part in a semi-structured interview – i.e., no focus groups – between November 2022 to July 2023.

An inductive, data-driven approach was adopted to code the data into descriptive terms, which were collated to generate three overarching themes ((1)Understanding Long COVID: Lived Realities, (2) Long COVID Care: Communication, Support and (3) Challenges and Long COVID Pathways: Inefficiencies and Expertise) and ten, supporting, subsidiary subthemes.

Two researchers (DS and MR) met regularly to discuss the transcripts and discuss whether the overarching themes were reflective of the presented data. These findings were also shared with AB (and patient representatives) for review. After review, there was agreement that the themes reflected the transcripts appropriately.

Patient and Public Involvement (PPI)

A PPI group (n=11) features in our regular management and oversight meetings. PPI members have contributed to the research components, including informing the semi-structured

For peer review only - http://bmjopen.bmj.com/site/about/guidelines.xhtml

interview design and approach to interviews. We also discussed the findings of this study with our PPI to facilitate our interpretations of the data. The PPI group will continue to be involved in the dissemination of findings.

Results

Characteristics of people living with Long COVID are detailed in Table 1; Characteristics of healthcare professionals providing Long COVID care are detailed in Table 2.

For peer review only

Table 1: Characteristics Of People Living With Long COVID.

Characteristic		Frequency	Percentage (%)
**Sex**	Male	3	14
	Female	18	86
**Age**	18-27	0	0
	28-37	1	5
	38-47	12	57
	48-57	6	29
	58-67	0	0
	68-77	2	10
**Ethnic Group**	White-British	12	57
	White - Any other White background	3	14
	Asian or Asian British - Indian	3	14
	Other Ethnic Groups - Any other ethnic group	2	10
	Not stated	1	5
**Languages Spoken**	English (Only)	14	67
	Multiple (Including English)	7	33
**Highest Educational Qualification**	GCSE/Equivalent	1	5
	A-Level	1	5
	Apprenticeship	1	5
	Undergraduate Degree	8	38
	Master's Degree	8	38
	PhD	1	5
	Not Stated	1	5
**Profession (Current or Prior to Covid/Long COVID (LC))**	Creative arts or design	2	10
	Education	1	5
	Engineering	1	5
	Financial	2	10
	Healthcare (Clinical Facing Role)	9	43
	Marketing, Advertising or PR	1	5

For peer review only

	Public Services or Administration	2	10
	Unemployed	1	5
	Retired	1	5
	Not Stated	1	5
**Comorbidities**	Yes	16	76
	No	4	19
	Not Stated	1	5
**LC Onset**	January - April 2020	6	29
	May - August 2020	2	10
	September - December 2020	2	10
	January - April 2021	1	5
	May - August 2021	4	19
	September - December 2021	0	0
	January - April 2022	5	24
	May - August 2022	1	5
	September - December 2022	0	0
**Post-COVID Service Attendance**	January - April 2020	0	0
	May - August 2020	2	10
	September - December 2020	3	14
	January - April 2021	3	14
	May - August 2021	0	0
	September - December 2021	2	10
	January - April 2022	1	5
	May - August 2022	4	19
	September - December 2022	5	24
	January - April 2023	1	5

For peer review only

Table 2: Characteristics of Healthcare Professionals Providing Long COVID Care.

Characteristic		Frequency	Percentage (%)
**Sex**	Male	3	20
	Female	12	80
**Age**	18-27	1	7
	28-37	5	33
	38-47	3	20
	48-57	2	13
	58-67	0	0
	68-77	0	0
	Not stated	4	27
**Ethnic Group**	White-British	6	40
	White-Any other White background	2	13
	Asian or Asian British - Indian	2	13
	Other Ethnic Groups - Any other ethnic group	2	13
	Not stated	3	20
**Profession**	Clinical Psychologist	1	7
	Clinical Trials Manager	1	7
	Doctor (Gastro)	1	7
	Exercise Physiologist	2	13
	Lead Therapist (Physio)	1	7
	Nurse	1	7
	Nurse (Service Lead)	1	7
	Occupational Health Physician & Post COVID Service (Medical Lead)	1	7

For peer review only

	Physiotherapist	1	7
	Post Covid Lead	1	7
	Rehabilitation Medicine Consultant	1	7
	Respiratory Physiotherapist	1	7
	Senior Specialist Physiotherapist	1	7
	Team Lead Post Covid Service	1	7
**Have they had Long COVID?**	Yes	3	20
	No	8	53
	Not Stated	4	27

To maintain participant anonymity, it is not possible to give further details on their roles.

For peer review only

Three overarching themes were identified and are elaborated further with example supporting quotations in boxes 1 – 3.

Theme 1: "Understanding Long COVID: Lived Realities"

Within this theme, people living with Long COVID reported on several factors relating to their Long COVID experiences. The 'physical' subtheme underscores the persistent battles with fluctuating symptoms, highlighting the complexities that impact daily life (quotes 1 – 2, box 1). The 'mental health' subtheme captures the emotional toll of uncertainty, anxiety and stress related to the nature of the condition (quotes 3 – 4, box 1). The 'social' subtheme explores 'loss of identity, or self', as people living with Long COVID grapple with both understanding and support (quote 5, box 1). Lastly, the theme pays heed to the often-overlooked environmental factors, examining how the interplay between personal surroundings and the broader context can shape the Long COVID journey (quotes 6 – 7, box 1). The interviews additionally established an evident lack of universal appreciation and/or adjustment from colleagues in their work environment of the impact of Long COVID (quote 8, box 1).

Overall, these subthemes weave a narrative that underscores the complex challenges faced by people living with Long COVID.

Box 1

Subtheme	Example Supporting Quote
Physical	1. *"I've got symptoms of that I've never experienced in a life before, you know, the aching joints, the headaches." [Participant T, Male, 74]*
Physical	2. *"So I say like the first few days, it was a lot of kind of like coughing, breathlessness, fevers, et cetera. And then kind of over the first few days starting to get kind of extreme tiredness and fatigue and a lot of kind of like neurological symptoms, like headaches, didn't like light, didn't like noise, went off like food and stuff." [Participant S, Female, 30]*
Mental Health	3. *"So there's a lot going on from a kind of stress, emotional sort of perspective." [Participant J, Female, 42]*
Mental Health	4. *"I get more forgetful and disorientated loss of concentration. Memory issues. sleep, disturbance, dizziness."[Participant P, Male, 74]*
Social	5. *"You know I'm shadow of the person I was and it's very hard to to accept that…" [Participant C, Female, 41].*
Environmental	6. *"So instead of going into a cafe, for example, we'll get a coffee and sit in the park because there's less risk. So I'm still trying to maintain a life, but it's completely different. And any events like weddings, funerals or just really high risk." [Participant N, Female, 42]*
Environmental	7. *"It's really tricky and then you're working online and actually working online, I think is one of the really toughest things if you've got Long COVID." [Participant A, Female, 57]*
Working Environment	8. *"You know they don't even give me time off for my appointments. I have to, sometimes I have to do them at work if I like. Take phone calls at work. You know it's really difficult." [Participant E, Female, 46].*
Multi-dimensional	9. *"Most of the patients come with cluster of symptoms, majority fatigue, breathlessness and also muscle aches, joint pains and the brain fog is also very common. The same cognitive issues forgetfulness and some with cardiac." [Nurse (Service Lead)].*

For peer review only

Multi-dimensional	10. *"and physical symptoms with long COVID patients and they are often burdened with quite a lot of mental health issues just because of the nature of the illness." [Doctor (Gastro)].*

Healthcare professionals, in their interviews, echoed and acknowledged the multifaceted nature of Long COVID, aligning with the reports and experiences shared by people living with Long COVID in this study (quotes 9 – 10, box 1).

By addressing physical, mental, social and environmental dimensions, this theme highlights the holistic nature of care needed for people living with Long COVID navigating the complexities of the condition and underscores the importance of a comprehensive and integrated approach to their well-being.

Theme 2: "Long COVID Care: Communication, Support and Challenges"

This theme reflects the intricate journey that people living with Long COVID undertake within the healthcare system. It highlights the significance of effective communication between people living with Long COVID and healthcare professionals (quote 1, box 2), as well as the potential barriers that can arise from perceived lack of support (quote 2, box 2). The theme also acknowledges the interplay between positive clinical experiences (quote 3, box 2), positive support networks (quotes 4 – 5, box 2) and challenges that people living with Long COVID encounter throughout their journey (quotes 6 – 7, box 2), which may also include a lack of clinician knowledge of the condition. By examining these aspects, the theme sheds light on the complexities and 'patient-provider' dynamics of Long COVID care, emphasising the need for improved 'patient-provider' communication, support and more efficient pathways within the healthcare system.

Box 2

Subtheme	Example Supporting Quote
Communication	1. *"I went to my GP because I was like, I'm not getting better and I was actually really worried and I was a typical nurse, I was like, oh God, have I got something else going on brain wise. And I was very much dismissed by the GP – she was quite unkind, actually. I think she just thought I was a bit nuts. I think she just thought its kind of PTSD, gone a bit nuts and very dismissive…". [Participant J, Female, 42]*
Perceived Lack of Support	2. [Referring to their GP Practice] *"It's impossible. You can't contact them in any way by phone or on the portal, and so you send them emails, and then no one, no one replies. They just ignore you". [Participant E, Female 46]*
Positive Engagement	3. *"And they've [referring to clinicians involved in their care] been more than amazing like I can't even tell you. They they've they've listened to me. They've cared for me when they were really worried. I had some problems with work and sick leave, and whatever my GP was like, I'm gonna call you in two weeks' time just to see how you are, you know, unnecessarily, really, but just kindness. And yeah, they've been amazing. I can't praise them highly enough.". [Participant E, Female, 46]*
Support Networks	4. *"So the Facebook community. I'm not a social media user really, but that community was was amazing because you knew for the first time that*

For peer review only

	it wasn't just you and it wasn't. You weren't alone. So that was incredible.", [Participant N, Female, 42]
Support Networks	*5. "Like, as I say, there's sort of support from friends also with Long COVID or you know, and obviously friends and family members.". [Participant G, Female, 44]*
Challenges	*6. "And this condition, it's not stable on the level bad or or or good. It's very one way one side or the other, it can be very nice and next day it can be very bad and and tomorrow I'm again good day and it's like fluctuate all the time. All the time." . [Participant F, Female, 55]*
Challenges	*7. "The respiratory physios from [Hospital name removed] were, you know, still being very nice and still being very supportive but basically saying, 'look, we we feel we've got to the end of what we can do. We're not Long COVID specialists, we're not funded for for doing Long COVID we think that any residual problems in your breathing patterns are all mixed up with autonomic dysfunction. We can't, we can't just make the breathing better without making everything else better'.". [Participant G, Female, 44]*
Challenges	*8. "They didn't have the capacity to take on any of this work. So we we wanted to have very much a multispecialty approach to managing long COVID. But our respiratory physicians and cardiologists didn't have the time available to to join the team. So the challenges have been that from a medical perspective, it's very much fallen solely on me." [Rehabilitation Medicine Consultant].*
Challenges	*9. "You know, working with people with long COVID can feel quite daunting unless you've seen a lot of it. And the confidence that comes with kind of managing that clinical uncertainty of what's in front of you only comes through with experience." [Physiotherapist].*
Challenges	*10. "So I think sort of an a key barrier is that we've that we are overcoming and have to a certain extent, but remains one that we still have to navigate and that is how we work across the integrated care system. So we're all on different electronic records and the information governance agreements extend to some things and not others. We don't yet have sight of how our patients are doing. You know, we assess them in clinic and we'll send them to rehab, but we don't really get hearing about their outcomes and that that is that is problematic both in terms of how we assess whether what we're doing is effective and and how we coordinate care, but it also limits us in our delivery." [Physiotherapist].*

This theme particularly underscores the importance of fostering an environment where people living with Long COVID can a) contact their healthcare provider and b) openly express their experiences without fear of disbelief. Moreover, the significance of a robust support network cannot be overstated in the context of Long COVID.

The interviews conducted with healthcare professionals served to validate many of the challenges reported by patients, shedding light on the complexity of the issues at hand. While corroborating some patient experiences, healthcare professionals also emphasised that these challenges were the result of various contributing factors. One notable concern raised was the lack of capacity and available time within healthcare systems (quote 8, box 2), which often led to suboptimal patient experiences. Furthermore, the interviews highlighted the clinical

For peer review only

uncertainty surrounding the best management practices for Long COVID (quote 9, box 2), indicating a pressing need for further research and guidance (e.g., by having models of care commissioned to achieve this) in this emerging field. Lastly, healthcare professionals also underscored the difficulties of navigating integrated care systems (quote 10, box 2), highlighting the need for improved collaboration and coordination among various healthcare entities to better address the multifaceted needs of patients grappling with Long COVID.

Theme 3: "Long COVID Pathways: Inefficiencies and Expertise"

Navigating the complexities of Long COVID care often involves a series of encounters with the healthcare system, from seeking initial advice at the GP's office to engaging with specialised post-COVID services. This theme encompasses a participant's experiences, challenges, and interactions within the healthcare system, highlighting their encounters with medical consultations, seeking support, and their experiences in dedicated post-COVID services.

Box 3

Subtheme	Example Supporting Quote
Avoidance	1. *"You know, you get into the into the, into the the GP's office and you know they're typing away and you know they're they're sort of trying to make a record of what it is you're you're saying and then you get 5 minutes. That's all really 5, possibly less than 10, less than 10 anyway. A lot less than 10. And they're under pressure. And yeah, it's it's not good." [Participant T, Male, 74]*
Avoidance	2. *"Everything changed in April at our GPs. Yeah, the NHS went crazy come April, and everybody noticed. The term gas lighting came around, and we constantly. We know that they're trying to push us out, because we did cases. We spoke to our practice manager – they didn't even process my husband's kidney scans. They didn't even tell him that he needed it from his CT – that there's something wrong with my husband's kidneys. So you know, we've been treated so so badly. But we can't move GPs again, because do you know what, they're all just as bad as each other, and moving won't help us. We caught our second Covid from the local GPs, not the one we're at now, and I put reported them to care Quality commission, and they were investigated. So it took a lot of strength to do that when I was feeling unwell". [Participant H, Female, 54]*
Clinical Expertise	3. *"But you know, just also, when I was under so many specialists. Like at one time was under so many people, I couldn't remember what's going on. She [the GP] had a list and she would just say, right, what's happening with this one? What's happening with that? Let's keep a track of everything. And it was really good because someone was keeping an eye out that I wasn't missing out anything or something need chasing. So yeah, really good, really good.". [Participant E, Female, 46]*
Inefficiencies	4. *"Yeah, I think that's the like having to wait to 12 weeks kind of variability on the GP's". [Participant S, Female, 30]*
Inefficiencies	5. *"To see GP it was nightmare. I don't know if it's something to do particularly with my particular surgery. Maybe they just. I don't know, become dysfunctional. I don't know. In terms of Long COVID clinic, it was in on average between three and six months.". [Participant F, Female, 55]*

For peer review only

Post-COVID service Experiences	6. *"I would, because it was the first time I felt heard. I literally thought I was going slightly loopy, and that maybe I was imagining all of these symptoms. They [Post-COVID service] made me feel heard. They got the referrals done because I've been asked – I've been saying to my GP, 'You know I can't get upstairs. I'm sleeping on the sofa' and all of these things, and she wasn't listening, well I don't know – I think she was just lazy. But it wasn't until they [Post-COVID service] got involved that I got all the tests. I got all the blood tests and the echo and I got the referral to speech and language therapy and psychology, which has been the biggest thing for me. But also, they gave me a community through their Facebook." [Participant L, Female, 54]*
Post-COVID service Experiences	7. *[About the Post-COVID service] "It's reassurance as well. And then if they find something they obviously can tell you straight away. You don't need to go and look for for something, and then it's just even acknowledgement of of somebody understand you. And and know what what is going in your body and you are not on your own and as the doctor understand you you so that's very important." [Participant F, Female, 55]*
Post-COVID service Experiences (Healthcare Professional (HCP) Perspective)	8. *"I think everybody's learning from each other, but no, not not haven't really kind of accessed like a formal training day. Like I've dipped into webinars and bits of things that I've seen that people are colleagues. I know that are doing that I'm kind of interested in." [Physiotherapist]*
Post-COVID service Experiences (HCP Perspective)	9. *"The psychology offer could maybe be a bit stronger. I mean, it's quite good that we even consider it and we have psychologists in, but we just haven't been able to fill the role and then issues with space and staffing. Again, it's just something that's fallen to the wayside." [Doctor (Gastro)].*
Post-COVID service Experiences (HCP Perspective)	10. *"Diagnostics wise, I think at the beginning we did a lot of tests because we really didn't know what was going on. And as we've grown our clinical confidence and expertise and the number of tests reduced and but we've become interested in." [Post COVID Lead].*

Some people living with Long COVID report encounters where GPs seem to avoid delving into the intricacies of Long COVID (quotes 1 – 2, box 3). Some emphasised a sense of being rushed or their concerns being sidelined. This avoidance can lead to feelings of frustration and further exacerbate the challenges of Long COVID management. Despite these comments, however, some people living with Long COVID were keen to make a note of the positive aspects that their GP had brought about (quotes 3, box 3).

Inefficiencies in the healthcare system also emerged as a common challenge. Some people living with Long COVID described lengthy wait times for appointments and a lack of continuity in care (quotes 4 – 5, box 3). Such inefficiencies contribute to delays in receiving appropriate care and hinder the progress of people living with Long COVID.

For peer review only

The narratives of people living with Long COVID provide valuable insights into the tangible impact of changes made to the services dedicated to their care. Many participants recount their initial struggles in accessing appropriate care, emphasising the importance and need for specialised Long COVID services. However, the establishment of dedicated clinics and services tailored to people living with Long COVID has offered a sense of validation and recognition (quotes 6 – 7, box 3). These services signify a commitment to understanding the complexities of Long COVID and acknowledging its lasting effects. People living with Long COVID appear to appreciate this understanding and Long COVID expertise.

The interviews with healthcare providers shed light on the challenges they faced while operating a Long COVID Service. Across all sites, the healthcare professionals confronted a multitude of obstacles, with a significant portion stemming from the pervasive clinical uncertainty surrounding Long COVID and the absence of standardised training programs. As a result, many healthcare providers had to rely on self-directed learning to stay updated and effective in their roles (quote 8, box 3). Nevertheless, more recently, this has been addressed with a training programme developed by NHS England, which launched in June 2023[17]. Furthermore, the persistent staffing issues within these specialised services meant healthcare teams often could not deliver the best possible care for patients dealing with the long-lasting effects of COVID-19 (quote 9, box 3). Nevertheless, for many Post-COVID services, while initially there were substantial clinical unknowns, there is now greater clinical expertise and confidence, resulting in more refined approaches in Long COVID care (quote 10, box 3).

Discussion

In our study combining lived experience and health professional experience, we found three overarching themes: 1) Understanding Long COVID: Lived Realities, (2) Long COVID Care: Communication, Support and Challenges and (3) Long COVID Pathways: Inefficiencies and Expertise.

Our first theme resonates with contemporary research that underscores the intricate and varied nature of the Long COVID journey. As the diverse range of symptoms and trajectories has been highlighted in previous studies [6], our study highlights the importance of personalised care models that account for the multifaceted dimensions of the condition. Additionally, the emphasis from people living with Long COVID on mental health aligns with other research [18], which showed the psychological toll of living with Long COVID, further stressing the need for integrated mental health support as part of comprehensive Long COVID management strategies. Moreover, the varied presentation, varied healthcare experience and varied trajectory all correlate with a lack of subtyping and understanding of the underlying mechanism in Long COVID.

This study additionally highlighted that the impact of Long COVID on people extends well beyond its physiological manifestations, significantly affecting their immediate surroundings and (if applicable), their working environments. This study's findings corroborate with existing literature[19,20], underlining the interplay between people living with Long COVID and the broader contexts in which they reside and work. The disruptions observed in social circles and routines align with the observations made in other studies [21,22], highlighting the persistent challenges people living with Long COVID face as they navigate the unpredictable nature of Long COVID symptoms. Moreover, our study echoes the experiences of those with cognitive impairments and reduced energy levels, resonating with other findings [23,24] that have

For peer review only

documented the diverse impacts of Long COVID on professional functioning and adjustments required in work environments. These insights underline the urgent need for supportive structures that acknowledge the multifaceted dimensions of Long COVID, both within the personal and professional spheres, ensuring comprehensive and responsive care.

Our second theme corroborates recent research emphasising effective communication and robust support systems in Long COVID care. Similar to the other findings [25], our study echoes concerns about fragmented information dissemination and suboptimal patient-provider communication, advocating for standardised protocols and improved communication strategies in Long COVID management. Our study found that establishment and use of Long COVID-specific online groups (both patient-led and provider-led) emerge as a noteworthy peer-support mechanism, facilitating connection and information-sharing among people living with Long COVID. These findings resonate with current literature, highlighting the value of online platforms in fostering a sense of community and understanding among Long COVID sufferers. The positive impact observed in our study aligns with the observations from other studies [25,26] where they have emphasised the therapeutic benefits of peer support networks in Long COVID management, acknowledging their role in reducing feelings of isolation and offering practical insights. The potential for these digital spaces to serve as outlets for shared experiences and coping strategies reflects a promising avenue for people living with Long COVID navigating the complexities of this condition, affirming the significance of such platforms within the broader landscape of patient-centred care. Nevertheless, while these online groups can be an avenue for peer support, given their potential for a source of conflicting information and misinformation, we would not advise them for safe medical management[27,28]. The presence of clinical uncertainty, as reported by patients and healthcare professionals in this study, aligns with previous findings [29]. This underscores the persistent challenge in understanding Long COVID, signifying the need for continued research and standardised approaches to improve patient care and outcomes in this complex condition.

The third theme aligns with discussions in the literature surrounding specialised post-COVID services. The positive perception of expert care within these clinics echoes the sentiments expressed by recent research [30] stressing the importance of multidisciplinary care teams in addressing the complex and varied symptomatology of Long COVID. Similarly, the issues of healthcare avoidance and inefficiencies, identified in our study, mirror previously raised concerns about limited awareness leading to delayed care-seeking behaviours [31]. A lack of resources, which was highlighted by healthcare professionals in this study is additionally a theme previously highlighted in another study [29]. We agree with suggestions that as the body of Long COVID evidence and diagnostic criteria evolves, there may be a need to revise referral criteria for specialist services to ensure the most efficient allocation of resources [29,32].

The experiences shared by people living with Long COVID in our study highlight a disconcerting circumstance wherein GPs and other healthcare professionals seemingly avoid engaging in thorough discussions about the complexities of Long COVID. The people living with Long COVID consistently express feelings of being hurried through appointments or having their concerns dismissed. These findings align with emerging observations in the literature [31,33,34], reflecting the challenge faced by healthcare providers in addressing the nuanced needs of people living with Long COVID within the constraints of limited appointment times. Similar issues in patient-provider interactions within the context of Long COVID care have been described in other studies globally, emphasising the pressures on

For peer review only

healthcare systems exacerbated by the pandemic [31,35,36]. The existing backlog of waiting lists, compounded by the strains of the COVID-19 pandemic, have strained healthcare resources, possibly contributing to the reported challenges in addressing Long COVID comprehensively within primary care settings. These dynamics emphasise the urgent need for tailored approaches to address Long COVID within healthcare systems, fostering better communication and understanding between people living with Long COVID and healthcare providers.

Despite this, our results indicate that post-COVID services emerged as a significant source of positive impact on people living with Long COVID, offering a unique space where they could find solace in being heard and understood. While immediate clinical improvement may not always be observed, many people living with Long COVID underscore the transformative nature of their interactions with healthcare professionals in these specialised clinics. These findings resonate with existing literature, reflecting the pivotal role that post-COVID services play in addressing the complex needs of people living with Long COVID. Our study's insights align with recommendations from previous studies [37,38] where an emphasis on the importance of multidisciplinary care teams and specialised expertise in tackling the intricate symptomatology of Long COVID was given. Our findings suggest that for many Long COVID sufferers, the post-COVID services are a place where their experiences were validated and offered tailored support, thereby filling a critical void within the broader healthcare system.

Limitations

The underrepresentation of males within the participant pool is a potential limitation, which might limit the comprehensiveness of our findings and hinder a complete understanding of gender-specific experiences. Nevertheless, this may reflect the utilisation of Long COVID services in England. Secondly, the lack of ethnic diversity among participants poses a constraint on the generalisability of our results, as experiences of Long COVID could vary across different ethnic backgrounds due to factors such as genetics, socio-cultural contexts, and healthcare disparities. We know from previous research that ethnic minorities have been disproportionately affected by COVID [39]. Furthermore, the absence of extreme age groups, specifically very young and very old individuals, might result in an incomplete depiction of Long COVID's impact across the lifespan. The experiences of these age groups could offer unique insights that have been overlooked in this study.

Conclusions

By delving into the personal narratives and experiences of people living with Long COVID, we have uncovered several examples of good practice, but also challenges within the realm of healthcare provision. These findings underscore the urgent need for a patient-centred approach that considers the unique complexities of Long COVID, encompassing its physical manifestations in addition to its impact on mental health, social and environmental aspects. Further, this study emphasised the pivotal role of effective communication, specialised expertise and comprehensive support systems. Our study advocates for the integration of these insights into the development of tailored interventions and policies that can holistically address the needs of people living with Long COVID, ultimately enhancing the quality of care and improving the lives of those with this condition.

For peer review only

Footnotes

Participant Consent for Publication

Not Applicable.

Ethics Approval

This study involves human participants and ethical approval (IRAS Project ID: 303958) was granted by the Health Research Authority (HRA) and South Central—Berkshire Research Ethics Committee (REC Reference: 22/SC/0047). All participants gave informed consent to participate in the study before taking part.

Funding

This research was funded by NIHR (COV-LT2-0043) as part of the STIMULATE-ICP study. The funders had no role in the design of the study; in the collection, analyses, or interpretation of data; in the writing of the manuscript; or in the decision to publish the results.

Acknowledgements

Our sincere thanks to all participants in this study for their time and the information they provided.

This study is part of the STIMULATE-ICP study. The STIMULATE-ICP Consortium: Amitava Banerjee, Elizabeth Murray, Hakim-Moulay Dehbi, Hugh Montgomery, Sarah Clegg, Henry Goodfellow, Mel Ramasawmy, Yi Mu, Sampath Weerakkody, Ileana Selejan, David Sunkersing, Ashkan Dashtban, Paula Lorgelly, Melissa Heightman, Toby Hillman, Emma Wall, Caroline Leigh-Watkins, Denise Forshaw, Gordon Prescott, Gail Allsopp, Mark Gabbay, Gregory Lip, Dan Cuthbertson, Dan Wootton, Nefyn Williams, Mike G Crooks, Angela Green, Christina van der Feltz-Cornelis, Jenny Sweetman, Han-I Wang, Natalie Smith, Kamlesh Khunti, Lauren O'Mahoney, Rachael Evans, William D Strain, Rachel Botell, Nisreen Alwan, Donna Clutterbuck, Marija Pantelic, Chris Robson, Mike Brady, Rajarshi Banerjee, Cat Kelly, Angela Barone, Johannes Alberts, Rob Suriano, Lyth Hishmeh, Emily Attree, Jasmine Hayer, Rita Mallinson Cookson, Rachel Hext, Andrew Williams, Rachel Williams, Mag Leahy, Antony Loveless, Clare Loveless, Kim Horstmanshof, Ewen Brennan, Amanpreet Sarna, Manuel Gomes, Andrew Clegg, Valerio Benedetto, Melissa Dalton, Fauzia Begum, Fidan Turk, Michele Pansini, Anu Chandra, Gemma Clunie, Dominic Crocombe, Shane McAuliffe, Michelle Quaye, Farhat Gilani, Yusuf Jaami, and Nikki Cleary. An up-to-date version of Consortium members can be found on https://www.stimulate-icp.org/team. STIMULATE-ICP can be contacted at: info@stimulate-icp.org.

Disclaimer

The views expressed in this publication are those of the author(s) and not necessarily those of the National Institute for Health and Social Care Research or the Department for Health and Social Care.

Competing Interests

AB has received research funding from NIHR, British Medical Association, Astra Zeneca, National Institute of Aging and European Union. AB is a Trustee of Long COVID SoS. NAA

For peer review only

has contributed in an advisory capacity to the World Health Organisation and the European Union Commission's Expert Panel on effective ways of investing in health meetings in relation to post-COVID-19 condition. MH is National Specialty Advisor for Long Covid, NHS England.

All other authors report no competing interests.

Supplementary Material

1. Semi-structured Interview Guide (Patients)
2. Semi-structured Interview Guide (Healthcare Professionals)

Word Count (Excluding Tables and References): 3887

Author Contributions: AB, MH and the STIMULATE-ICP Consortium conceived and designed the study. DS and MR conducted interviews with both people with Long COVID and healthcare professionals. DS conducted data analysis and drafted the paper. MR, NA, DC, YM, KH, AB and MH provided constructive feedback on drafts of the manuscript and approved the final version. DS is the guarantor.

References:

1. Cheng C, Zhang D, Dang D, Geng J, Zhu P, Yuan M, et al. The incubation period of COVID-19: a global meta-analysis of 53 studies and a Chinese observation study of 11 545 patients. Infect Dis Poverty. 2021 Sep 17;10(1):119.

2. Huang C, Wang Y, Li X, Ren L, Zhao J, Hu Y, et al. Clinical features of patients infected with 2019 novel coronavirus in Wuhan, China. Lancet [Internet]. 2020 Feb 15 [cited 2023 Sep 19];395(10223):497–506. Available from: https://pubmed.ncbi.nlm.nih.gov/31986264/

3. Jin X, Lian JS, Hu JH, Gao J, Zheng L, Zhang YM, et al. Epidemiological, clinical and virological characteristics of 74 cases of coronavirus-infected disease 2019 (COVID-19) with gastrointestinal symptoms. Gut [Internet]. 2020 Jun 1 [cited 2023 Sep 19];69(6):1002–9. Available from: https://gut.bmj.com/content/69/6/1002

4. Davis HE, McCorkell L, Vogel JM, Topol EJ. Long COVID: major findings, mechanisms and recommendations. Nature Reviews Microbiology 2023 21:3 [Internet]. 2023 Jan 13 [cited 2023 Sep 19];21(3):133–46. Available from: https://www.nature.com/articles/s41579-022-00846-2

5. Parker AM, Brigham E, Connolly B, McPeake J, Agranovich A V., Kenes MT, et al. Addressing the post-acute sequelae of SARS-CoV-2 infection: a multidisciplinary model of care. Lancet Respir Med [Internet]. 2021 Nov 1 [cited 2023 Sep 19];9(11):1328–41. Available from: https://pubmed.ncbi.nlm.nih.gov/34678213/

6. Michelen M, Manoharan L, Elkheir N, Cheng V, Dagens A, Hastie C, et al. Characterising long COVID: a living systematic review. BMJ Glob Health [Internet]. 2021 Sep 1 [cited 2023 Sep 19];6(9):e005427. Available from: https://gh.bmj.com/content/6/9/e005427

7. Raveendran A V., Jayadevan R, Sashidharan S. Long COVID: An overview. Diabetes Metab Syndr [Internet]. 2021 May 1 [cited 2023 Sep 19];15(3):869–75. Available from: https://pubmed.ncbi.nlm.nih.gov/33892403/

8. Koc HC, Xiao J, Liu W, Li Y, Chen G. Long COVID and its Management. Int J Biol Sci [Internet]. 2022 [cited 2023 Sep 19];18(12):4768–80. Available from: https://pubmed.ncbi.nlm.nih.gov/35874958/

For peer review only

9. Walker S, Goodfellow H, Pookarnjanamorakot P, Murray E, Bindman J, Blandford A, et al. Impact of fatigue as the primary determinant of functional limitations among patients with post-COVID-19 syndrome: a cross-sectional observational study. BMJ Open [Internet]. 2023 Jun 1 [cited 2023 Sep 19];13(6):e069217. Available from: https://bmjopen.bmj.com/content/13/6/e069217

10. Mantovani A, Morrone MC, Patrono C, Santoro MG, Schiaffino S, Remuzzi G, et al. Long Covid: where we stand and challenges ahead. Cell Death & Differentiation 2022 29:10 [Internet]. 2022 Sep 7 [cited 2023 Sep 19];29(10):1891–900. Available from: https://www.nature.com/articles/s41418-022-01052-6

11. Aghaei A, Zhang R, Taylor S, Tam CC, Yang CH, Li X, et al. Impact of COVID-19 symptoms on social aspects of life among female long haulers: A qualitative study. Res Sq. 2022 Feb 10;

12. NICE. COVID-19 rapid guideline: managing the long-term effects of COVID-19 [Internet]. 2020 [cited 2023 Oct 9]. Available from: https://www.nice.org.uk/guidance/ng188

13. NHS England. National commissioning guidance for post COVID services [Internet]. 2022 [cited 2023 Oct 9]. Available from: https://www.england.nhs.uk/publication/national-commissioning-guidance-for-post-covid-services/

14. Forshaw D, Wall EC, Prescott G, Dehbi HM, Green A, Attree E, et al. STIMULATE-ICP: A pragmatic, multi-centre, cluster randomised trial of an integrated care pathway with a nested, Phase III, open label, adaptive platform randomised drug trial in individuals with Long COVID: A structured protocol. PLoS One [Internet]. 2023 Feb 1 [cited 2023 Sep 19];18(2). Available from: https://pubmed.ncbi.nlm.nih.gov/36791116/

15. ATLAS.ti. Scientific Software Development GmbH; 2023.

16. Lumivero. NVivo (Version 12). 2017.

17. NHS England. Post COVID Services [Internet]. 2023 [cited 2023 Oct 10]. Available from: https://www.e-lfh.org.uk/programmes/post-covid-services/

18. Houben-Wilke S, Goërtz YMJ, Delbressine JM, Vaes AW, Meys R, Machado FVC, et al. The Impact of Long COVID-19 on Mental Health: Observational 6-Month Follow-Up Study. JMIR Ment Health 2022;9(2):e33704 https://mental.jmir.org/2022/2/e33704 [Internet]. 2022 Feb 24 [cited 2023 Sep 19];9(2):e33704. Available from: https://mental.jmir.org/2022/2/e33704

19. Ziauddeen N, Gurdasani D, O'Hara ME, Hastie C, Roderick P, Yao G, et al. Characteristics and impact of Long Covid: Findings from an online survey. PLoS One. 2022;17(3 March).

20. Suran M. Long COVID Linked With Unemployment in New Analysis. Vol. 329, JAMA. 2023.

21. Samper-Pardo M, Oliván-Blázquez B, Magallón-Botaya R, Méndez-López F, Bartolomé-Moreno C, León-Herrera S. The emotional well-being of Long COVID patients in relation to their symptoms, social support and stigmatization in social and health services: a qualitative study. BMC Psychiatry [Internet]. 2023 Dec 1 [cited 2023 Sep 19];23(1):1–13. Available from: https://bmcpsychiatry.biomedcentral.com/articles/10.1186/s12888-022-04497-8

22. Schmachtenberg T, Müller F, Kranz J, Dragaqina A, Wegener G, Königs G, et al. How do long COVID patients perceive their current life situation and occupational perspective? Results of a qualitative interview study in Germany. Front Public Health [Internet]. 2023 [cited 2023 Sep 19];11. Available from: /pmc/articles/PMC10034079/

For peer review only

23. De Luca R, Bonanno M, Calabrò RS. Psychological and Cognitive Effects of Long COVID: A Narrative Review Focusing on the Assessment and Rehabilitative Approach. J Clin Med [Internet]. 2022 Nov 1 [cited 2023 Sep 19];11(21). Available from: https://pubmed.ncbi.nlm.nih.gov/36362782/

24. Zahouani I, Desmeules F, Perreault K, Campeau-Lecours A, Best K, Beaulieu-Bonneau S, et al. Physical and cognitive impairments in people suffering from long COVID: protocol for a longitudinal population-based cohort study. BMJ Open [Internet]. 2023 [cited 2023 Sep 19];13:64054. Available from: http://bmjopen.bmj.com/

25. Russell D, Spence NJ, Chase JAD, Schwartz T, Tumminello CM, Bouldin E. Support amid uncertainty: Long COVID illness experiences and the role of online communities. SSM - Qualitative Research in Health. 2022 Dec 1;2:100177.

26. Day HLS. Exploring Online Peer Support Groups for Adults Experiencing Long COVID in the United Kingdom: Qualitative Interview Study. J Med Internet Res [Internet]. 2022 May 1 [cited 2023 Sep 19];24(5). Available from: /pmc/articles/PMC9128729/

27. Brown K, Yahyouche A, Haroon S, Camaradou J, Turner G. Long COVID and self-management. The Lancet. 2022 Jan;399(10322):355.

28. Aiyegbusi OL, Hughes SE, Turner G, Rivera SC, McMullan C, Chandan JS, et al. Symptoms, complications and management of long COVID: a review. J R Soc Med. 2021 Sep 15;114(9):428–42.

29. Ladds E, Rushforth A, Wieringa S, Taylor S, Rayner C, Husain L, et al. Developing services for long COVID: lessons from a study of wounded healers. Clin Med (Lond) [Internet]. 2021 Jan 1 [cited 2023 Sep 19];21(1):59–65. Available from: https://pubmed.ncbi.nlm.nih.gov/33479069/

30. Parkin A, Davison J, Tarrant R, Ross D, Halpin S, Simms A, et al. A Multidisciplinary NHS COVID-19 Service to Manage Post-COVID-19 Syndrome in the Community. J Prim Care Community Health [Internet]. 2021 [cited 2023 Sep 19];12. Available from: https://pubmed.ncbi.nlm.nih.gov/33880955/

31. Au L, Capotescu C, Eyal G, Finestone G. Long covid and medical gaslighting: Dismissal, delayed diagnosis, and deferred treatment. SSM Qualitative research in health. 2022 Dec;2:100167.

32. Devereaux A, Yang H, Seda G, Sankar V, Maves RC, Karanjia N, et al. Optimizing Scarce Resource Allocation During COVID-19: Rapid Creation of a Regional Health-Care Coalition and Triage Teams in San Diego County, California. Disaster Med Public Health Prep [Internet]. 2022 Feb 10 [cited 2023 Sep 19];16(1):321–7. Available from: https://www.cambridge.org/core/journals/disaster-medicine-and-public-health-preparedness/article/optimizing-scarce-resource-allocation-during-covid19-rapid-creation-of-a-regional-healthcare-coalition-and-triage-teams-in-san-diego-county-california/2C5495005A8CF15F4971884A2B80E578

33. Purewal S, Byers K, Jamieson K, Zolfaghari N. People with long COVID continue to experience medical gaslighting more than 3 years into the pandemic. The Conversation. 2023.

34. As a doctor with long covid, I feel abandoned by the NHS. Vol. 380, BMJ (Clinical research ed.). 2023.

35. Hussain FA. Facilitating care: a biopsychosocial perspective on long COVID. British Journal of General Practice [Internet]. 2022 Jan 1 [cited 2023 Sep 19];72(714):30–1. Available from: https://bjgp.org/content/72/714/30

For peer review only

36. Kingstone T, Taylor AK, O'Donnell CA, Atherton H, Blane DN, Chew-Graham CA. Finding the "right" GP: a qualitative study of the experiences of people with long-COVID. BJGP Open [Internet]. 2020 Dec 1 [cited 2023 Sep 19];4(5):1–12. Available from: https://bjgpopen.org/content/4/5/bjgpopen20X101143

37. Greenhalgh T, Sivan M, Delaney B, Evans R, Milne R. Long covid—an update for primary care. BMJ [Internet]. 2022 Sep 22 [cited 2023 Sep 19];378. Available from: https://www.bmj.com/content/378/bmj-2022-072117

38. Nalbandian A, Sehgal K, Gupta A, Madhavan M V., McGroder C, Stevens JS, et al. Post-acute COVID-19 syndrome. Nature Medicine 2021 27:4 [Internet]. 2021 Mar 22 [cited 2023 Sep 19];27(4):601–15. Available from: https://www.nature.com/articles/s41591-021-01283-z

39. Jaljaa A, Caminada S, Tosti ME, D'Angelo F, Angelozzi A, Isonne C, et al. Risk of SARS-CoV-2 infection in migrants and ethnic minorities compared with the general population in the European WHO region during the first year of the pandemic: a systematic review. BMC Public Health [Internet]. 2022 Dec 1 [cited 2023 Sep 19];22(1):1–14. Available from: https://bmcpublichealth.biomedcentral.com/articles/10.1186/s12889-021-12466-1

For peer review only

STIMULATE-ICP - WP1

Interview Schedule – Patients

Introduction

1. Review of Participant Information Sheet
2. Consent form
3. Timing and confidentiality
4. My name is
5. I am a researcher, I'm not a doctor or a psychologist and I don't have any clinical contact with your team.
6. I'm interested in finding out from you what your experiences have been with your care for Long-COVID
7. Overview of sections: your experiences, care you have received, your experiences of the service
8. There are no right answers
9. Appreciate symptoms include fatigue and brain fog, so let me know if you need to take a break etc.

For peer review only

**10. Precipitating events**	**Take me back, can you tell me about when you were first unwell with COVID-19 up to when you felt that you had Long Covid?**
Prompts	• Can you remember feeling unwell?<br>• Was there anything else you think may have contributed to how you were feeling? *What else going on at that time?*<br>• How do you think that experience relates to how you feel today?<br>• **Can you talk me through the support you received during this time?**<br>○ E.g. family, friends, GP?<br>○ How did you find out you had COVID-19? E.g. Who told you? When?<br>○ How did you feel when you found out it was COVID-19?<br>○ COVID-19 and other chronic conditions. Exacerbates other chronic conditions and affects management of it?<br>• When did you realise your symptoms weren't going away?<br>• **How did you find out about the post-COVID/Long Covid clinic**?
**11. Symptoms**	**Can you tell me about a time when you felt your Long-COVID was at its worst?**
Prompts	• How did it feel?<br>• How long did it last?<br>• Can you tell me how most days are for you at the moment?<br>• What's a good day for you?<br>• How long have you been feeling this way?<br>• Are there times when you don't feel this way? How long does this last?<br>• How has this impacted on your work and personal life

Patient Flow diagram

I would like to move on to think about the care you have received for your long-COVID. I'd like you to look at what some other people have experienced on their care journey at [name of clinic].

**Diagram discussion**	**When was it confirmed you had long covid? GP or after referral into clinic?**<br><br>**Has this been your experience of care?**

For peer review only - http://bmjopen.bmj.com/site/about/guidelines.xhtml

	**Do you recognise all the parts to this care diagram?**<br>**Are any parts of your experience missing?**<br>**What part of this care pathway do you think has been the most important to your experience?**

**12. Management of Long COVID**	**What treatment are you receiving for your long-COVID at the moment?**
Prompts	• Can you tell me about the care you have received/are receiving for your Long-COVID from doctors?<br>*e.g. who (GP, clinic nurse/doctor/ physiotherapist/ occupational therapist)*<br>*e.g. where (GP clinic, hospital clinic, community/third sector/private doctor)*<br>*e.g. when (routine/booked appointments, length of appointments)*<br>*e.g. what (medications/treatment/lifestyle advice/social prescriptions/physiotherapy/ vocational rehab/ psychology)*<br>• How did you find the treatment?<br>• How helpful has this treatment been? E.g. what was most helpful and least helpful?<br>• Diagnosis/attending appointments when you're not feeling well? *Better to go and show what you're like at your worst or not be able to make it at all.*
**13. Service evaluation**	• **Can you talk me through anything you would change about the long-COVID care you have received?**
Prompts	• How long did you have to wait to be seen by your GP/ by the PCAS service/ by onward rehabilitation services?<br>• Have any parts of your care been particularly helpful or unhelpful?<br>• Have you received the care you expected to when you were referred?<br>• Did you feel listened to and understood by your GP/ clinic doctors/ any therapist you have met<br>• What would you like to happen in the future?<br>• Were there any specific types of doctor you would have liked to have seen?<br>• Would you have like to have seen a psychologist<br>• Did you receive support form a physiotherapist or occupational therapist?<br>• Would you like to go back to the clinic in the future?<br>• Would you recommend the PCAS clinic / rehabilitation service to other patients<br>• **Where do you go to for information about long covid?**<br>• Do you feel like HCPs are knowledgeable / open to new evidence about LC?

Wrapping up

14. Is there anything else you would like to tell me?
15. How have you found this interview?
16. Do you have any questions?

For peer review only - http://bmjopen.bmj.com/site/about/guidelines.xhtml

17. Thank you for your time.
18. Give sources of further support handout.
19. Give baseline questionnaire.

STOP RECORDING

Baseline questions to complete at end of interview.

If they have already answered this during the interview, can move on to the next question, or confirm.

Script:

Thank you for participating in this interview. We need to collect some background information about you, so that we can describe which groups of people participated in our study. This will be stored separately from the transcript of this conversation and will not include identifying information, such as your name or date of birth. If there is any question you would prefer not to answer we can move on.

- What is your age?
- What is your gender?
- How would you describe your ethnic group?
- What languages do you speak?
- What is your highest level of educational qualification?
- What is the nature of your employment? (If you're no longer employed due to LC symptoms, what was your previous job)
- Aside from your Long Covid symptoms, do you have any longstanding illnesses, diseases or medical conditions? These can include conditions relating to your physical and mental health (e.g. asthma, depression, diabetes, previous heart attack, osteoporosis).

Information for interviewers

Do not recommend that patients should complain. However if they ask where to complain or raise concerns, you can direct them to their local PALS. Information for that is in the participant information sheet, or can offer to resend the details.

If patient is very distressed, you can recommend that they get in touch with their GP or post-covid service. Similarly, you can remind them that they can let the GP know if anything has changed. You can let them know that the post-covid service has offered that we can help them connect with them, if they would like to see them.

If you have an immediate concern that there is a risk of harm to the patient or to others, e.g. they express suicidal thoughts without prompting, you have a duty to report this. In this instance, you should phone the site clinical lead. You can inform the patient that it is your responsibility to report this, and go ahead without their consent. However you should only do this if you have a compelling reason to do so; otherwise do not breach confidentiality, suggest that the patient follows up with the GP or post-covid service.

For peer review only - http://bmjopen.bmj.com/site/about/guidelines.xhtml

Interview guide – Interview with HCP

n=24, up to four health professional categories will be nominated at each site, and recruitment will take place within those professionals. Max four interviews per site across the six sites.

The questions below are a guide but will be adapted based on HCP type and service delivery model.

**1. Introduction**	• Introductions<br>• Purpose of discussion is to understand experience of HCPs within each site to enable us to understand how usual care is being delivered.
**2. Individual background**	**Can you tell me about your clinical background?**
Prompts	• Background, speciality, grade, years since qualification
**3. Employment in current service**	**How did you come to work in the Post COVID service?**
Prompts	• E.g. applied, seconded (where did you come from?)<br>• How long is your contract or secondment?<br>• Do you work from home or on site?<br>• Did you feel that you had previous skills or experience that was relevant to this setting?<br>• Do you want to continue working in this field in the long term?<br>• Have you experienced any long COVID symptoms yourself?
**4. Training**	**Can you tell us about the induction, training and support you have received in this role?**
	• Were you offered any training when you entered the role? [*relative to what you would expect moving into another new department, specific to LC*]<br>• Are there resources available, and have you used them?<br>• For example, have you accessed the NHS Futures site for Long COVID, attended webinars?<br>• Do you feel able to keep up to date with developments?<br>• What additional training in Long-COVID care would you like? (How would you like it to be delivered)
**5. HCP experience**	**What are the main opportunities or challenges of your role?**
Prompts	• Do you feel you have adequate time in your job plan to do this work?<br>• Managing patient disappointments (waiting list, no clear treatment)

For peer review only - http://bmjopen.bmj.com/site/about/guidelines.xhtml

Workforce experience –Interview Guide

	• Keeping up with patient research/knowledge / responding to it / non-validated interventions and home remedies / patient safety "*This is a rapidly moving area, with patients doing a lot of their own research. What are your concerns and/or strategies for handling this?*"
**6. Patient presentation**	**What is the typical presentation of patients for the services you deliver?**
Prompts	• What symptoms do patients present with most frequently?<br>• What underlying comorbidities are typically seen?<br>• How has this changed over the course of the pandemic? (e.g. different waves, variants, vaccination, etc.)?<br>• *[pick up differences from service lead answers]*
**7. Patient experience of care**	**How would you describe a typical patient pathway in this service?**
Prompts	• **Introduce map.**<br>• What is the typical experience of referral, experience in clinic, and onward referral.<br>• What are factors that lead to a different experience?<br>• What would you describe as a good outcome or a poor outcome for a patient on the pathway? [audit, challenge]<br>• How would you describe patient access to rehab or psychology support?<br>• NHS Staff?<br>• Community vs previously hospitalised Long covid patients? Has this changed?
**8. Quality of care**	**Where do you feel the service delivers good care for patients?**
Prompts	• Have you had feedback from patients on what works well, or what they would like to improve?<br>• What do you feel are the barriers to delivering good care (if any)? E.g. high vacancy rate, high demand/low capacity?<br>• Can you identify any barriers to access to care?<br>• What are the opportunities to improve patient experience of care?<br>• How do you think patient outcomes might be improved?<br><br>*What do we mean? Quality vs effectiveness.*
**9. Inequalities**	**In your experience, has there been equitable uptake of the service across different groups in society e.g. those from socially disadvantaged backgrounds?**
Prompts	- Who is referred vs who turns up? How do you manage DNAs?<br>- Who is accessing the clinic?

For peer review only - http://bmjopen.bmj.com/site/about/guidelines.xhtml

Workforce experience –Interview Guide

	- Why do you think this may be?<br>- Do you think those most at need are accessing the service?<br>- Are there particular socio-demographic groups who struggle to access the clinic?<br>- What do you think may be causing the equal/unequal uptake?<br>- Is the clinic doing anything to try and actively access those most in need?
**10.**	**What kind of immediate improvements do you want to see in the service?**
	- Funding<br>- Staffing<br>- Evidence for change might lead to overloaded capacity
11. **Wrapping up**	**Opportunity to ask questions or add in further detail**

Stop recording

Baseline questions to complete at end of interview.

If they have already answered this during the interview, can move on to the next question, or just confirm details.

Script:

Thank you for participating in this interview. We need to collect some background information about you, so that we can describe which groups of people participated in our study. This will be stored separately from the transcript of this conversation and will not include identifying information, such as your name or date of birth. If there is any question you would prefer not to answer we can move on.

- What is your age?
- What is your gender?
- How would you describe your ethnic group?
- Can you provide the following details about your role:
    - Background -
    - Speciality –
    - Grade –
    - Years since qualification
- Have you had Long Covid yourself?

For peer review only - http://bmjopen.bmj.com/site/about/guidelines.xhtml

Standards for Reporting Qualitative Research (SRQR)*

http://www.equator-network.org/reporting-guidelines/srqr/

Title and abstract

	Page/line no(s).
**Title** - Concise description of the nature and topic of the study Identifying the study as qualitative or indicating the approach (e.g., ethnography, grounded theory) or data collection methods (e.g., interview, focus group) is recommended	1
**Abstract** - Summary of key elements of the study using the abstract format of the intended publication; typically includes background, purpose, methods, results, and conclusions	1

Introduction

**Problem formulation** - Description and significance of the problem/phenomenon studied; review of relevant theory and empirical work; problem statement	2
**Purpose or research question** - Purpose of the study and specific objectives or questions	2

Methods

**Qualitative approach and research paradigm** - Qualitative approach (e.g., ethnography, grounded theory, case study, phenomenology, narrative research) and guiding theory if appropriate; identifying the research paradigm (e.g., postpositivist, constructivist/ interpretivist) is also recommended; rationale**	2-3
**Researcher characteristics and reflexivity** - Researchers' characteristics that may influence the research, including personal attributes, qualifications/experience, relationship with participants, assumptions, and/or presuppositions; potential or actual interaction between researchers' characteristics and the research questions, approach, methods, results, and/or transferability	3
**Context** - Setting/site and salient contextual factors; rationale**	2
**Sampling strategy** - How and why research participants, documents, or events were selected; criteria for deciding when no further sampling was necessary (e.g., sampling saturation); rationale**	2-3
**Ethical issues pertaining to human subjects** - Documentation of approval by an appropriate ethics review board and participant consent, or explanation for lack thereof; other confidentiality and data security issues	16 (Ethics Approval)
**Data collection methods** - Types of data collected; details of data collection procedures including (as appropriate) start and stop dates of data collection and analysis, iterative process, triangulation of sources/methods, and modification of procedures in response to evolving study findings; rationale**	3 (Methods)

For peer review only

For peer review only - http://bmjopen.bmj.com/site/about/guidelines.xhtml

**Data collection instruments and technologies** - Description of instruments (e.g., interview guides, questionnaires) and devices (e.g., audio recorders) used for data collection; if/how the instrument(s) changed over the course of the study	3 (Data Analysis)
**Units of study** - Number and relevant characteristics of participants, documents, or events included in the study; level of participation (could be reported in results)	2 (Methods); 3 (Table 1); 5 (Table 2)
**Data processing** - Methods for processing data prior to and during analysis, including transcription, data entry, data management and security, verification of data integrity, data coding, and anonymization/de-identification of excerpts	2-3 (Methods, Data Analysis)
**Data analysis** - Process by which inferences, themes, etc., were identified and developed, including the researchers involved in data analysis; usually references a specific paradigm or approach; rationale**	3 (Data Analysis)
**Techniques to enhance trustworthiness** - Techniques to enhance trustworthiness and credibility of data analysis (e.g., member checking, audit trail, triangulation); rationale**	3 (Data Analysis)

Results/findings

**Synthesis and interpretation** - Main findings (e.g., interpretations, inferences, and themes); might include development of a theory or model, or integration with prior research or theory	4-13
**Links to empirical data** - Evidence (e.g., quotes, field notes, text excerpts, photographs) to substantiate analytic findings	4-13

Discussion

**Integration with prior work, implications, transferability, and contribution(s) to the field -** Short summary of main findings; explanation of how findings and conclusions connect to, support, elaborate on, or challenge conclusions of earlier scholarship; discussion of scope of application/generalizability; identification of unique contribution(s) to scholarship in a discipline or field	13-15
**Limitations** - Trustworthiness and limitations of findings	15

Other

**Conflicts of interest** - Potential sources of influence or perceived influence on study conduct and conclusions; how these were managed	16
**Funding** - Sources of funding and other support; role of funders in data collection, interpretation, and reporting	16

*The authors created the SRQR by searching the literature to identify guidelines, reporting standards, and critical appraisal criteria for qualitative research; reviewing the reference lists of retrieved sources; and contacting experts to gain feedback. The SRQR aims to improve the transparency of all aspects of qualitative research by providing clear standards for reporting qualitative research.

For peer review only

For peer review only - http://bmjopen.bmj.com/site/about/guidelines.xhtml

**The rationale should briefly discuss the justification for choosing that theory, approach, method, or technique rather than other options available, the assumptions and limitations implicit in those choices, and how those choices influence study conclusions and transferability. As appropriate, the rationale for several items might be discussed together.

Reference:

O'Brien BC, Harris IB, Beckman TJ, Reed DA, Cook DA. **Standards for reporting qualitative research: a synthesis of recommendations.** *Academic Medicine*, Vol. 89, No. 9 / Sept 2014 DOI: 10.1097/ACM.0000000000000388

For peer review only

For peer review only - http://bmjopen.bmj.com/site/about/guidelines.xhtml

BMJ Open

What is current care for people with Long COVID in England? A qualitative interview study.

Journal:	*BMJ Open*
Manuscript ID	bmjopen-2023-080967.R1
Article Type:	Original research
Date Submitted by the Author:	05-Mar-2024
Complete List of Authors:	Sunkersing, David; University College London, Institute of Health Informatics<br>Ramasawmy, Mel ; University College London, Institute of Health Informatics<br>Alwan, Nisreen; University of Southampton, Academic unit of Primary Care and Population Sciences, Faculty of Medicine; University of Southampton and University Hospital Southampton NHS Foundation Trust, NIHR Southampton Biomedical Research Centre<br>Clutterbuck, Donna ; University of Southampton, Academic unit of Primary Care and Population Sciences, Faculty of Medicine<br>Mu, Yi ; University College London, Institute of Health Informatics<br>Horstmanshof, Kim; University College London<br>Banerjee, Amitava; University College London, Institute of Health Informatics<br>Heightman, Melissa; University College London Hospitals NHS Foundation Trust
**Primary Subject Heading**:	Health services research
Secondary Subject Heading:	Qualitative research
Keywords:	COVID-19, QUALITATIVE RESEARCH, PUBLIC HEALTH, Post-Acute COVID-19 Syndrome, Health Services

SCHOLARONE™
Manuscripts

I, the Submitting Author has the right to grant and does grant on behalf of all authors of the Work (as defined in the below author licence), an exclusive licence and/or a non-exclusive licence for contributions from authors who are: i) UK Crown employees; ii) where BMJ has agreed a CC-BY licence shall apply, and/or iii) in accordance with the terms applicable for US Federal Government officers or employees acting as part of their official duties; on a worldwide, perpetual, irrevocable, royalty-free basis to BMJ Publishing Group Ltd ("BMJ") its licensees and where the relevant Journal is co-owned by BMJ to the co-owners of the Journal, to publish the Work in this journal and any other BMJ products and to exploit all rights, as set out in our licence.

The Submitting Author accepts and understands that any supply made under these terms is made by BMJ to the Submitting Author unless you are acting as an employee on behalf of your employer or a postgraduate student of an affiliated institution which is paying any applicable article publishing charge ("APC") for Open Access articles. Where the Submitting Author wishes to make the Work available on an Open Access basis (and intends to pay the relevant APC), the terms of reuse of such Open Access shall be governed by a Creative Commons licence – details of these licences and which Creative Commons licence will apply to this Work are set out in our licence referred to above.

Other than as permitted in any relevant BMJ Author's Self Archiving Policies, I confirm this Work has not been accepted for publication elsewhere, is not being considered for publication elsewhere and does not duplicate material already published. I confirm all authors consent to publication of this Work and authorise the granting of this licence.

For peer review only

Title: What is current care for people with Long COVID in England? A qualitative interview study.

Authors: David Sunkersing[1], Mel Ramasawmy[1], Nisreen A Alwan[2], Donna Clutterbuck[2], Yi Mu[1], Kim Horstmanshof[1], Amitava Banerjee[1] and Melissa Heightman[3].

Corresponding Author: David Sunkersing (University College London, Institute of Health Informatics, London, UK; david.sunkersing@ucl.ac.uk; +44 20 8016 8424)

Author Institutions:
1. University College London, Institute of Health Informatics, London, UK
2. University of Southampton, Academic unit of Primary Care and Population Sciences, Faculty of Medicine, Southampton, Hampshire, UK
3. University College London Hospitals NHS Foundation Trust, London, London, UK

Keywords: COVID-19, QUALITATIVE RESEARCH, PUBLIC HEALTH, Post-Acute COVID-19 Syndrome, Health Services.

Word Count (Excluding Title Page, References, Figures): 4947

For peer review only

Abstract

Objective: To investigate current care for people with Long COVID in England.

Design: In-depth, semi-structured interviews with people living with Long COVID and Long COVID healthcare professionals; data analysed using thematic analysis.

Setting: NHS England (NHSE) post-COVID services in six clinics from November 2022 to July 2023.

Participants: 15 healthcare professionals and 21 people living with Long COVID currently attending or discharged (18 female; 3 male).

Results: Health professionals and people with lived experience highlighted the multifaceted nature of Long COVID, including its varied symptoms, impact on peoples' lives and the complexity involved in managing this condition. These impacts encompass physical, social, mental and environmental dimensions. People with Long COVID reported barriers in accessing primary care, as well as negative GP consultations where they felt unheard or invalidated, though some positive interactions were also noted. Peer support or support systems proved highly valuable and beneficial for individuals, aiding their recovery and well-being. Post-COVID services were viewed as spaces where overlooked voices found validation, offering more than medical expertise. Despite initial challenges, healthcare providers' increasing expertise in diagnosing and treating Long COVID has helped refine care approaches for this condition.

Conclusion: Long COVID care in England is not uniform across all locations. Effective communication, specialised expertise and comprehensive support systems are crucial. A patient-centred approach considering the unique complexities of Long COVID, including physical, mental health, social and environmental aspects is needed. Sustained access to post-COVID services is imperative, with success dependent on offering continuous rehabilitation beyond rapid recovery, acknowledging the condition's enduring impacts and complexities.

Data availability statement

Data are available on reasonable request. Anonymised data can be made available on request.

STRENGTHS AND LIMITATIONS OF THIS STUDY

- This study used in-depth, semi-structured interviews to provide a rich account of current care, from participants across NHSE commissioned post-COVID services in England.
- Voluntary participation from individuals able to access a post-COVID service could lead to an under-representation of certain respondent types.
- As this study aimed to investigate perspectives from 6 post-COVID sites, the findings may not be generalisable or representative of other post-COVID sites in England.

Introduction

Long COVID (or "Post-Acute Sequelae of SARS-CoV-2 infection" (PASC))(1,2) refers to a complex and poorly understood set of persistent symptoms that can continue for weeks, months or longer after an initial COVID-19 infection. First identified online from individuals with

For peer review only

ongoing symptoms of COVID-19, it quickly gained recognition in formal clinical channels within months.(3) Estimates suggest at least 65 million people worldwide have Long COVID, with reported symptoms including extreme fatigue, breathlessness, cognitive impairments and depression.(1,4,5) For some, Long COVID leads to prolonged recovery, severely impacting their work, daily activities and social relationships.(6) Importantly, the recognition and understanding of Long COVID have been enhanced by the crucial role played by people with the condition themselves, including through supportive peer-to-peer interactions, where valuable networks of shared experiences and coping strategies have been exchanged.(7) However, as symptoms often present heterogeneously, diagnosis and treatment are challenging.(1,4,8)

In September 2020, recognising the distinctive and persistent healthcare needs of individuals with Long COVID,(3,9) the Royal College of General Practitioners (RCGP) called for the establishment of a national network of 'post-COVID' services. In response, NHS England (NHSE) committed to rapidly expanding new and strengthened rehabilitation centres.(10) This occurred when only a minority of Clinical Commissioning Groups (CCGs) had successfully implemented post-COVID services, reflecting challenges in adapting healthcare services for people with Long COVID. In October 2020, NHSE implemented a comprehensive five-part package to bolster support for people with Long COVID.(11) This included the release of new guidance on managing Long COVID, the introduction of an online rehab service ('Your Covid Recovery'), designated Long COVID clinics/post-COVID services (to treat Long COVID symptoms, deliver holistic assessments with a multidisciplinary team, offer medical support, diagnostics and referral and rehabilitation where needed),(12) NIHR-funded research and establishing a new NHSE Long COVID task force.(12,13) These initiatives underscored the evolving understanding of Long COVID and the imperative for healthcare services to adapt rapidly to effectively support those navigating the complex and prolonged effects of the virus.

For people with suspected Long COVID, UK guidance advises beginning with a GP consultation for an initial assessment.(14) This involves discussing symptoms, ruling out underlying conditions and evaluating their impact on daily life. Referral to specialised services like rehabilitation or post-COVID services may follow if symptoms severely disrupt daily functioning, in accordance with NICE and WHO recommendations.(15,16) Despite this guidance, care for people with Long COVID can vary – and people have reported difficulties in accessing care,(17,18) as well as varied experiences in their treatment and support.(19) For healthcare professionals, the existing complexity in addressing Long COVID care is compounded by ongoing staff shortages, under-resourcing and insufficient funding.(20–22)

Currently, there is lack of in-depth, critical analyses concerning patient experiences on their ongoing care for Long COVID.(17,23,24) Similarly, the challenges of implementing effective post-COVID services contributes to the limited evidence on healthcare professionals' perspectives in experiences of managing Long COVID care.(18,25) To effectively manage and improve care for people living with Long COVID, it is crucial to evaluate current healthcare approaches for this specific patient group and their healthcare professionals. Our study aimed to address these gaps by conducting an in-depth investigation into current care within a sample of newly established post-COVID services in England. This paper presents findings from semi-structured interviews conducted with both individuals experiencing Long COVID and healthcare professionals providing Long COVID care.

For peer review only - http://bmjopen.bmj.com/site/about/guidelines.xhtml

Methods

Study Design and Setting

People living with Long COVID, either discharged or under follow up from six sites across England were invited to participate virtually over Microsoft Teams in either semi-structured interviews or focus groups (from November 2022 to July 2023). Healthcare professionals providing Long COVID care at each of the six sites were also invited to participate.

In addition to the above, the study inclusion criteria were >18 years old, English speaker and competent to provide written consent.

Recruitment of people with Long COVID

The service manager and clinical lead for each of the six post-COVID services, which were identified as part of a previous study, known as STIMULATE-ICP (Symptoms, Trajectory, Inequalities and Management: Understanding Long-COVID to Address and Transform Existing Integrated Care Pathways),(26) were asked to review their database of people living with Long COVID, including those that may have signed up to take part in research. Depending on the most appropriate site-specific means to approach people living with Long COVID we contacted (via email, via an online Long COVID group at one site (led by the post-COVID service) and/or via an internal hospital communication app at another site (MyCare application)) them to advertise the project and request their involvement. Voluntary response sampling (i.e. where participants had volunteered themselves to participate) was employed in this study to accommodate the unique challenges and vulnerabilities of people with Long COVID – and, importantly, recognising that those affected may experience limitations in time and energy. Although this sampling strategy, has limitations (including potential bias, lack of representativeness and underrepresentation of certain subgroups) we felt that this sampling strategy offered a more feasible and considerate approach for gathering valuable insights into the experiences of people with Long COVID.

Regular contact with all six post-COVID services was made throughout the data collection period to maximise participation. The principle of saturation guided the sample size, and semi-structured interviews were conducted until additional participants did not yield new information.

Recruitment of Healthcare Professionals

Apart from one site, each service manager and clinical lead additionally identified Long COVID healthcare professionals who were willing to participate in the study. We chose this approach – voluntary response sampling – as we considered it the most pragmatic and efficient method for collecting data.

In our healthcare professional interviews, we also investigated the route of individuals with Long COVID through a site's post-COVID services to understand the structure, delivery and accessibility of care of the Long COVID pathway.

Data Collection and Semi-Structured Interview

Each participant was asked to describe their experience of care, including referral, assessment, diagnosis and treatment (see supplementary materials 1 and 2 for full interview guides). For people living with Long COVID, the aim of the semi-structured interviews/focus groups was

For peer review only

to gain a greater understanding of their perception of care including any barriers to accessing care, their experience of the care pathway and any outcomes they experienced due to treatment. For healthcare professionals providing care for people living with Long COVID, we wanted to understand the delivery of Long COVID care from their perspective, including any challenges, historical or current. Semi-structured interviews/focus groups were selected as the data collection approach because they can provide comprehensive, profound, and detailed narratives of participants' experiences, viewpoints, and thoughts. They also strike a balance between flexibility and structure, allowing for an in-depth exploration of individual experiences with Long COVID while providing a framework for key topics. This approach enabled us to adapt the interview based on participants' responses, fostering a richer understanding of their perspectives and capturing nuanced information that may not have emerged in more rigidly structured interview formats. The semi-structured interviews were conducted by DS and MR via Microsoft Teams, who are both experienced qualitative researchers. Semi-structured interviews were conducted with both people with Long COVID and healthcare professionals until saturation was achieved and no new information was forthcoming.

Data Analysis

All participants in this study opted to take part in a semi-structured interview – i.e., no focus groups – between November 2022 to July 2023. All semi-structured interviews were (with participant consent) audio recorded and automatically transcribed verbatim in Microsoft Teams using the 'transcribe feature', which converts speech to a text transcript with each speaker individually separated.

An inductive, data-driven approach(27) to data analysis was undertaken by DS and MR. Firstly, each transcript was reviewed for accuracy and initial codes were generated using support from the qualitative analysis software programs ATLAS.ti (version 5.8.0)(28) and NVivo V.12.(29)

Following initial code generation, these were reviewed and further refined. The process was concluded when an agreement that the overarching themes effectively encapsulated the narratives present in the data for both people with Long COVID and Healthcare Professionals.

Three overarching themes were generated ((1) : Understanding Long COVID: Symptoms and Lived Realities, (2) Long COVID Care: Non-Specialist Service Experiences (3) Long COVID Care: Post-COVID Service Experiences) and a number of supporting, subsidiary subthemes.

Two researchers (DS and MR) met regularly to discuss the transcripts and discuss whether the overarching themes were reflective of the presented data. These findings were also discussed with AB (and patient representatives) for review. After review, there was agreement that the themes reflected the transcripts appropriately.

This approach of generating shared overarching themes for both people with Long COVID and healthcare professional perspectives was done to enable a deeper understanding of shared and divergent perspectives – an approach used in other studies.(30,31)

Patient and Public Involvement (PPI)

A PPI group (n=11) features in our regular management and oversight meetings. This group comprises individuals who either have Long COVID or provide care for someone with Long COVID. PPI members have contributed to the research components in this study, including informing the semi-structured interview design and approach. We also discussed the findings

For peer review only

of this study with our PPI to facilitate our interpretations of the data. The PPI group will continue to be involved in the dissemination of findings.

Results

A total of 21 people with Long COVID were interviewed from four of the six possible sites (10 from service a, 8 from service b, 2 from service c, 0 from service d, 1 from service e and 0 from service f). For people with Long COVID, the nature of their symptoms, privacy concerns, emotional challenges and/or ongoing medical commitments may have impacted participation.

All six post-COVID services were approached and contacted, with five services able to identify healthcare professionals willing to participate in the study. A total of 15 healthcare professionals were interviewed (5 from service a, 4 from service b, 3 from service c, 0 from service d, 1 from service e and 2 from service f).

We believe the smaller than anticipated sample size in this study can be attributed to staff shortages, existing pressures within the NHS and time availability. At one specific service, the absence of semi-structured interviews with healthcare professionals can be attributed to unforeseen circumstances, which hindered the engagement and participation of relevant personnel in the study.

Characteristics of people living with Long COVID are detailed in Table 1; Characteristics of healthcare professionals providing Long COVID care are detailed in Table 2.

We found that a generic Long COVID pathway was followed by many sites (Figure 1), although comprehensiveness of testing and/or referral ability varied, based on available staffing, other resources and local infrastructure.

[Figure 1. Long COVID Patient Pathway]

From our healthcare professional interviews, we were able to establish the service model used at each post-COVID service:

a: Face-to-Face

b: Hybrid (predominantly virtual; some aspects face-to-face)

c: Face-to-Face

d: Hybrid (dependent on patient)

e: Hybrid (dependent on patient)

f: Hybrid (dependent on patient – home visits, if necessary)

Information from post-COVID service "d" was obtained from publicly available data, as no direct interview was conducted with this site.

For peer review only

Table 1: Characteristics Of People Living With Long COVID.

Characteristic		Frequency	Percentage (%)
**Sex**	Male	3	14
	Female	18	86
**Age**	18-27	0	0
	28-37	1	5
	38-47	12	57
	48-57	6	29
	58-67	0	0
	68-77	2	10
**Ethnic Group**	White-British	12	57
	White - Any other White background	3	14
	Asian or Asian British - Indian	3	14
	Other Ethnic Groups - Any other ethnic group	2	10
	Not stated	1	5
**Languages Spoken**	English (Only)	14	67
	Multiple (Including English)	7	33
**Highest Educational Qualification**	GCSE/Equivalent	1	5
	A-Level	1	5
	Apprenticeship	1	5
	Undergraduate Degree	8	38
	Master's Degree	8	38
	PhD	1	5
	Not Stated	1	5
**Profession (Current or Prior to Covid/Long COVID (LC))**	Creative arts or design	2	10
	Education	1	5
	Engineering	1	5
	Financial	2	10
	Healthcare (Clinical Facing Role)	9	43
	Marketing, Advertising or PR	1	5

For peer review only

	Public Services or Administration	2	10
	Unemployed	1	5
	Retired	1	5
	Not Stated	1	5
**Comorbidities**	Yes	16	76
	No	4	19
	Not Stated	1	5
**LC Onset**	January - April 2020	6	29
	May - August 2020	2	10
	September - December 2020	2	10
	January - April 2021	1	5
	May - August 2021	4	19
	September - December 2021	0	0
	January - April 2022	5	24
	May - August 2022	1	5
	September - December 2022	0	0
**Post-COVID Service Attendance**	January - April 2020	0	0
	May - August 2020	2	10
	September - December 2020	3	14
	January - April 2021	3	14
	May - August 2021	0	0
	September - December 2021	2	10
	January - April 2022	1	5
	May - August 2022	4	19
	September - December 2022	5	24
	January - April 2023	1	5

For peer review only

Table 2: Characteristics of Healthcare Professionals Providing Long COVID Care.

Characteristic		Frequency	Percentage (%)
**Sex**	Male	3	20
	Female	12	80
**Age**	18-27	1	7
	28-37	5	33
	38-47	3	20
	48-57	2	13
	58-67	0	0
	68-77	0	0
	Not stated	4	27
**Ethnic Group**	White-British	6	40
	White-Any other White background	2	13
	Asian or Asian British - Indian	2	13
	Other Ethnic Groups - Any other ethnic group	2	13
	Not stated	3	20
**Profession**	Clinical Psychologist	1	7
	Clinical Trials Manager	1	7
	Doctor (Gastro)	1	7
	Exercise Physiologist	2	13
	Lead Therapist (Physio)	1	7
	Nurse	1	7
	Nurse (Service Lead)	1	7
	Occupational Health Physician & Post COVID Service (Medical Lead)	1	7

For peer review only

	Physiotherapist	1	7
	Post Covid Lead	1	7
	Rehabilitation Medicine Consultant	1	7
	Respiratory Physiotherapist	1	7
	Senior Specialist Physiotherapist	1	7
	Team Lead Post Covid Service	1	7
**Have they had Long COVID?**	Yes	3	20
	No	8	53
	Not Stated	4	27

To maintain participant anonymity, it is not possible to give further details on their roles.

For peer review only

Three overarching themes were identified and are elaborated further with example supporting quotations in boxes 1 – 3. We have refrained from attributing quotes to specific post-COVID services to avoid potential identification of specific services and participants.

Theme 1: "Understanding Long COVID: Symptoms and Lived Realities".

Within this theme, people living with Long COVID reported on several factors relating to their Long COVID experiences. The 'physical' subtheme underscores the persistent battles with fluctuating symptoms, highlighting the complexities that impact daily life (quotes 1 – 2, box 1). The 'mental health' subtheme captures the emotional toll of uncertainty, anxiety and stress related to the nature of the condition (quotes 3 – 4, box 1). The 'social' subtheme explores 'loss of identity, or self', as people living with Long COVID grapple with both understanding and support (quote 5, box 1). Lastly, the theme pays heed to the often-overlooked environmental factors, examining how the interplay between personal surroundings and the broader context can shape the Long COVID journey (quote 6, box 1). The interviews additionally established an evident impact of Long COVID on their working environment – and, for some, a lack of universal appreciation and/or adjustment from colleagues (quotes 7 – 8, box 1).

Overall, these subthemes weave a narrative that underscores the complex challenges faced by people living with Long COVID.

Box 1

Subtheme	Example Supporting Quote
Physical	1. *"I've got symptoms of that I've never experienced in a life before, you know, the aching joints, the headaches.". [Participant T, Male, Age Range 68-77]*
Physical	2. *"…the first few days, it was a lot of kind of like coughing, breathlessness, fevers, et cetera. And then kind of over the first few days starting to get kind of extreme tiredness and fatigue….". [Participant S, Female, Age Range 28-37]*
Mental Health	3. *"…there's a lot going on from a kind of stress, emotional sort of perspective.". [Participant J, Female, Age Range 38-47]*
Mental Health	4. *"…the most thing from [Long] Covid that affected us is our mental health and the lack of sleep." [Participant H, Female, Age Range 48-57*
Social	5. *"You know I'm shadow of the person I was and it's very hard to to accept that…". [Participant C, Female, Age Range 38-47].*
Environmental	6. *"So instead of going into a cafe, for example, we'll get a coffee and sit in the park because there's less risk. So I'm still trying to maintain a life, but it's completely different.". [Participant N, Female, Age Range 38-47]*
Working Environment	7. *"…and actually working online, I think is one of the really toughest things if you've got Long COVID.". [Participant A, Female, Age Range 48-57]*
Working Environment	8. *"You know they don't even give me time off for my appointments… sometimes I have to do them at work if I like. Take phone calls at work. You know it's really difficult.". [Participant E, Female, Age Range 38-47].*
Multi-dimensional	9. *"Most of the patients come with cluster of symptoms, majority fatigue, breathlessness and also muscle aches, joint pains and the brain fog is also very common. The same cognitive issues forgetfulness and some with cardiac.". [Nurse (Service Lead)].*

For peer review only

Multi-dimensional	10. *"…and physical symptoms with long COVID patients and they are often burdened with quite a lot of mental health issues just because of the nature of the illness.". [Doctor (Gastro)].*

Healthcare professionals, in their semi-structured interviews, echoed and acknowledged the multifaceted nature of Long COVID, aligning with the reports and experiences shared by people living with Long COVID in this study (quotes 9 – 10, box 1).

By addressing physical, mental, social and environmental dimensions, this theme highlights the holistic nature of care needed for people living with Long COVID navigating the complexities of the condition and underscores the importance of a comprehensive and integrated approach to their well-being.

Theme 2: "Long COVID Care: Non-Specialist Service Experiences".

This theme reflects the intricate journey that people living with Long COVID undertake within the healthcare system in non-specialist settings (i.e. outside of a post-COVID service). This theme encompasses a participant's experiences, challenges and interactions within the healthcare system, highlighting their encounters with medical consultations and also support outside of healthcare settings.

Our study found that securing an initial appointment with a GP was challenging, both in terms of contact (quote 1, box 2), but also with respect to waiting times. Several people with Long COVID described lengthy wait times for appointments and a lack of efficient continuity in care (quotes 2 – 3, box 2). Moreover, our study reports encounters from people living with Long COVID where, after securing an appointment, their GP seemed to avoid comprehensive discussions regarding the intricacies of their Long COVID and/or people reported frustrations with the service received (quotes 4 – 5, box 2). Some emphasised a sense of being rushed or their concerns being sidelined, highlighting the significance of effective communication between people living with Long COVID and healthcare professionals (quote 6, box 2). These can all further exacerbate the challenges of Long COVID management.

Despite these comments, however, some people living with Long COVID were keen to make a note of the positive aspects that their GP had brought about (quotes 7 – 8, box 2). However, for many people with Long COVID, they were especially keen to highlight the profound impact of community support networks (quotes 9 – 10, box 2). They emphasised the reassurance and validation found in shared experiences within online communities, as well as the crucial role played by friends and family in offering understanding and solidarity during the challenging journey of coping with long-lasting symptoms.

Lastly, we spoke to people with Long COVID who, though receiving some form of care outside of a post-COVID service, were told that it was limited in addressing all aspects of their Long COVID (quote 11, box 2). While their experiences from these healthcare professionals were positive, the need for dedicated resources and expertise to effectively manage the intricate aspects of Long COVID were still apparent.

Box 2

Subtheme	Example Supporting Quote

For peer review only

Perceived Lack of Support	1. *[Regarding their GP Practice] "It's impossible. You can't contact them in any way by phone or on the portal, and so you send them emails, and then no one, no one replies. They just ignore you". [Participant E, Female Age Range 38-47]*
Waiting Times	2. *"Yeah, I think that's the like having to wait to 12 weeks kind of variability on the GP's". [Participant S, Female, Age Range 28-37]*
Waiting Times	3. *"To see GP it was nightmare. I don't know if it's something to do particularly with my particular surgery...In terms of Long COVID clinic, it was in on average between three and six months.". [Participant F, Female, Age Range 48-57]*
Frustrations With Primary Care Service	4. *"… you get into the into the, into the the GP's office…and then you get 5 minutes. That's all really 5, possibly less than 10, less than 10 anyway. A lot less than 10. And they're under pressure…it's it's not good.". [Participant T, Male, Age Range 68-77]*
Frustrations With Primary Care Service	5. *"We spoke to our practice manager – they didn't even process my husband's kidney scans. They didn't even tell him that he needed it from his CT – that there's something wrong with my husband's kidneys. So you know, we've been treated so so badly. But we can't move GPs again…they're all just as bad as each other, and moving won't help us". [Participant H, Female, Age Range 48-57]*
Negative Clinical Engagements	6. *"…I was very much dismissed by the GP – she was quite unkind, actually. I think she just thought I was a bit nuts. I think she just thought its kind of PTSD, gone a bit nuts and very dismissive…". [Participant J, Female, Age Range 38-47]*
Positive Clinical Engagements	7. *"[I] was under so many people, I couldn't remember what's going on. She [the GP] had a list and she would just say, right, what's happening with this one? What's happening with that? Let's keep a track of everything. And it was really good because someone was keeping an eye out that I wasn't missing out anything or something need chasing.". [Participant E, Female, Age Range 38-47]*
Positive Clinical Engagements	8. *"And they've [regarding their clinicians] been more than amazing like I can't even tell you. They they've they've listened to me. They've cared for me when they were really worried. I had some problems with work and sick leave, and whatever my GP was like, I'm gonna call you in two weeks' time just to see how you are, you know, unnecessarily, really, but just kindness…I can't praise them highly enough.". [Participant E, Female, Age Range 38-47]*
Support Networks	9. *"So the Facebook community…that community was was amazing because you knew for the first time that it wasn't just you and it wasn't. You weren't alone. So that was incredible.". [Participant N, Female, Age Range 38-47]*
Support Networks	10. *"… there's sort of support from friends also with Long COVID or you know, and obviously friends and family members.". [Participant G, Female, Age Range 38-47]*
Clinical Challenges	11. *"The respiratory physios…were, you know, still being very nice and still being very supportive but basically saying, 'look, we we feel we've got to the end of what we can do. We're not Long COVID specialists, we're not funded for for doing Long COVID we think that any residual problems in your breathing patterns are all mixed up with autonomic dysfunction.". [Participant G, Female, Age Range 38-47]*

For peer review only

This theme particularly underscores the importance of fostering an environment where people living with Long COVID can a) contact their healthcare provider and b) openly express their experiences without fear of disbelief. Importantly, the theme sheds light on the complexities and ‘patient-provider’ dynamics of Long COVID care, emphasising the need for improved ‘patient-provider’ communication, support and more efficient pathways within the healthcare system. Finally, the significance of a robust support network, where an individual’s experiences feel validated cannot be overstated in the context of Long COVID.

Theme 3: "Long COVID Care: Post-COVID Service Experiences".

As noted in Theme 2, participants recount their initial struggles in accessing appropriate care, emphasising the importance and need for specialised post-COVID services. The establishment of dedicated services tailored to people living with Long COVID has also offered a sense of validation and recognition (quotes 1 – 2, box 3). For many people with Long COVID, these services represent more than just medical facilities; they serve as havens where individuals, perhaps previously unheard, find their voices listened to. Beyond medical reassurance, the services provide a space where the legitimacy of their symptoms and experiences are acknowledged, fostering a sense of validation and understanding that transcends merely physical symptoms. However, some people with Long COVID suggested that they expected more comprehensive or hands-on support from these post-COVID services (quote 3, box 3). Nevertheless, these services signify a commitment to understanding the complexities of Long COVID and acknowledging its lasting effects. Overall, people living with Long COVID appear to appreciate this understanding and Long COVID expertise.

Box 3

Subtheme	Example Supporting Quote
Post-COVID Service Experiences	1. *"They [post-COVID service] made me feel heard. But it wasn't until they got involved that I got all the tests [referring to medical tests and referrals]…But also, they gave me a community through their Facebook.”. [Participant L, Female, Age Range 48-57]*
Post-COVID Service Experiences	2. *[About the post-COVID service] “It's reassurance as well. And then if they find something they obviously can tell you straight away. You don't need to go and look for for something, and then it's just even acknowledgement of of somebody understand you. And and know what what is going in your body and you are not on your own…so that's very important.”. [Participant F, Female, Age Range 48-57]*
Post-COVID Service Experiences	3. *“Yeah, I feel like I was a bit disappointed with my like long COVID clinic. It was just like a 60 minute phone call.”. [Participant S, Female, Age Range 28-37]*
Post-COVID Service Experiences (Healthcare Professional (HCP) Perspective)	4. *“I think everybody's learning from each other, but no, not not haven't really kind of accessed like a formal training day. Like I've dipped into webinars and bits of things that I've seen that people are colleagues. I know that are doing that I'm kind of interested in.”. [Physiotherapist]*
Post-COVID Service Experiences (HCP Perspective)	5. *“The psychology offer could maybe be a bit stronger. I mean, it's quite good that we even consider it and we have psychologists in, but we just haven't been able to fill the role and then issues with space and*

	staffing. Again, it's just something that's fallen to the wayside.”. [Doctor (Gastro)].
Post-COVID Service Experiences (HCP Perspective)	6. *“They didn't have the capacity to take on any of this work. So we we wanted to have very much a multispecialty approach to managing long COVID. But our respiratory physicians and cardiologists didn't have the time available to to join the team. So the challenges have been that from a medical perspective, it's very much fallen solely on me.”. [Rehabilitation Medicine Consultant].*
Post-COVID Service Experiences (HCP Perspective)	7. *“…working with people with long COVID can feel quite daunting unless you've seen a lot of it. And the confidence that comes with kind of managing that clinical uncertainty of what's in front of you only comes through with experience.”. [Physiotherapist].*
Post-COVID Service Experiences (HCP Perspective)	8. *“…a key barrier…is how we work across the integrated care system. So we're all on different electronic records and the information governance agreements extend to some things and not others. We don't yet have sight of how our patients are doing. You know, we assess them in clinic and we'll send them to rehab, but we don't really get hearing about their outcomes and that that is that is problematic both in terms of how we assess whether what we're doing is effective and and how we coordinate care, but it also limits us in our delivery.”. [Physiotherapist].*
Post-COVID Service Experiences (HCP Perspective)	9.*“Diagnostics wise, I think at the beginning we did a lot of tests because we really didn't know what was going on. And as we've grown our clinical confidence and expertise…the number of tests reduced…”. [Post COVID Lead].*

The semi-structured interviews with healthcare providers shed light on the experiences they faced while operating a Long COVID Service. Across all sites, the healthcare professionals confronted a multitude of obstacles, with a significant portion stemming from the pervasive clinical uncertainty surrounding Long COVID and the absence of standardised training programs. As a result, many healthcare providers had to rely on self-directed learning to stay updated and effective in their roles (quote 4, box 3). Furthermore, the persistent staffing and capacity issues within these specialised services meant healthcare teams often could not deliver the best possible care for people dealing with the long-lasting effects of COVID-19 (quotes 5 – 6, box 3). The semi-structured interviews additionally highlighted the clinical uncertainty surrounding the best management practices for Long COVID (quote 7, box 3), indicating a pressing need for further research and guidance (e.g., by having models of care commissioned to achieve this) in this emerging field. Healthcare professionals also underscored the difficulties of navigating integrated care systems (quote 8, box 3), emphasising the need for improved collaboration and coordination among various healthcare entities to better address the multifaceted needs of people with Long COVID.

Despite these challenges, for many post-COVID services, while initially there were substantial clinical unknowns, there was now a sense of greater clinical expertise and confidence, resulting in more refined approaches in Long COVID care (quote 9, box 3).

Discussion

For peer review only

In our study, which examined perspectives from people with lived experience of Long COVID and healthcare professionals, we found three overarching themes: (1) Understanding Long COVID: Symptoms and Lived Realities, (2) Long COVID Care: Non-Specialist Service Experiences and (3) Long COVID Care: Post-COVID Service Experiences.

Our first theme resonates with contemporary research that underscores the intricate, individual and varied nature of the Long COVID journey. Our findings from people with Long COVID align with studies that have emphasised the diverse range of symptoms of this condition,(32) highlighting the importance of personalised care models that account for the multifaceted dimensions of Long COVID. The emphasis from people living with Long COVID on mental health aligns with other research,(33) which showed the psychological and sometimes traumatic toll of living with Long COVID, further stressing the need for integrated mental health support as part of comprehensive Long COVID management strategies.

Our study additionally highlighted that the impact of Long COVID on people can extend well beyond its physiological manifestations, significantly affecting their immediate surroundings and (if applicable), their working environments. This study's findings corroborate with existing literature,(34,35) underlining the interplay between people living with Long COVID and the broader contexts in which they reside and work. The disruptions observed in social circles and routines align with the observations made in other studies,(36,37) highlighting the persistent challenges people living with Long COVID face as they navigate the unpredictable nature of Long COVID symptoms. Our study additionally echoes the experiences of those with cognitive impairments and reduced energy levels from Long COVID, resonating with other findings(38,39) that have documented the diverse impacts of Long COVID on professional functioning and adjustments required in work environments.(38,39) Notably, our findings indicated that the uncertainty caused by the lack of an effective treatment along with debilitating symptoms and the potential disruption across various aspects of daily life, makes living with long COVID especially challenging.

The healthcare professional's perspectives of symptoms resonated with those of the patients, highlighting a shared understanding of the complex nature of Long COVID – and the multifaceted impact it can cause. Overall, these two perspectives underline the urgent need for supportive structures that acknowledge the multifaceted dimensions of Long COVID, both within the personal and professional spheres, ensuring comprehensive and responsive care.

Our second theme, which exclusively examined the perspectives of people with Long COVID, underscored their frustrations with the accessibility and responsiveness of their GP practice and described challenges in contacting them and/or receiving a response. Prolonged waiting times were frequently reported in our semi-structured interviews, exacerbating their difficulties in accessing care. These experiences align with literature(40,41) and highlight the need for improved access to healthcare services for Long COVID patients and adequate support structures for managing this complex condition within primary care settings.

The experiences shared by people living with Long COVID in our study highlight a disconcerting circumstance wherein some GPs and other healthcare professionals seemingly avoid engaging in thorough discussions about the complexities of Long COVID. The people living with Long COVID consistently express feelings of being hurried through appointments or having their concerns dismissed. These findings align with emerging observations in the literature,(42–44) reflecting the challenge faced by healthcare providers in addressing the

For peer review only

nuanced needs of people living with Long COVID within the constraints of limited appointment times. Such negative interactions can significantly impact mental health, impeding the overall well-being and recovery of those navigating the complexities of their condition.(45)

Similar issues in patient-provider interactions within the context of Long COVID care have been described in other studies globally, emphasising the pressures on healthcare systems exacerbated by the pandemic. (44,46,47) The existing backlog of waiting lists, compounded by the strains of the COVID-19 pandemic, have strained healthcare resources, possibly contributing to the reported challenges in addressing Long COVID comprehensively within primary care settings. However, it is important to consider whether an individual's expectations align with the practical capacities of clinicians. Nevertheless, overall, these dynamics emphasise the urgent need for tailored approaches to address Long COVID within healthcare systems and fostering improved communication between both parties to align expectations with achievable outcomes.

Our study found that establishment and use of Long COVID-specific online groups (both patient-led and provider-led) emerge as a noteworthy peer-support mechanism, facilitating connection and information-sharing among people living with Long COVID. These findings resonate with current literature, highlighting the value of online platforms in fostering a sense of community and understanding among people with Long COVID. The positive impact observed in our study aligns with the observations from other studies(48,49) where they have emphasised the therapeutic benefits of peer support networks in Long COVID management, acknowledging their role in reducing feelings of isolation and offering practical insights. The potential for these digital spaces to serve as outlets for shared experiences and coping strategies reflects a promising avenue for people living with Long COVID navigating the complexities of this condition, affirming the significance of such platforms within the broader landscape of patient-centred care. Nevertheless, while these online groups can be an avenue for peer support, given their potential for a source of conflicting information and misinformation, there remains a demand for accurate, up-to-date information to ensure individuals have reliable and current data which could support medical management.(50,51)

Lastly, a lack of clinical understanding of Long COVID among healthcare providers, as reported (or experienced) by people with Long COVID, aligns with previous findings.(52) This underscores the persistent challenge in understanding Long COVID, signifying the need for continued research and standardised approaches to improve patient care and outcomes in this complex condition.

The third theme aligns with discussions in the literature surrounding specialised post-COVID services. This theme was divided into two perspectives: that of people with Long COVID and healthcare professionals providing care within the post-COVID service.

In our interviews, we found that diverse models of post-COVID services were being used, where some operate primarily face-to-face (post-COVID services a and c) and others adopt hybrid approaches (post-COVID services b, d, e, and f), suggesting flexibility to tailor care to individual needs. The hybrid nature of post-COVID services potentially reflects a responsiveness to patient requirements, ranging from entirely virtual to incorporating in-person components such as home visits when necessary. Additionally, considerations such as pandemic-related infection control measures and staffing constraints within these post-COVID

For peer review only

service models may have influenced preference and practicality of a virtual service for certain sites and individuals.(53)

For people with Long COVID, the positive perception of expert care within these clinics echoes the sentiments expressed by recent research(54) stressing the importance of multidisciplinary care teams in addressing the complex and varied symptomatology of Long COVID. While immediate clinical improvement may not always be observed, many people living with Long COVID described the transformative nature of their interactions with healthcare professionals in these specialised clinics. Importantly, for many, it was a place where they were given time to discuss their symptoms and where they were validated and understood. This finding agrees with existing literature,(55,56) reinforcing the crucial role of empathy in health and social care professionals. We underscore its inherent importance in any service, with a particular emphasis on its heightened relevance in situations marked by clinical uncertainty. Further, our findings resonate with existing literature that reflects the pivotal role that post-COVID services play in addressing the complex needs of people living with Long COVID. Our study's insights also align with recommendations from previous studies(57,58) where an emphasis on the importance of multidisciplinary care teams and specialised expertise in tackling the intricate symptomatology of Long COVID was given.

For the virtual post-COVID services, drawbacks potentially include limited medical examination, challenges in effective communication and the risk of exacerbating health inequalities due to disparities in technology access.(59) The lack of hands-on assessments in virtual care may lead to delays in diagnosis or treatment and the digital nature of interactions may limit emotional connections. Additionally, the digital divide could exclude individuals with limited access to technology, hindering inclusivity in these support networks.(60) Addressing these concerns is crucial for ensuring a comprehensive and equitable approach to managing the complexities of Long COVID.

For healthcare professionals working within the post-COVID service, they reported clinical uncertainty, 'learning on the job' and a lack of resources – issues all previously noted in other studies.(52,61) Nevertheless, given the largely positive reviews of the post-COVID services, there was a sense that the healthcare professionals in the post-COVID service were able to align their clinical approach with the diverse expectations of those under their care. Striking a balance between patient expectations and the intricacies of their conditions has been well reported in the literature – an essential approach for reducing patient dissatisfaction and potentially improving health outcomes.(52,62) Although there remains clinical uncertainty with respect to Long COVID, efforts to address best practices towards assessing, treating and rehabilitating have been made with a training programme developed by NHSE, which launched in June 2023.(63)

We agree with suggestions that as the body of Long COVID evidence and diagnostic criteria evolves, there may be a need to revise referral criteria for specialist services to ensure the most efficient allocation of resources(52,64).(52,64) Similarly, self-referral to post-COVID clinics could be effective if these clinics could adequately manage the demand, which hinges on factors such as staffing and other available resources. However, the viability of such an approach is likely to vary since it heavily relies on the specific capabilities of each post-COVID service and may not be universally applicable.

Limitations

For peer review only

The underrepresentation of males is a potential limitation, which might limit the comprehensiveness of our findings and hinder a complete understanding of gender-specific experiences. The overrepresentation of White British/White ethnicities potentially limits the generalisability of our findings to more diverse populations, particularly as evidence suggests that ethnic minorities have been disproportionately impacted by COVID-19(65) and Long COVID has been reported across various ethnic groups.(66) Additionally, the focus on English-speaking participants may exclude those who primarily communicate in other languages, potentially overlooking unique perspectives within linguistically diverse communities. The high representation of individuals with higher education levels and healthcare employment introduces a potential bias, as experiences and access to healthcare resources may differ for those with varied educational backgrounds and occupational statuses.(67) Further, the limited age range of participants may not fully capture the experiences of younger or older individuals with Long COVID. Voluntary participation and its potential to introduce selection bias is a further limitation.(68) As this study recruited people attending post-COVID services only, this excludes opinions and experiences of those who could not access these services. Nevertheless, our sample's demographics appear to be aligned with the utilisation of post-COVID services by age, gender, ethnicity in England, as recorded by NHSE.(69)

Overall, although this population is not necessarily representative of the populations who have developed Long COVID at high rates, (1) these findings offer a particular lens through which to understand Long COVID, providing valuable insights into the experiences of this specific subgroup.

Conclusions

By delving into the personal narratives and experiences of people living with Long COVID, we have uncovered several examples of good practice, but also challenges within the realm of healthcare provision. These findings underscore the urgent need for a patient-centred approach that considers the unique complexities of Long COVID, encompassing its physical manifestations in addition to its impact on mental health, social and environmental aspects. Further, this study emphasised the pivotal role of effective communication, specialised expertise and comprehensive support systems. Our study advocates for the integration of these insights into the development of tailored interventions and policies that can holistically address the needs of people living with Long COVID, ultimately enhancing the quality of care and improving the lives of those with this condition. Though not without its challenges, ensuring continued, sustainable access to specialised care for people with Long COVID requires a thoughtful and enduring commitment to address the multifaceted needs of those affected. The success of post-COVID services hinges on the ability to offer ongoing rehabilitation that extends beyond immediate recovery, acknowledging the long-term impacts and complexities associated with this condition.

Footnotes

Participant Consent for Publication

Not Applicable.

Ethics Approval

For peer review only

This study involves human participants and ethical approval (IRAS Project ID: 303958) was granted by the Health Research Authority (HRA) and South Central—Berkshire Research Ethics Committee (REC Reference: 22/SC/0047). All participants gave informed written consent to participate in the study before taking part.

Funding

This research was funded by NIHR (COV-LT2-0043) as part of the STIMULATE-ICP study. The funders had no role in the design of the study; in the collection, analyses, or interpretation of data; in the writing of the manuscript; or in the decision to publish the results.

Acknowledgements

Our sincere thanks to all participants in this study for their time and the information they provided.

This study is part of the STIMULATE-ICP study. The STIMULATE-ICP Consortium: Amitava Banerjee, Elizabeth Murray, Hakim-Moulay Dehbi, Hugh Montgomery, Sarah Clegg, Henry Goodfellow, Mel Ramasawmy, Yi Mu, Sampath Weerakkody, Ileana Selejan, David Sunkersing, Ashkan Dashtban, Paula Lorgelly, Melissa Heightman, Toby Hillman, Emma Wall, Caroline Leigh-Watkins, Denise Forshaw, Gordon Prescott, Gail Allsopp, Mark Gabbay, Gregory Lip, Dan Cuthbertson, Dan Wootton, Nefyn Williams, Mike G Crooks, Angela Green, Christina van der Feltz-Cornelis, Jenny Sweetman, Han-I Wang, Natalie Smith, Kamlesh Khunti, Lauren O'Mahoney, Rachael Evans, William D Strain, Rachel Botell, Nisreen Alwan, Donna Clutterbuck, Marija Pantelic, Chris Robson, Mike Brady, Rajarshi Banerjee, Cat Kelly, Angela Barone, Johannes Alberts, Rob Suriano, Lyth Hishmeh, Emily Attree, Jasmine Hayer, Rita Mallinson Cookson, Rachel Hext, Andrew Williams, Rachel Williams, Mag Leahy, Antony Loveless, Clare Loveless, Kim Horstmanshof, Ewen Brennan, Amanpreet Sarna, Manuel Gomes, Andrew Clegg, Valerio Benedetto, Melissa Dalton, Fauzia Begum, Fidan Turk, Michele Pansini, Anu Chandra, Gemma Clunie, Dominic Crocombe, Shane McAuliffe, Michelle Quaye, Farhat Gilani, Yusuf Jaami, and Nikki Cleary. An up-to-date version of Consortium members can be found on https://www.stimulate-icp.org/team. STIMULATE-ICP can be contacted at: info@stimulate-icp.org.

Disclaimer

The views expressed in this publication are those of the author(s) and not necessarily those of the National Institute for Health and Social Care Research or the Department for Health and Social Care.

Competing Interests

AB has received research funding from NIHR, British Medical Association, Astra Zeneca, National Institute of Aging and European Union. AB is a Trustee of Long COVID SoS. NAA has contributed in an advisory capacity to the World Health Organisation and the European Union Commission's Expert Panel on effective ways of investing in health meetings in relation to post-COVID-19 condition. MH is National Specialty Advisor for Long Covid, NHS England.

All other authors report no competing interests.

For peer review only

Supplementary Materials

1. Supplementary Material 1 - Patient Interview Schedule
2. Supplementary Material 2 - HCP Interview Schedule

Word Count (Excluding Tables and References): 4947.

Author Contributions: AB, MH and the STIMULATE-ICP Consortium conceived and designed the study. DS and MR conducted semi-structured interviews with both people with Long COVID and healthcare professionals. DS conducted data analysis and drafted the paper. MR, NA, DC, YM, KH, AB and MH provided constructive feedback on drafts of the manuscript and approved the final version. DS is the guarantor.

References:

1. Davis HE, McCorkell L, Vogel JM, Topol EJ. Long COVID: major findings, mechanisms and recommendations. Nature Reviews Microbiology 2023 21:3 [Internet]. 2023 Jan 13 [cited 2023 Sep 19];21(3):133–46. Available from: https://www.nature.com/articles/s41579-022-00846-2

2. Parker AM, Brigham E, Connolly B, McPeake J, Agranovich A V., Kenes MT, et al. Addressing the post-acute sequelae of SARS-CoV-2 infection: a multidisciplinary model of care. Lancet Respir Med [Internet]. 2021 Nov 1 [cited 2023 Sep 19];9(11):1328–41. Available from: https://pubmed.ncbi.nlm.nih.gov/34678213/

3. Callard F, Perego E. How and why patients made Long Covid. Soc Sci Med. 2021 Jan 1;268:113426.

4. Koc HC, Xiao J, Liu W, Li Y, Chen G. Long COVID and its Management. Int J Biol Sci [Internet]. 2022 [cited 2023 Sep 19];18(12):4768–80. Available from: https://pubmed.ncbi.nlm.nih.gov/35874958/

5. Walker S, Goodfellow H, Pookarnjanamorakot P, Murray E, Bindman J, Blandford A, et al. Impact of fatigue as the primary determinant of functional limitations among patients with post-COVID-19 syndrome: a cross-sectional observational study. BMJ Open [Internet]. 2023 Jun 1 [cited 2023 Sep 19];13(6):e069217. Available from: https://bmjopen.bmj.com/content/13/6/e069217

6. Aghaei A, Zhang R, Taylor S, Tam CC, Yang CH, Li X, et al. Impact of COVID-19 symptoms on social aspects of life among female long haulers: A qualitative study. Res Sq. 2022 Feb 10;

7. Mullard JCR, Kawalek J, Parkin A, Rayner C, Mir G, Sivan M, et al. Towards evidence-based and inclusive models of peer support for long covid: A hermeneutic systematic review. Soc Sci Med. 2023 Mar 1;320:115669.

8. Mantovani A, Morrone MC, Patrono C, Santoro MG, Schiaffino S, Remuzzi G, et al. Long Covid: where we stand and challenges ahead. Cell Death & Differentiation 2022 29:10 [Internet]. 2022 Sep 7 [cited 2023 Sep 19];29(10):1891–900. Available from: https://www.nature.com/articles/s41418-022-01052-6

9. Garner P. Paul Garner: For 7 weeks I have been through a roller coaster of ill health, extreme emotions, and utter exhaustion - The BMJ [Internet]. 2020 [cited 2024 Feb 3]. Available from: https://blogs.bmj.com/bmj/2020/05/05/paul-garner-people-who-have-a-more-protracted-illness-need-help-to-understand-and-cope-with-the-constantly-shifting-bizarre-symptoms/

10. BBC. Coronavirus: “Long Covid” patients need treatment programme, doctors say - BBC News [Internet]. 2020 [cited 2024 Feb 4]. Available from: https://www.bbc.co.uk/news/health-54031587

For peer review only

11. NHS England. NHS England » NHS to offer 'long covid' sufferers help at specialist centres [Internet]. 2020 [cited 2024 Feb 4]. Available from: https://www.england.nhs.uk/2020/10/nhs-to-offer-long-covid-help/

12. NHS England. National commissioning guidance for post COVID services [Internet]. 2022 [cited 2023 Oct 9]. Available from: https://www.england.nhs.uk/publication/national-commissioning-guidance-for-post-covid-services/

13. NICE. COVID-19 rapid guideline: managing the long-term effects of COVID-19 [Internet]. 2020 [cited 2023 Oct 9]. Available from: https://www.nice.org.uk/guidance/ng188

14. NHS. Long-term effects of COVID-19 (long COVID) - NHS [Internet]. 2024 [cited 2024 Feb 22]. Available from: https://www.nhs.uk/conditions/covid-19/long-term-effects-of-covid-19-long-covid/

15. Scenario: Management | Management | Long-term effects of coronavirus (long COVID) | CKS | NICE [Internet]. [cited 2024 Feb 22]. Available from: https://cks.nice.org.uk/topics/long-term-effects-of-coronavirus-long-covid/management/management/

16. World Health Organization. Guideline Clinical management of COVID-19: living guideline. 2023 [cited 2024 Feb 22]; Available from: http://apps.who.int/bookorders.

17. Baz SA, Fang C, Carpentieri JD, Sheard L. 'I don't know what to do or where to go'. Experiences of accessing healthcare support from the perspectives of people living with Long Covid and healthcare professionals: A qualitative study in Bradford, UK. Health Expectations [Internet]. 2023 Feb 1 [cited 2024 Feb 23];26(1):542–54. Available from: https://onlinelibrary.wiley.com/doi/full/10.1111/hex.13687

18. MacPherson K, Cooper K, Harbour J, Mahal D, Miller C, Nairn M. Experiences of living with long COVID and of accessing healthcare services: a qualitative systematic review. BMJ Open [Internet]. 2022 Jan 1 [cited 2024 Feb 23];12(1):e050979. Available from: https://bmjopen.bmj.com/content/12/1/e050979

19. BMA. Addressing the health challenge of long COVID Executive summary. 2022;

20. BMA. COVID-19: Impact of the pandemic on healthcare delivery [Internet]. 2023 [cited 2024 Feb 23]. Available from: https://www.bma.org.uk/advice-and-support/covid-19/what-the-bma-is-doing/covid-19-impact-of-the-pandemic-on-healthcare-delivery

21. Limb M. Staff and patients at risk from underinvestment in health and social care, says England's regulator. BMJ [Internet]. 2022 Oct 21 [cited 2024 Feb 23];379. Available from: https://www.bmj.com/content/379/bmj.o2537

22. Waitzman E. Staff shortages in the NHS and social care sectors [Internet]. 2022 [cited 2024 Feb 23]. Available from: https://lordslibrary.parliament.uk/staff-shortages-in-the-nhs-and-social-care-sectors/

23. Baz S, Fang C, Carpentieri J, Sheard L. Understanding the lived experience of Long Covid: A rapid literature review. 2021 [cited 2024 Feb 23]; Available from: https://www.ucl.ac.uk/covid-19-longitudinal-health-wellbeing/convalescence-long-covid-

24. Ladds E, Rushforth A, Wieringa S, Taylor S, Rayner C, Husain L, et al. Persistent symptoms after Covid-19: qualitative study of 114 "long Covid" patients and draft quality principles for services. BMC Health Serv Res [Internet]. 2020 Dec 1 [cited 2024 Feb 23];20(1). Available from: /pmc/articles/PMC7750006/

25. McNabb KC, Bergman AJ, Smith-Wright R, Seltzer J, Slone SE, Tomiwa T, et al. "It was almost like it's set up for people to fail" A qualitative analysis of experiences and unmet supportive needs of people

For peer review only

with Long COVID. BMC Public Health [Internet]. 2023 Dec 1 [cited 2024 Feb 23];23(1):1–12. Available from: https://bmcpublichealth.biomedcentral.com/articles/10.1186/s12889-023-17033-4

26. Forshaw D, Wall EC, Prescott G, Dehbi HM, Green A, Attree E, et al. STIMULATE-ICP: A pragmatic, multi-centre, cluster randomised trial of an integrated care pathway with a nested, Phase III, open label, adaptive platform randomised drug trial in individuals with Long COVID: A structured protocol. PLoS One [Internet]. 2023 Feb 1 [cited 2023 Sep 19];18(2). Available from: https://pubmed.ncbi.nlm.nih.gov/36791116/

27. Schreier M. Qualitative Content Analysis in Practice. SAGE Publications. 2012. 24–25 p.

28. ATLAS.ti. Scientific Software Development GmbH; 2023.

29. Lumivero. NVivo (Version 12). 2017.

30. Hassan L, Edge D, Senior J, Shaw J. Staff and patient perspectives on the purpose of psychotropic prescribing in prisons: care or control? Gen Hosp Psychiatry. 2013 Jul 1;35(4):433–8.

31. Insua-Summerhays B, Hart A, Plummer E, Priebe S, Barnicot K. Staff and patient perspectives on therapeutic engagement during one-to-one observation. J Psychiatr Ment Health Nurs [Internet]. 2018 Nov 1 [cited 2024 Feb 9];25(9–10):546–57. Available from: https://onlinelibrary.wiley.com/doi/full/10.1111/jpm.12497

32. Michelen M, Manoharan L, Elkheir N, Cheng V, Dagens A, Hastie C, et al. Characterising long COVID: a living systematic review. BMJ Glob Health [Internet]. 2021 Sep 1 [cited 2023 Sep 19];6(9):e005427. Available from: https://gh.bmj.com/content/6/9/e005427

33. Houben-Wilke S, Goërtz YMJ, Delbressine JM, Vaes AW, Meys R, Machado FVC, et al. The Impact of Long COVID-19 on Mental Health: Observational 6-Month Follow-Up Study. JMIR Ment Health 2022;9(2):e33704 https://mental.jmir.org/2022/2/e33704 [Internet]. 2022 Feb 24 [cited 2023 Sep 19];9(2):e33704. Available from: https://mental.jmir.org/2022/2/e33704

34. Ziauddeen N, Gurdasani D, O'Hara ME, Hastie C, Roderick P, Yao G, et al. Characteristics and impact of Long Covid: Findings from an online survey. PLoS One. 2022;17(3 March).

35. Suran M. Long COVID Linked With Unemployment in New Analysis. Vol. 329, JAMA. 2023.

36. Samper-Pardo M, Oliván-Blázquez B, Magallón-Botaya R, Méndez-López F, Bartolomé-Moreno C, León-Herrera S. The emotional well-being of Long COVID patients in relation to their symptoms, social support and stigmatization in social and health services: a qualitative study. BMC Psychiatry [Internet]. 2023 Dec 1 [cited 2023 Sep 19];23(1):1–13. Available from: https://bmcpsychiatry.biomedcentral.com/articles/10.1186/s12888-022-04497-8

37. Schmachtenberg T, Müller F, Kranz J, Dragaqina A, Wegener G, Königs G, et al. How do long COVID patients perceive their current life situation and occupational perspective? Results of a qualitative interview study in Germany. Front Public Health [Internet]. 2023 [cited 2023 Sep 19];11. Available from: /pmc/articles/PMC10034079/

38. De Luca R, Bonanno M, Calabrò RS. Psychological and Cognitive Effects of Long COVID: A Narrative Review Focusing on the Assessment and Rehabilitative Approach. J Clin Med [Internet]. 2022 Nov 1 [cited 2023 Sep 19];11(21). Available from: https://pubmed.ncbi.nlm.nih.gov/36362782/

39. Zahouani I, Desmeules F, Perreault K, Campeau-Lecours A, Best K, Beaulieu-Bonneau S, et al. Physical and cognitive impairments in people suffering from long COVID: protocol for a longitudinal population-based cohort study. BMJ Open [Internet]. 2023 [cited 2023 Sep 19];13:64054. Available from: http://bmjopen.bmj.com/

For peer review only

40. Goyal DK, Mansab F, Naasan AP, Iqbal A, Millar C, Franklin G, et al. Restricted access to the NHS during the COVID-19 pandemic: Is it time to move away from the rationed clinical response? The Lancet Regional Health - Europe [Internet]. 2021 Sep 1 [cited 2024 Feb 14];8:100201. Available from: http://www.thelancet.com/article/S2666776221001782/fulltext

41. McIntyre D, Chow CK. Waiting Time as an Indicator for Health Services Under Strain: A Narrative Review. Inquiry (United States) [Internet]. 2020 Apr 30 [cited 2024 Feb 14];57. Available from: https://journals.sagepub.com/doi/10.1177/0046958020910305

42. Purewal S, Byers K, Jamieson K, Zolfaghari N. The Conversation. 2023. People with long COVID continue to experience medical gaslighting more than 3 years into the pandemic.

43. Anonymous. As a doctor with long covid, I feel abandoned by the NHS. Vol. 380, BMJ (Clinical research ed.). 2023.

44. Au L, Capotescu C, Eyal G, Finestone G. Long covid and medical gaslighting: Dismissal, delayed diagnosis, and deferred treatment. SSM Qualitative research in health. 2022 Dec;2:100167.

45. Eriksen AA, Fegran L, Fredwall TE, Larsen IB. Patients' negative experiences with health care settings brought to light by formal complaints: A qualitative metasynthesis. J Clin Nurs [Internet]. 2023 Sep 1 [cited 2024 Feb 15];32(17–18):5816–35. Available from: https://onlinelibrary.wiley.com/doi/full/10.1111/jocn.16704

46. Hussain FA. Facilitating care: a biopsychosocial perspective on long COVID. British Journal of General Practice [Internet]. 2022 Jan 1 [cited 2023 Sep 19];72(714):30–1. Available from: https://bjgp.org/content/72/714/30

47. Kingstone T, Taylor AK, O'Donnell CA, Atherton H, Blane DN, Chew-Graham CA. Finding the "right" GP: a qualitative study of the experiences of people with long-COVID. BJGP Open [Internet]. 2020 Dec 1 [cited 2023 Sep 19];4(5):1–12. Available from: https://bjgpopen.org/content/4/5/bjgpopen20X101143

48. Russell D, Spence NJ, Chase JAD, Schwartz T, Tumminello CM, Bouldin E. Support amid uncertainty: Long COVID illness experiences and the role of online communities. SSM - Qualitative Research in Health. 2022 Dec 1;2:100177.

49. Day HLS. Exploring Online Peer Support Groups for Adults Experiencing Long COVID in the United Kingdom: Qualitative Interview Study. J Med Internet Res [Internet]. 2022 May 1 [cited 2023 Sep 19];24(5). Available from: /pmc/articles/PMC9128729/

50. Brown K, Yahyouche A, Haroon S, Camaradou J, Turner G. Long COVID and self-management. The Lancet. 2022 Jan;399(10322):355.

51. Aiyegbusi OL, Hughes SE, Turner G, Rivera SC, McMullan C, Chandan JS, et al. Symptoms, complications and management of long COVID: a review. J R Soc Med. 2021 Sep 15;114(9):428–42.

52. Ladds E, Rushforth A, Wieringa S, Taylor S, Rayner C, Husain L, et al. Developing services for long COVID: lessons from a study of wounded healers. Clin Med (Lond) [Internet]. 2021 Jan 1 [cited 2023 Sep 19];21(1):59–65. Available from: https://pubmed.ncbi.nlm.nih.gov/33479069/

53. Chung-Lee L, Catallo C. A new approach to digital health? Virtual COVID-19 care: A scoping review. Digit Health [Internet]. 2023 Jan 1 [cited 2024 Mar 1];9. Available from: /pmc/articles/PMC9926398/

54. Parkin A, Davison J, Tarrant R, Ross D, Halpin S, Simms A, et al. A Multidisciplinary NHS COVID-19 Service to Manage Post-COVID-19 Syndrome in the Community. J Prim Care Community Health [Internet]. 2021 [cited 2023 Sep 19];12. Available from: https://pubmed.ncbi.nlm.nih.gov/33880955/

For peer review only

55. Jeffrey D. Empathy, sympathy and compassion in healthcare: Is there a problem? Is there a difference? Does it matter? J R Soc Med [Internet]. 2016 Dec 1 [cited 2024 Feb 15];109(12):446–52. Available from: https://pubmed.ncbi.nlm.nih.gov/27923897/

56. Moudatsou M, Stavropoulou A, Philalithis A, Koukouli S. The Role of Empathy in Health and Social Care Professionals. Healthcare [Internet]. 2020 [cited 2024 Feb 15];8(1). Available from: /pmc/articles/PMC7151200/

57. Greenhalgh T, Sivan M, Delaney B, Evans R, Milne R. Long covid—an update for primary care. BMJ [Internet]. 2022 Sep 22 [cited 2023 Sep 19];378. Available from: https://www.bmj.com/content/378/bmj-2022-072117

58. Nalbandian A, Sehgal K, Gupta A, Madhavan M V., McGroder C, Stevens JS, et al. Post-acute COVID-19 syndrome. Nature Medicine 2021 27:4 [Internet]. 2021 Mar 22 [cited 2023 Sep 19];27(4):601–15. Available from: https://www.nature.com/articles/s41591-021-01283-z

59. Yao R, Zhang W, Evans R, Cao G, Rui T, Shen L. Inequities in Health Care Services Caused by the Adoption of Digital Health Technologies: Scoping Review. J Med Internet Res [Internet]. 2022 Mar 1 [cited 2024 Mar 1];24(3). Available from: /pmc/articles/PMC8981004/

60. Hernandez K, Roberts T. Leaving No One Behind in a Digital World. K4D [Internet]. 2018 [cited 2024 Mar 1]; Available from: https://assets.publishing.service.gov.uk/media/5c178371ed915d0b8a31a404/Emerging_Issues_LNOBDW_final.pdf

61. O'Hare AM, Vig EK, Iwashyna TJ, Fox A, Taylor JS, Viglianti EM, et al. Complexity and Challenges of the Clinical Diagnosis and Management of Long COVID. JAMA Netw Open [Internet]. 2022 Nov 1 [cited 2024 Feb 15];5(11):e2240332–e2240332. Available from: https://jamanetwork.com/journals/jamanetworkopen/fullarticle/2798146

62. Rushforth A, Ladds E, Wieringa S, Taylor S, Husain L, Greenhalgh T. Long Covid – The illness narratives. Soc Sci Med. 2021 Oct 1;286:114326.

63. NHS England. Post COVID Services [Internet]. 2023 [cited 2023 Oct 10]. Available from: https://www.e-lfh.org.uk/programmes/post-covid-services/

64. Devereaux A, Yang H, Seda G, Sankar V, Maves RC, Karanjia N, et al. Optimizing Scarce Resource Allocation During COVID-19: Rapid Creation of a Regional Health-Care Coalition and Triage Teams in San Diego County, California. Disaster Med Public Health Prep [Internet]. 2022 Feb 10 [cited 2023 Sep 19];16(1):321–7. Available from: https://www.cambridge.org/core/journals/disaster-medicine-and-public-health-preparedness/article/optimizing-scarce-resource-allocation-during-covid19-rapid-creation-of-a-regional-healthcare-coalition-and-triage-teams-in-san-diego-county-california/2C5495005A8CF15F4971884A2B80E578

65. Kirby T. Evidence mounts on the disproportionate effect of COVID-19 on ethnic minorities. Lancet Respir Med [Internet]. 2020 Jun 1 [cited 2024 Feb 10];8(6):547–8. Available from: http://www.thelancet.com/article/S2213260020302289/fulltext

66. Khullar D, Zhang Y, Zang C, Xu Z, Wang F, Weiner MG, et al. Racial/Ethnic Disparities in Post-acute Sequelae of SARS-CoV-2 Infection in New York: an EHR-Based Cohort Study from the RECOVER Program. J Gen Intern Med [Internet]. 2023 Apr 1 [cited 2024 Feb 10];38(5):1127–36. Available from: https://link.springer.com/article/10.1007/s11606-022-07997-1

67. McMaughan DJ, Oloruntoba O, Smith ML. Socioeconomic Status and Access to Healthcare: Interrelated Drivers for Healthy Aging. Front Public Health [Internet]. 2020 Jun 18 [cited 2024 Feb 10];8:231. Available from: /pmc/articles/PMC7314918/

For peer review only

68. Tripepi G, Jager KJ, Dekker FW, Zoccali C. Selection Bias and Information Bias in Clinical Research. Nephron Clin Pract [Internet]. 2010 Jun 1 [cited 2024 Feb 10];115(2):c94–9. Available from: https://dx.doi.org/10.1159/000312871

69. NHS England. Statistics » COVID-19 Post-Covid Assessment Service [Internet]. 2024 [cited 2024 Feb 17]. Available from: https://www.england.nhs.uk/statistics/statistical-work-areas/covid-19-post-covid-assessment-service/

For peer review only

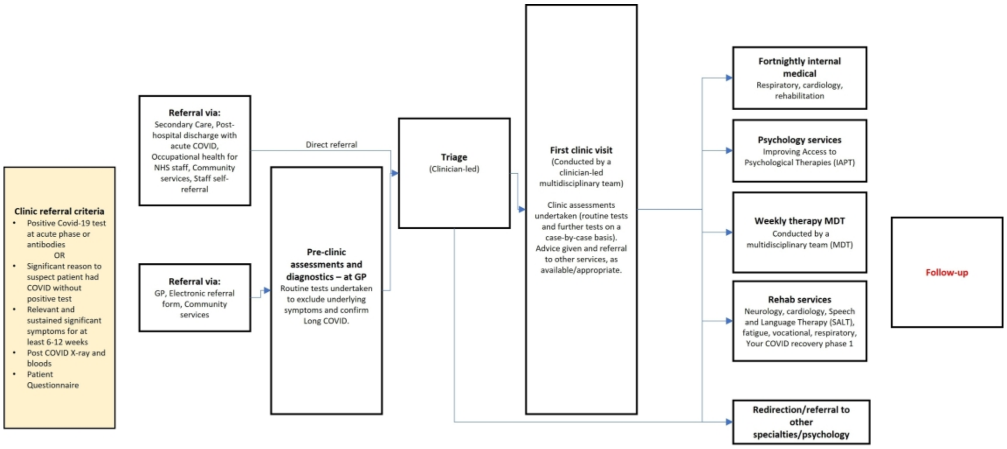

Figure 1. Long COVID Patient Pathway

298x132mm (300 x 300 DPI)

STIMULATE-ICP - WP1

Interview Schedule – Patients

Introduction

1. Review of Participant Information Sheet
2. Consent form
3. Timing and confidentiality
4. My name is
5. I am a researcher, I'm not a doctor or a psychologist and I don't have any clinical contact with your team.
6. I'm interested in finding out from you what your experiences have been with your care for Long-COVID
7. Overview of sections: your experiences, care you have received, your experiences of the service
8. There are no right answers
9. Appreciate symptoms include fatigue and brain fog, so let me know if you need to take a break etc.

**10. Precipitating events**	**Take me back, can you tell me about when you were first unwell with COVID-19 up to when you felt that you had Long Covid?**
Prompts	• Can you remember feeling unwell?<br>• Was there anything else you think may have contributed to how you were feeling? *What else going on at that time?*<br>• How do you think that experience relates to how you feel today?<br>• **Can you talk me through the support you received during this time?**<br>○ E.g. family, friends, GP?<br>○ How did you find out you had COVID-19? E.g. Who told you? When?<br>○ How did you feel when you found out it was COVID-19?<br>○ COVID-19 and other chronic conditions. Exacerbates other chronic conditions and affects management of it?<br>• When did you realise your symptoms weren't going away?<br>• **How did you find out about the post-COVID/Long Covid clinic**?
**11. Symptoms**	**Can you tell me about a time when you felt your Long-COVID was at its worst?**
Prompts	• How did it feel?<br>• How long did it last?<br>• Can you tell me how most days are for you at the moment?<br>• What's a good day for you?<br>• How long have you been feeling this way?<br>• Are there times when you don't feel this way? How long does this last?<br>• How has this impacted on your work and personal life

Patient Flow diagram

I would like to move on to think about the care you have received for your long-COVID. I'd like you to look at what some other people have experienced on their care journey at [name of clinic].

**Diagram discussion**	**When was it confirmed you had long covid? GP or after referral into clinic?**<br><br>**Has this been your experience of care?**

For peer review only - http://bmjopen.bmj.com/site/about/guidelines.xhtml

	**Do you recognise all the parts to this care diagram?**<br>**Are any parts of your experience missing?**<br>**What part of this care pathway do you think has been the most important to your experience?**

**12. Management of Long COVID**	**What treatment are you receiving for your long-COVID at the moment?**
Prompts	• Can you tell me about the care you have received/are receiving for your Long-COVID from doctors?<br>*e.g. who (GP, clinic nurse/doctor/ physiotherapist/ occupational therapist)*<br>*e.g. where (GP clinic, hospital clinic, community/third sector/private doctor)*<br>*e.g. when (routine/booked appointments, length of appointments)*<br>*e.g. what (medications/treatment/lifestyle advice/social prescriptions/physiotherapy/ vocational rehab/ psychology)*<br>• How did you find the treatment?<br>• How helpful has this treatment been? E.g. what was most helpful and least helpful?<br>• Diagnosis/attending appointments when you're not feeling well? *Better to go and show what you're like at your worst or not be able to make it at all.*
**13. Service evaluation**	• **Can you talk me through anything you would change about the long-COVID care you have received?**
Prompts	• How long did you have to wait to be seen by your GP/ by the PCAS service/ by onward rehabilitation services?<br>• Have any parts of your care been particularly helpful or unhelpful?<br>• Have you received the care you expected to when you were referred?<br>• Did you feel listened to and understood by your GP/ clinic doctors/ any therapist you have met<br>• What would you like to happen in the future?<br>• Were there any specific types of doctor you would have liked to have seen?<br>• Would you have like to have seen a psychologist<br>• Did you receive support form a physiotherapist or occupational therapist?<br>• Would you like to go back to the clinic in the future?<br>• Would you recommend the PCAS clinic / rehabilitation service to other patients<br>• **Where do you go to for information about long covid?**<br>• Do you feel like HCPs are knowledgeable / open to new evidence about LC?

Wrapping up

14. Is there anything else you would like to tell me?
15. How have you found this interview?
16. Do you have any questions?

For peer review only - http://bmjopen.bmj.com/site/about/guidelines.xhtml

17. Thank you for your time.
18. Give sources of further support handout.
19. Give baseline questionnaire.

STOP RECORDING

<u>Baseline questions to complete at end of interview.</u>

If they have already answered this during the interview, can move on to the next question, or confirm.

Script:

Thank you for participating in this interview. We need to collect some background information about you, so that we can describe which groups of people participated in our study. This will be stored separately from the transcript of this conversation and will not include identifying information, such as your name or date of birth. If there is any question you would prefer not to answer we can move on.

- What is your age?
- What is your gender?
- How would you describe your ethnic group?
- What languages do you speak?
- What is your highest level of educational qualification?
- What is the nature of your employment? (If you're no longer employed due to LC symptoms, what was your previous job)
- Aside from your Long Covid symptoms, do you have any longstanding illnesses, diseases or medical conditions? These can include conditions relating to your physical and mental health (e.g. asthma, depression, diabetes, previous heart attack, osteoporosis).

<u>Information for interviewers</u>

Do not recommend that patients should complain. However if they ask where to complain or raise concerns, you can direct them to their local PALS. Information for that is in the participant information sheet, or can offer to resend the details.

If patient is very distressed, you can recommend that they get in touch with their GP or post-covid service. Similarly, you can remind them that they can let the GP know if anything has changed. You can let them know that the post-covid service has offered that we can help them connect with them, if they would like to see them.

If you have an immediate concern that there is a risk of harm to the patient or to others, e.g. they express suicidal thoughts without prompting, you have a duty to report this. In this instance, you should phone the site clinical lead. You can inform the patient that it is your responsibility to report this, and go ahead without their consent. However you should only do this if you have a compelling reason to do so; otherwise do not breach confidentiality, suggest that the patient follows up with the GP or post-covid service.

For peer review only - http://bmjopen.bmj.com/site/about/guidelines.xhtml

Interview guide – Interview with HCP

n=24, up to four health professional categories will be nominated at each site, and recruitment will take place within those professionals. Max four interviews per site across the six sites.

The questions below are a guide but will be adapted based on HCP type and service delivery model.

**1. Introduction**	• Introductions<br>• Purpose of discussion is to understand experience of HCPs within each site to enable us to understand how usual care is being delivered.
**2. Individual background**	**Can you tell me about your clinical background?**
Prompts	• Background, speciality, grade, years since qualification
**3. Employment in current service**	**How did you come to work in the Post COVID service?**
Prompts	• E.g. applied, seconded (where did you come from?)<br>• How long is your contract or secondment?<br>• Do you work from home or on site?<br>• Did you feel that you had previous skills or experience that was relevant to this setting?<br>• Do you want to continue working in this field in the long term?<br>• Have you experienced any long COVID symptoms yourself?
**4. Training**	**Can you tell us about the induction, training and support you have received in this role?**
	• Were you offered any training when you entered the role? [*relative to what you would expect moving into another new department, specific to LC*]<br>• Are there resources available, and have you used them?<br>• For example, have you accessed the NHS Futures site for Long COVID, attended webinars?<br>• Do you feel able to keep up to date with developments?<br>• What additional training in Long-COVID care would you like? (How would you like it to be delivered)
**5. HCP experience**	**What are the main opportunities or challenges of your role?**
Prompts	• Do you feel you have adequate time in your job plan to do this work?<br>• Managing patient disappointments (waiting list, no clear treatment)

For peer review only - http://bmjopen.bmj.com/site/about/guidelines.xhtml

Workforce experience –Interview Guide

	• Keeping up with patient research/knowledge / responding to it / non-validated interventions and home remedies / patient safety "*This is a rapidly moving area, with patients doing a lot of their own research. What are your concerns and/or strategies for handling this?*"
**6. Patient presentation**	**What is the typical presentation of patients for the services you deliver?**
Prompts	• What symptoms do patients present with most frequently?<br>• What underlying comorbidities are typically seen?<br>• How has this changed over the course of the pandemic? (e.g. different waves, variants, vaccination, etc.)?<br>• *[pick up differences from service lead answers]*
**7. Patient experience of care**	**How would you describe a typical patient pathway in this service?**
Prompts	• **Introduce map.**<br>• What is the typical experience of referral, experience in clinic, and onward referral.<br>• What are factors that lead to a different experience?<br>• What would you describe as a good outcome or a poor outcome for a patient on the pathway? [audit, challenge]<br>• How would you describe patient access to rehab or psychology support?<br>• NHS Staff?<br>• Community vs previously hospitalised Long covid patients? Has this changed?
**8. Quality of care**	**Where do you feel the service delivers good care for patients?**
Prompts	• Have you had feedback from patients on what works well, or what they would like to improve?<br>• What do you feel are the barriers to delivering good care (if any)? E.g. high vacancy rate, high demand/low capacity?<br>• Can you identify any barriers to access to care?<br>• What are the opportunities to improve patient experience of care?<br>• How do you think patient outcomes might be improved?<br><br>*What do we mean? Quality vs effectiveness.*
**9. Inequalities**	**In your experience, has there been equitable uptake of the service across different groups in society e.g. those from socially disadvantaged backgrounds?**
Prompts	- Who is referred vs who turns up? How do you manage DNAs?<br>- Who is accessing the clinic?

For peer review only - http://bmjopen.bmj.com/site/about/guidelines.xhtml

Workforce experience –Interview Guide

	- Why do you think this may be?<br>- Do you think those most at need are accessing the service?<br>- Are there particular socio-demographic groups who struggle to access the clinic?<br>- What do you think may be causing the equal/unequal uptake?<br>- Is the clinic doing anything to try and actively access those most in need?
**10.**	**What kind of immediate improvements do you want to see in the service?**
	- Funding<br>- Staffing<br>- Evidence for change might lead to overloaded capacity
11. **Wrapping up**	**Opportunity to ask questions or add in further detail**

Stop recording

Baseline questions to complete at end of interview.

If they have already answered this during the interview, can move on to the next question, or just confirm details.

Script:

Thank you for participating in this interview. We need to collect some background information about you, so that we can describe which groups of people participated in our study. This will be stored separately from the transcript of this conversation and will not include identifying information, such as your name or date of birth. If there is any question you would prefer not to answer we can move on.

- What is your age?
- What is your gender?
- How would you describe your ethnic group?
- Can you provide the following details about your role:
    - Background -
    - Speciality –
    - Grade –
    - Years since qualification
- Have you had Long Covid yourself?

For peer review only - http://bmjopen.bmj.com/site/about/guidelines.xhtml

Standards for Reporting Qualitative Research (SRQR)*

http://www.equator-network.org/reporting-guidelines/srqr/

Page/line no(s).

Title and abstract

**Title** - Concise description of the nature and topic of the study Identifying the study as qualitative or indicating the approach (e.g., ethnography, grounded theory) or data collection methods (e.g., interview, focus group) is recommended	1
**Abstract** - Summary of key elements of the study using the abstract format of the intended publication; typically includes background, purpose, methods, results, and conclusions	1

Introduction

**Problem formulation** - Description and significance of the problem/phenomenon studied; review of relevant theory and empirical work; problem statement	2
**Purpose or research question** - Purpose of the study and specific objectives or questions	2

Methods

**Qualitative approach and research paradigm** - Qualitative approach (e.g., ethnography, grounded theory, case study, phenomenology, narrative research) and guiding theory if appropriate; identifying the research paradigm (e.g., postpositivist, constructivist/ interpretivist) is also recommended; rationale**	2-3
**Researcher characteristics and reflexivity** - Researchers' characteristics that may influence the research, including personal attributes, qualifications/experience, relationship with participants, assumptions, and/or presuppositions; potential or actual interaction between researchers' characteristics and the research questions, approach, methods, results, and/or transferability	3
**Context** - Setting/site and salient contextual factors; rationale**	2-3
**Sampling strategy** - How and why research participants, documents, or events were selected; criteria for deciding when no further sampling was necessary (e.g., sampling saturation); rationale**	2-3
**Ethical issues pertaining to human subjects** - Documentation of approval by an appropriate ethics review board and participant consent, or explanation for lack thereof; other confidentiality and data security issues	19/20 (Ethics Approval)
**Data collection methods** - Types of data collected; details of data collection procedures including (as appropriate) start and stop dates of data collection and analysis, iterative process, triangulation of sources/methods, and modification of procedures in response to evolving study findings; rationale**	3 (Methods)

For peer review only

For peer review only - http://bmjopen.bmj.com/site/about/guidelines.xhtml

**Data collection instruments and technologies** - Description of instruments (e.g., interview guides, questionnaires) and devices (e.g., audio recorders) used for data collection; if/how the instrument(s) changed over the course of the study	4 (Data Analysis)
**Units of study** - Number and relevant characteristics of participants, documents, or events included in the study; level of participation (could be reported in results)	3 (Methods); 7(Table 1); 9 (Table 2)
**Data processing** - Methods for processing data prior to and during analysis, including transcription, data entry, data management and security, verification of data integrity, data coding, and anonymization/de-identification of excerpts	3-5 (Methods, Data Analysis)
**Data analysis** - Process by which inferences, themes, etc., were identified and developed, including the researchers involved in data analysis; usually references a specific paradigm or approach; rationale**	4 (Data Analysis)
**Techniques to enhance trustworthiness** - Techniques to enhance trustworthiness and credibility of data analysis (e.g., member checking, audit trail, triangulation); rationale**	4 (Data Analysis)

Results/findings

**Synthesis and interpretation** - Main findings (e.g., interpretations, inferences, and themes); might include development of a theory or model, or integration with prior research or theory	5-15
**Links to empirical data** - Evidence (e.g., quotes, field notes, text excerpts, photographs) to substantiate analytic findings	5-15

Discussion

**Integration with prior work, implications, transferability, and contribution(s) to the field -** Short summary of main findings; explanation of how findings and conclusions connect to, support, elaborate on, or challenge conclusions of earlier scholarship; discussion of scope of application/generalizability; identification of unique contribution(s) to scholarship in a discipline or field	15-19
**Limitations** - Trustworthiness and limitations of findings	19

Other

**Conflicts of interest** - Potential sources of influence or perceived influence on study conduct and conclusions; how these were managed	20
**Funding** - Sources of funding and other support; role of funders in data collection, interpretation, and reporting	20

*The authors created the SRQR by searching the literature to identify guidelines, reporting standards, and critical appraisal criteria for qualitative research; reviewing the reference lists of retrieved sources; and contacting experts to gain feedback. The SRQR aims to improve the transparency of all aspects of qualitative research by providing clear standards for reporting qualitative research.

For peer review only

For peer review only - http://bmjopen.bmj.com/site/about/guidelines.xhtml

**The rationale should briefly discuss the justification for choosing that theory, approach, method, or technique rather than other options available, the assumptions and limitations implicit in those choices, and how those choices influence study conclusions and transferability. As appropriate, the rationale for several items might be discussed together.

Reference:

O'Brien BC, Harris IB, Beckman TJ, Reed DA, Cook DA. **Standards for reporting qualitative research: a synthesis of recommendations.** *Academic Medicine*, Vol. 89, No. 9 / Sept 2014 DOI: 10.1097/ACM.0000000000000388

For peer review only

For peer review only - http://bmjopen.bmj.com/site/about/guidelines.xhtml

What is current care for people with Long COVID in England? A qualitative interview study.

Journal:	*BMJ Open*
Manuscript ID	bmjopen-2023-080967.R2
Article Type:	Original research
Date Submitted by the Author:	29-Mar-2024
Complete List of Authors:	Sunkersing, David; University College London, Institute of Health Informatics<br>Ramasawmy, Mel ; University College London, Institute of Health Informatics<br>Alwan, Nisreen; University of Southampton, Academic unit of Primary Care and Population Sciences, Faculty of Medicine; University of Southampton and University Hospital Southampton NHS Foundation Trust, NIHR Southampton Biomedical Research Centre<br>Clutterbuck, Donna ; University of Southampton, Academic unit of Primary Care and Population Sciences, Faculty of Medicine<br>Mu, Yi ; University College London, Institute of Health Informatics<br>Horstmanshof, Kim; University College London<br>Banerjee, Amitava; University College London, Institute of Health Informatics<br>Heightman, Melissa; University College London Hospitals NHS Foundation Trust
**Primary Subject Heading**:	Health services research
Secondary Subject Heading:	Qualitative research
Keywords:	COVID-19, QUALITATIVE RESEARCH, PUBLIC HEALTH, Post-Acute COVID-19 Syndrome, Health Services

SCHOLARONE™
Manuscripts

I, the Submitting Author has the right to grant and does grant on behalf of all authors of the Work (as defined in the below author licence), an exclusive licence and/or a non-exclusive licence for contributions from authors who are: i) UK Crown employees; ii) where BMJ has agreed a CC-BY licence shall apply, and/or iii) in accordance with the terms applicable for US Federal Government officers or employees acting as part of their official duties; on a worldwide, perpetual, irrevocable, royalty-free basis to BMJ Publishing Group Ltd ("BMJ") its licensees and where the relevant Journal is co-owned by BMJ to the co-owners of the Journal, to publish the Work in this journal and any other BMJ products and to exploit all rights, as set out in our licence.

The Submitting Author accepts and understands that any supply made under these terms is made by BMJ to the Submitting Author unless you are acting as an employee on behalf of your employer or a postgraduate student of an affiliated institution which is paying any applicable article publishing charge ("APC") for Open Access articles. Where the Submitting Author wishes to make the Work available on an Open Access basis (and intends to pay the relevant APC), the terms of reuse of such Open Access shall be governed by a Creative Commons licence – details of these licences and which Creative Commons licence will apply to this Work are set out in our licence referred to above.

Other than as permitted in any relevant BMJ Author's Self Archiving Policies, I confirm this Work has not been accepted for publication elsewhere, is not being considered for publication elsewhere and does not duplicate material already published. I confirm all authors consent to publication of this Work and authorise the granting of this licence.

For peer review only

Title: What is current care for people with Long COVID in England? A qualitative interview study.

Authors: David Sunkersing[1], Mel Ramasawmy[1], Nisreen A Alwan[2], Donna Clutterbuck[2], Yi Mu[1], Kim Horstmanshof[1], Amitava Banerjee[1] and Melissa Heightman[3].

Corresponding Author: David Sunkersing (University College London, Institute of Health Informatics, London, UK; david.sunkersing@ucl.ac.uk; +44 20 8016 8424)

Author Institutions:
1. University College London, Institute of Health Informatics, London, UK
2. University of Southampton, Academic unit of Primary Care and Population Sciences, Faculty of Medicine, Southampton, Hampshire, UK
3. University College London Hospitals NHS Foundation Trust, London, London, UK

Keywords: COVID-19, QUALITATIVE RESEARCH, PUBLIC HEALTH, Post-Acute COVID-19 Syndrome, Health Services.

Word Count (Excluding Title Page, References, Figures): 4934

For peer review only

Abstract

Objective: To investigate current care for people with Long COVID in England.

Design: In-depth, semi-structured interviews with people living with Long COVID and Long COVID healthcare professionals; data analysed using thematic analysis.

Setting: NHS England (NHSE) post-COVID services in six clinics from November 2022 to July 2023.

Participants: 15 healthcare professionals and 21 people living with Long COVID currently attending or discharged (18 female; 3 male).

Results: Health professionals and people with lived experience highlighted the multifaceted nature of Long COVID, including its varied symptoms, impact on peoples' lives and the complexity involved in managing this condition. These impacts encompass physical, social, mental and environmental dimensions. People with Long COVID reported barriers in accessing primary care, as well as negative GP consultations where they felt unheard or invalidated, though some positive interactions were also noted. Peer support or support systems proved highly valuable and beneficial for individuals, aiding their recovery and well-being. Post-COVID services were viewed as spaces where overlooked voices found validation, offering more than medical expertise. Despite initial challenges, healthcare providers' increasing expertise in diagnosing and treating Long COVID has helped refine care approaches for this condition.

Conclusion: Long COVID care in England is not uniform across all locations. Effective communication, specialised expertise and comprehensive support systems are crucial. A patient-centred approach considering the unique complexities of Long COVID, including physical, mental health, social and environmental aspects is needed. Sustained access to post-COVID services is imperative, with success dependent on offering continuous rehabilitation beyond rapid recovery, acknowledging the condition's enduring impacts and complexities.

Data availability statement

Data are available on reasonable request. Anonymised data can be made available on request.

STRENGTHS AND LIMITATIONS OF THIS STUDY

- This study used in-depth, semi-structured interviews to provide a rich account of current care, from participants across NHSE commissioned post-COVID services in England.
- Voluntary participation from individuals able to access a post-COVID service could lead to an under-representation of certain respondent types.
- As this study aimed to investigate perspectives from 6 post-COVID sites, the findings may not be generalisable or representative of other post-COVID sites in England.

Introduction

Long COVID (or "Post-Acute Sequelae of SARS-CoV-2 infection" (PASC))(1,2) refers to a complex and poorly understood set of persistent symptoms that can continue for weeks, months or longer after an initial COVID-19 infection. First identified online from individuals with

For peer review only

ongoing symptoms of COVID-19, it quickly gained recognition in formal clinical channels within months.(3) Estimates suggest at least 65 million people worldwide have Long COVID, with reported symptoms including extreme fatigue, breathlessness, cognitive impairments and depression.(1,4,5) For some, Long COVID leads to prolonged recovery, severely impacting their work, daily activities and social relationships.(6) Importantly, the recognition and understanding of Long COVID have been enhanced by the crucial role played by people with the condition themselves, including through supportive peer-to-peer interactions, where valuable networks of shared experiences and coping strategies have been exchanged.(7) However, as symptoms often present heterogeneously, diagnosis and treatment are challenging.(1,4,8)

In September 2020, recognising the distinctive and persistent healthcare needs of individuals with Long COVID,(3,9) the Royal College of General Practitioners (RCGP) called for the establishment of a national network of 'post-COVID' services. In response, NHS England (NHSE) committed to rapidly expanding new and strengthened rehabilitation centres.(10) This occurred when only a minority of Clinical Commissioning Groups (CCGs) had successfully implemented post-COVID services, reflecting challenges in adapting healthcare services for people with Long COVID. In October 2020, NHSE implemented a comprehensive five-part package to bolster support for people with Long COVID.(11) This included the release of new guidance on managing Long COVID, the introduction of an online rehab service ('Your Covid Recovery'), designated Long COVID clinics/post-COVID services (to treat Long COVID symptoms, deliver holistic assessments with a multidisciplinary team, offer medical support, diagnostics and referral and rehabilitation where needed),(12) NIHR-funded research and establishing a new NHSE Long COVID task force.(12,13) These initiatives underscored the evolving understanding of Long COVID and the imperative for healthcare services to adapt rapidly to effectively support those navigating the complex and prolonged effects of the virus.

For people with suspected Long COVID, UK guidance advises beginning with a GP consultation for an initial assessment.(14) This involves discussing symptoms, ruling out underlying conditions and evaluating their impact on daily life. Referral to specialised services like rehabilitation or post-COVID services may follow if symptoms severely disrupt daily functioning, in accordance with NICE and WHO recommendations.(15,16) Despite this guidance, care for people with Long COVID can vary – and people have reported difficulties in accessing care,(17,18) as well as varied experiences in their treatment and support.(19) For healthcare professionals, the existing complexity in addressing Long COVID care is compounded by ongoing staff shortages, under-resourcing and insufficient funding.(20–22)

Currently, there is lack of in-depth, critical analyses concerning patient experiences on their ongoing care for Long COVID.(17,23,24) Similarly, the challenges of implementing effective post-COVID services contributes to the limited evidence on healthcare professionals' perspectives in experiences of managing Long COVID care.(18,25) To effectively manage and improve care for people living with Long COVID, it is crucial to evaluate current healthcare approaches for this specific patient group and their healthcare professionals. Our study aimed to address these gaps by conducting an in-depth investigation into current care within a sample of newly established post-COVID services in England. This paper presents findings from semi-structured interviews conducted with both individuals experiencing Long COVID and healthcare professionals providing Long COVID care.

For peer review only - http://bmjopen.bmj.com/site/about/guidelines.xhtml

Methods

Study Design and Setting

People living with Long COVID, either discharged or under follow up from six sites across England were invited to participate virtually over Microsoft Teams in either semi-structured interviews or focus groups (from November 2022 to July 2023). Healthcare professionals providing Long COVID care at each of the six sites were also invited to participate.

In addition to the above, the study inclusion criteria were >18 years old, English speaker and competent to provide written consent.

Recruitment of people with Long COVID

The service manager and clinical lead for each of the six post-COVID services, which were identified as part of a previous study, known as STIMULATE-ICP (Symptoms, Trajectory, Inequalities and Management: Understanding Long-COVID to Address and Transform Existing Integrated Care Pathways),(26) were asked to review their database of people living with Long COVID, including those that may have signed up to take part in research. Depending on the most appropriate site-specific means to approach people living with Long COVID we contacted (via email, via an online Long COVID group at one site (led by the post-COVID service) and/or via an internal hospital communication app at another site (MyCare application)) them to advertise the project and request their involvement. Voluntary response sampling (i.e. where participants had volunteered themselves to participate) was employed in this study to accommodate the unique challenges and vulnerabilities of people with Long COVID – and, importantly, recognising that those affected may experience limitations in time and energy. Although this sampling strategy, has limitations, we felt that this sampling strategy offered a more feasible and considerate approach for gathering valuable insights into the experiences of people with Long COVID.

Regular contact with all six post-COVID services was made throughout the data collection period to maximise participation. The principle of saturation guided the sample size, and semi-structured interviews were conducted until additional participants did not yield new information.

Recruitment of Healthcare Professionals

Apart from one site, each service manager and clinical lead additionally identified Long COVID healthcare professionals who were willing to participate in the study. We chose this approach – voluntary response sampling – as we considered it the most pragmatic and efficient method for collecting data.

In our healthcare professional interviews, we also investigated the route of individuals with Long COVID through a site's post-COVID services to understand the structure, delivery and accessibility of care of the Long COVID pathway.

Data Collection and Semi-Structured Interview

Each participant was asked to describe their experience of care, including referral, assessment, diagnosis and treatment (see supplementary materials 1 and 2 for full interview guides). For people living with Long COVID, the aim of the semi-structured interviews/focus groups was to gain a greater understanding of their perception of care including any barriers to accessing

For peer review only

care, their experience of the care pathway and any outcomes they experienced due to treatment. For healthcare professionals providing care for people living with Long COVID, we wanted to understand the delivery of Long COVID care from their perspective, including any challenges, historical or current. Semi-structured interviews/focus groups were selected as the data collection approach because they can provide comprehensive, profound, and detailed narratives of participants' experiences, viewpoints, and thoughts. They also strike a balance between flexibility and structure, allowing for an in-depth exploration of individual experiences with Long COVID while providing a framework for key topics. This approach enabled us to adapt the interview based on participants' responses, fostering a richer understanding of their perspectives and capturing nuanced information that may not have emerged in more rigidly structured interview formats. The semi-structured interviews were conducted by DS and MR via Microsoft Teams, who are both experienced qualitative researchers. Semi-structured interviews were conducted with both people with Long COVID and healthcare professionals until saturation was achieved and no new information was forthcoming.

Data Analysis

All participants in this study opted to take part in a semi-structured interview – i.e., no focus groups – between November 2022 to July 2023. All semi-structured interviews were (with participant consent) audio recorded and automatically transcribed verbatim in Microsoft Teams using the 'transcribe feature', which converts speech to a text transcript with each speaker individually separated.

An inductive, data-driven approach(27) to data analysis was undertaken by DS and MR. Firstly, each transcript was reviewed for accuracy and initial codes were generated using support from the qualitative analysis software programs ATLAS.ti (version 5.8.0)(28) and NVivo V.12.(29)

Following initial code generation, these were reviewed and further refined. The process was concluded when an agreement that the overarching themes effectively encapsulated the narratives present in the data for both people with Long COVID and Healthcare Professionals.

Three overarching themes were generated ((1) : Understanding Long COVID: Symptoms and Lived Realities, (2) Long COVID Care: Non-Specialist Service Experiences (3) Long COVID Care: Post-COVID Service Experiences) and a number of supporting, subsidiary subthemes.

Two researchers (DS and MR) met regularly to discuss the transcripts and discuss whether the overarching themes were reflective of the presented data. These findings were also discussed with AB (and patient representatives) for review. After review, there was agreement that the themes reflected the transcripts appropriately.

This approach of generating shared overarching themes for both people with Long COVID and healthcare professional perspectives was done to enable a deeper understanding of shared and divergent perspectives – an approach used in other studies.(30,31)

Patient and Public Involvement (PPI)

A PPI group (n=11) features in our regular management and oversight meetings. This group comprises individuals who either have Long COVID or provide care for someone with Long COVID. PPI members have contributed to the research components in this study, including informing the semi-structured interview design and approach. We also discussed the findings

For peer review only

of this study with our PPI to facilitate our interpretations of the data. The PPI group will continue to be involved in the dissemination of findings.

Results

A total of 21 people with Long COVID were interviewed from four of the six possible sites (10 from service a, 8 from service b, 2 from service c, 0 from service d, 1 from service e and 0 from service f). For people with Long COVID, the nature of their symptoms, privacy concerns, emotional challenges and/or ongoing medical commitments may have impacted participation.

All six post-COVID services were approached and contacted, with five services able to identify healthcare professionals willing to participate in the study. A total of 15 healthcare professionals were interviewed (5 from service a, 4 from service b, 3 from service c, 0 from service d, 1 from service e and 2 from service f).

We believe the smaller than anticipated sample size in this study can be attributed to staff shortages, existing pressures within the NHS and time availability. At one specific service, the absence of semi-structured interviews with healthcare professionals can be attributed to unforeseen circumstances, which hindered the engagement and participation of relevant personnel in the study.

Characteristics of people living with Long COVID are detailed in Table 1; Characteristics of healthcare professionals providing Long COVID care are detailed in Table 2.

We found that a generic Long COVID pathway was followed by many sites (Figure 1), although comprehensiveness of testing and/or referral ability varied, based on available staffing, other resources and local infrastructure.

[Figure 1. Long COVID Patient Pathway]

From our healthcare professional interviews, we were able to establish the service model used at each post-COVID service:

a: Face-to-Face

b: Hybrid (predominantly virtual; some aspects face-to-face)

c: Face-to-Face

d: Hybrid (dependent on patient)

e: Hybrid (dependent on patient)

f: Hybrid (dependent on patient – home visits, if necessary)

Information from post-COVID service “d” was obtained from publicly available data, as no direct interview was conducted with this site.

For peer review only

Table 1: Characteristics Of People Living With Long COVID.

Characteristic		Frequency	Percentage (%)
**Sex**	Male	3	14
	Female	18	86
**Age**	18-27	0	0
	28-37	1	5
	38-47	12	57
	48-57	6	29
	58-67	0	0
	68-77	2	10
**Ethnic Group**	White-British	12	57
	White - Any other White background	3	14
	Asian or Asian British - Indian	3	14
	Other Ethnic Groups - Any other ethnic group	2	10
	Not stated	1	5
**Languages Spoken**	English (Only)	14	67
	Multiple (Including English)	7	33
**Highest Educational Qualification**	GCSE/Equivalent	1	5
	A-Level	1	5
	Apprenticeship	1	5
	Undergraduate Degree	8	38
	Master's Degree	8	38
	PhD	1	5
	Not Stated	1	5
**Profession (Current or Prior to Covid/Long COVID (LC))**	Creative arts or design	2	10
	Education	1	5
	Engineering	1	5
	Financial	2	10
	Healthcare (Clinical Facing Role)	9	43
	Marketing, Advertising or PR	1	5

For peer review only

	Public Services or Administration	2	10
	Unemployed	1	5
	Retired	1	5
	Not Stated	1	5
**Comorbidities**	Yes	16	76
	No	4	19
	Not Stated	1	5
**LC Onset**	January - April 2020	6	29
	May - August 2020	2	10
	September - December 2020	2	10
	January - April 2021	1	5
	May - August 2021	4	19
	September - December 2021	0	0
	January - April 2022	5	24
	May - August 2022	1	5
	September - December 2022	0	0
**Post-COVID Service Attendance**	January - April 2020	0	0
	May - August 2020	2	10
	September - December 2020	3	14
	January - April 2021	3	14
	May - August 2021	0	0
	September - December 2021	2	10
	January - April 2022	1	5
	May - August 2022	4	19
	September - December 2022	5	24
	January - April 2023	1	5

For peer review only

Table 2: Characteristics of Healthcare Professionals Providing Long COVID Care.

Characteristic		Frequency	Percentage (%)
**Sex**	Male	3	20
	Female	12	80
**Age**	18-27	1	7
	28-37	5	33
	38-47	3	20
	48-57	2	13
	58-67	0	0
	68-77	0	0
	Not stated	4	27
**Ethnic Group**	White-British	6	40
	White-Any other White background	2	13
	Asian or Asian British - Indian	2	13
	Other Ethnic Groups - Any other ethnic group	2	13
	Not stated	3	20
**Profession**	Clinical Psychologist	1	7
	Clinical Trials Manager	1	7
	Doctor (Gastro)	1	7
	Exercise Physiologist	2	13
	Lead Therapist (Physio)	1	7
	Nurse	1	7
	Nurse (Service Lead)	1	7
	Occupational Health Physician & Post COVID Service (Medical Lead)	1	7

For peer review only

	Physiotherapist	1	7
	Post Covid Lead	1	7
	Rehabilitation Medicine Consultant	1	7
	Respiratory Physiotherapist	1	7
	Senior Specialist Physiotherapist	1	7
	Team Lead Post Covid Service	1	7
**Have they had Long COVID?**	Yes	3	20
	No	8	53
	Not Stated	4	27

To maintain participant anonymity, it is not possible to give further details on their roles.

For peer review only

Three overarching themes were identified and are elaborated further with example supporting quotations in boxes 1 – 3. We have refrained from attributing quotes to specific post-COVID services to avoid potential identification of specific services and participants.

Theme 1: "Understanding Long COVID: Symptoms and Lived Realities".

Within this theme, people living with Long COVID reported on several factors relating to their Long COVID experiences. The ‘physical’ subtheme underscores the persistent battles with fluctuating symptoms, highlighting the complexities that impact daily life (quotes 1 – 2, box 1). The ‘mental health’ subtheme captures the emotional toll of uncertainty, anxiety and stress related to the nature of the condition (quotes 3 – 4, box 1). The ‘social’ subtheme explores ‘loss of identity, or self’, as people living with Long COVID grapple with both understanding and support (quote 5, box 1). Lastly, the theme pays heed to the often-overlooked environmental factors, examining how the interplay between personal surroundings and the broader context can shape the Long COVID journey (quote 6, box 1). The interviews additionally established an evident impact of Long COVID on their working environment – and, for some, a lack of universal appreciation and/or adjustment from colleagues (quotes 7 – 8, box 1).

Overall, these subthemes weave a narrative that underscores the complex challenges faced by people living with Long COVID.

Box 1

Subtheme	Example Supporting Quote
Physical	1. *“I've got symptoms of that I've never experienced in a life before, you know, the aching joints, the headaches.”. [Participant T, Male, Age Range 68-77]*
Physical	2. *“…the first few days, it was a lot of kind of like coughing, breathlessness, fevers, et cetera. And then kind of over the first few days starting to get kind of extreme tiredness and fatigue….”. [Participant S, Female, Age Range 28-37]*
Mental Health	3. *“…there's a lot going on from a kind of stress, emotional sort of perspective.”. [Participant J, Female, Age Range 38-47]*
Mental Health	4. *“…the most thing from [Long] Covid that affected us is our mental health and the lack of sleep.” [Participant H, Female, Age Range 48-57*
Social	5. *“You know I’m shadow of the person I was and it's very hard to to accept that…”. [Participant C, Female, Age Range 38-47].*
Environmental	6. *“So instead of going into a cafe, for example, we'll get a coffee and sit in the park because there's less risk. So I'm still trying to maintain a life, but it's completely different.”. [Participant N, Female, Age Range 38-47]*
Working Environment	7. *“…and actually working online, I think is one of the really toughest things if you've got Long COVID.”. [Participant A, Female, Age Range 48-57]*
Working Environment	8. *“You know they don't even give me time off for my appointments… sometimes I have to do them at work if I like. Take phone calls at work. You know it's really difficult.”. [Participant E, Female, Age Range 38-47].*
Multi-dimensional	9. *“Most of the patients come with cluster of symptoms, majority fatigue, breathlessness and also muscle aches, joint pains and the brain fog is also very common. The same cognitive issues forgetfulness and some with cardiac.”. [Nurse (Service Lead)].*

For peer review only

Multi-dimensional	10. *"…and physical symptoms with long COVID patients and they are often burdened with quite a lot of mental health issues just because of the nature of the illness.". [Doctor (Gastro)].*

Healthcare professionals, in their semi-structured interviews, echoed and acknowledged the multifaceted nature of Long COVID, aligning with the reports and experiences shared by people living with Long COVID in this study (quotes 9 – 10, box 1).

By addressing physical, mental, social and environmental dimensions, this theme highlights the holistic nature of care needed for people living with Long COVID navigating the complexities of the condition and underscores the importance of a comprehensive and integrated approach to their well-being.

Theme 2: "Long COVID Care: Non-Specialist Service Experiences".

This theme reflects the intricate journey that people living with Long COVID undertake within the healthcare system in non-specialist settings (i.e. outside of a post-COVID service). This theme encompasses a participant's experiences, challenges and interactions within the healthcare system, highlighting their encounters with medical consultations and also support outside of healthcare settings.

Our study found that securing an initial appointment with a GP was challenging, both in terms of contact (quote 1, box 2), but also with respect to waiting times. Several people with Long COVID described lengthy wait times for appointments and a lack of efficient continuity in care (quotes 2 – 3, box 2). Moreover, our study reports encounters from people living with Long COVID where, after securing an appointment, their GP seemed to avoid comprehensive discussions regarding the intricacies of their Long COVID and/or people reported frustrations with the service received (quotes 4 – 5, box 2). Some emphasised a sense of being rushed or their concerns being sidelined, highlighting the significance of effective communication between people living with Long COVID and healthcare professionals (quote 6, box 2). These can all further exacerbate the challenges of Long COVID management.

Despite these comments, however, some people living with Long COVID were keen to make a note of the positive aspects that their GP had brought about (quotes 7 – 8, box 2). However, for many people with Long COVID, they were especially keen to highlight the profound impact of community support networks (quotes 9 – 10, box 2). They emphasised the reassurance and validation found in shared experiences within online communities, as well as the crucial role played by friends and family in offering understanding and solidarity during the challenging journey of coping with long-lasting symptoms.

Lastly, we spoke to people with Long COVID who, though receiving some form of care outside of a post-COVID service, were told that it was limited in addressing all aspects of their Long COVID (quote 11, box 2). While their experiences from these healthcare professionals were positive, the need for dedicated resources and expertise to effectively manage the intricate aspects of Long COVID were still apparent.

Box 2

Subtheme	Example Supporting Quote

For peer review only

Perceived Lack of Support	1. *[Regarding their GP Practice] "It's impossible. You can't contact them in any way by phone or on the portal, and so you send them emails, and then no one, no one replies. They just ignore you". [Participant E, Female Age Range 38-47]*
Waiting Times	2. *"Yeah, I think that's the like having to wait to 12 weeks kind of variability on the GP's". [Participant S, Female, Age Range 28-37]*
Waiting Times	3. *"To see GP it was nightmare. I don't know if it's something to do particularly with my particular surgery...In terms of Long COVID clinic, it was in on average between three and six months.". [Participant F, Female, Age Range 48-57]*
Frustrations With Primary Care Service	4. *"… you get into the into the, into the the GP's office…and then you get 5 minutes. That's all really 5, possibly less than 10, less than 10 anyway. A lot less than 10. And they're under pressure…it's it's not good.". [Participant T, Male, Age Range 68-77]*
Frustrations With Primary Care Service	5. *"We spoke to our practice manager – they didn't even process my husband's kidney scans. They didn't even tell him that he needed it from his CT – that there's something wrong with my husband's kidneys. So you know, we've been treated so so badly. But we can't move GPs again…they're all just as bad as each other, and moving won't help us". [Participant H, Female, Age Range 48-57]*
Negative Clinical Engagements	6. *"…I was very much dismissed by the GP – she was quite unkind, actually. I think she just thought I was a bit nuts. I think she just thought its kind of PTSD, gone a bit nuts and very dismissive…". [Participant J, Female, Age Range 38-47]*
Positive Clinical Engagements	7. *"[I] was under so many people, I couldn't remember what's going on. She [the GP] had a list and she would just say, right, what's happening with this one? What's happening with that? Let's keep a track of everything. And it was really good because someone was keeping an eye out that I wasn't missing out anything or something need chasing.". [Participant E, Female, Age Range 38-47]*
Positive Clinical Engagements	8. *"And they've [regarding their clinicians] been more than amazing like I can't even tell you. They they've they've listened to me. They've cared for me when they were really worried. I had some problems with work and sick leave, and whatever my GP was like, I'm gonna call you in two weeks' time just to see how you are, you know, unnecessarily, really, but just kindness…I can't praise them highly enough.". [Participant E, Female, Age Range 38-47]*
Support Networks	9. *"So the Facebook community…that community was was amazing because you knew for the first time that it wasn't just you and it wasn't. You weren't alone. So that was incredible.". [Participant N, Female, Age Range 38-47]*
Support Networks	10. *"… there's sort of support from friends also with Long COVID or you know, and obviously friends and family members.". [Participant G, Female, Age Range 38-47]*
Clinical Challenges	11. *"The respiratory physios…were, you know, still being very nice and still being very supportive but basically saying, 'look, we we feel we've got to the end of what we can do. We're not Long COVID specialists, we're not funded for for doing Long COVID we think that any residual problems in your breathing patterns are all mixed up with autonomic dysfunction.". [Participant G, Female, Age Range 38-47]*

For peer review only

This theme particularly underscores the importance of fostering an environment where people living with Long COVID can a) contact their healthcare provider and b) openly express their experiences without fear of disbelief. Importantly, the theme sheds light on the complexities and 'patient-provider' dynamics of Long COVID care, emphasising the need for improved 'patient-provider' communication, support and more efficient pathways within the healthcare system. Finally, the significance of a robust support network, where an individual's experiences feel validated cannot be overstated in the context of Long COVID.

Theme 3: "Long COVID Care: Post-COVID Service Experiences".

As noted in Theme 2, participants recount their initial struggles in accessing appropriate care, emphasising the importance and need for specialised post-COVID services. The establishment of dedicated services tailored to people living with Long COVID has also offered a sense of validation and recognition (quotes 1 – 2, box 3). For many people with Long COVID, these services represent more than just medical facilities; they serve as havens where individuals, perhaps previously unheard, find their voices listened to. Beyond medical reassurance, the services provide a space where the legitimacy of their symptoms and experiences are acknowledged, fostering a sense of validation and understanding that transcends merely physical symptoms. However, some people with Long COVID suggested that they expected more comprehensive or hands-on support from these post-COVID services (quote 3, box 3). Nevertheless, these services signify a commitment to understanding the complexities of Long COVID and acknowledging its lasting effects. Overall, people living with Long COVID appear to appreciate this understanding and Long COVID expertise.

Box 3

Subtheme	Example Supporting Quote
Post-COVID Service Experiences	1. *"They [post-COVID service] made me feel heard. But it wasn't until they got involved that I got all the tests [referring to medical tests and referrals]...But also, they gave me a community through their Facebook.". [Participant L, Female, Age Range 48-57]*
Post-COVID Service Experiences	2. *[About the post-COVID service] "It's reassurance as well. And then if they find something they obviously can tell you straight away. You don't need to go and look for for something, and then it's just even acknowledgement of of somebody understand you. And and know what what is going in your body and you are not on your own…so that's very important.". [Participant F, Female, Age Range 48-57]*
Post-COVID Service Experiences	3. *"Yeah, I feel like I was a bit disappointed with my like long COVID clinic. It was just like a 60 minute phone call.". [Participant S, Female, Age Range 28-37]*
Post-COVID Service Experiences (Healthcare Professional (HCP) Perspective)	4. *"I think everybody's learning from each other, but no, not not haven't really kind of accessed like a formal training day. Like I've dipped into webinars and bits of things that I've seen that people are colleagues. I know that are doing that I'm kind of interested in.". [Physiotherapist]*
Post-COVID Service Experiences (HCP Perspective)	5. *"The psychology offer could maybe be a bit stronger. I mean, it's quite good that we even consider it and we have psychologists in, but we just haven't been able to fill the role and then issues with space and*

	staffing. Again, it's just something that's fallen to the wayside.". [Doctor (Gastro)].
Post-COVID Service Experiences (HCP Perspective)	6. *"They didn't have the capacity to take on any of this work. So we we wanted to have very much a multispecialty approach to managing long COVID. But our respiratory physicians and cardiologists didn't have the time available to to join the team. So the challenges have been that from a medical perspective, it's very much fallen solely on me.". [Rehabilitation Medicine Consultant].*
Post-COVID Service Experiences (HCP Perspective)	7. *"…working with people with long COVID can feel quite daunting unless you've seen a lot of it. And the confidence that comes with kind of managing that clinical uncertainty of what's in front of you only comes through with experience.". [Physiotherapist].*
Post-COVID Service Experiences (HCP Perspective)	8. *"…a key barrier…is how we work across the integrated care system. So we're all on different electronic records and the information governance agreements extend to some things and not others. We don't yet have sight of how our patients are doing. You know, we assess them in clinic and we'll send them to rehab, but we don't really get hearing about their outcomes and that that is that is problematic both in terms of how we assess whether what we're doing is effective and and how we coordinate care, but it also limits us in our delivery.". [Physiotherapist].*
Post-COVID Service Experiences (HCP Perspective)	9.*"Diagnostics wise, I think at the beginning we did a lot of tests because we really didn't know what was going on. And as we've grown our clinical confidence and expertise…the number of tests reduced…". [Post COVID Lead].*

The semi-structured interviews with healthcare providers shed light on the experiences they faced while operating a Long COVID Service. Across all sites, the healthcare professionals confronted a multitude of obstacles, with a significant portion stemming from the pervasive clinical uncertainty surrounding Long COVID and the absence of standardised training programs. As a result, many healthcare providers had to rely on self-directed learning to stay updated and effective in their roles (quote 4, box 3). Furthermore, the persistent staffing and capacity issues within these specialised services meant healthcare teams often could not deliver the best possible care for people dealing with the long-lasting effects of COVID-19 (quotes 5 – 6, box 3). The semi-structured interviews additionally highlighted the clinical uncertainty surrounding the best management practices for Long COVID (quote 7, box 3), indicating a pressing need for further research and guidance (e.g., by having models of care commissioned to achieve this) in this emerging field. Healthcare professionals also underscored the difficulties of navigating integrated care systems (quote 8, box 3), emphasising the need for improved collaboration and coordination among various healthcare entities to better address the multifaceted needs of people with Long COVID.

Despite these challenges, for many post-COVID services, while initially there were substantial clinical unknowns, there was now a sense of greater clinical expertise and confidence, resulting in more refined approaches in Long COVID care (quote 9, box 3).

Discussion

For peer review only

In our study, which examined perspectives from people with lived experience of Long COVID and healthcare professionals, we found three overarching themes: (1) Understanding Long COVID: Symptoms and Lived Realities, (2) Long COVID Care: Non-Specialist Service Experiences and (3) Long COVID Care: Post-COVID Service Experiences.

Our first theme resonates with contemporary research that underscores the intricate, individual and varied nature of the Long COVID journey. Our findings from people with Long COVID align with studies that have emphasised the diverse range of symptoms of this condition,(32) highlighting the importance of personalised care models that account for the multifaceted dimensions of Long COVID. The emphasis from people living with Long COVID on mental health aligns with other research,(33) which showed the psychological and sometimes traumatic toll of living with Long COVID, further stressing the need for integrated mental health support as part of comprehensive Long COVID management strategies.

Our study additionally highlighted that the impact of Long COVID on people can extend well beyond its physiological manifestations, significantly affecting their immediate surroundings and (if applicable), their working environments. This study's findings corroborate with existing literature,(34,35) underlining the interplay between people living with Long COVID and the broader contexts in which they reside and work. The disruptions observed in social circles and routines align with the observations made in other studies,(36,37) highlighting the persistent challenges people living with Long COVID face as they navigate the unpredictable nature of Long COVID symptoms. Our study additionally echoes the experiences of those with cognitive impairments and reduced energy levels from Long COVID, resonating with other findings(38,39) that have documented the diverse impacts of Long COVID on professional functioning and adjustments required in work environments.(38,39) Notably, our findings indicated that the uncertainty caused by the lack of an effective treatment along with debilitating symptoms and the potential disruption across various aspects of daily life, makes living with long COVID especially challenging.

The healthcare professional's perspectives of symptoms resonated with those of the patients, highlighting a shared understanding of the complex nature of Long COVID – and the multifaceted impact it can cause. Overall, these two perspectives underline the urgent need for supportive structures that acknowledge the multifaceted dimensions of Long COVID, both within the personal and professional spheres, ensuring comprehensive and responsive care.

Our second theme, which exclusively examined the perspectives of people with Long COVID, underscored their frustrations with the accessibility and responsiveness of their GP practice and described challenges in contacting them and/or receiving a response. Prolonged waiting times were frequently reported in our semi-structured interviews, exacerbating their difficulties in accessing care. These experiences align with literature(40,41) and highlight the need for improved access to healthcare services for Long COVID patients and adequate support structures for managing this complex condition within primary care settings.

The experiences shared by people living with Long COVID in our study highlight a disconcerting circumstance wherein some GPs and other healthcare professionals seemingly avoid engaging in thorough discussions about the complexities of Long COVID. The people living with Long COVID consistently express feelings of being hurried through appointments or having their concerns dismissed. These findings align with emerging observations in the literature,(42–44) reflecting the challenge faced by healthcare providers in addressing the

For peer review only

nuanced needs of people living with Long COVID within the constraints of limited appointment times. Such negative interactions can significantly impact mental health, impeding the overall well-being and recovery of those navigating the complexities of their condition.(45)

Similar issues in patient-provider interactions within the context of Long COVID care have been described in other studies globally, emphasising the pressures on healthcare systems exacerbated by the pandemic. (44,46,47) The existing backlog of waiting lists, compounded by the strains of the COVID-19 pandemic, have strained healthcare resources, possibly contributing to the reported challenges in addressing Long COVID comprehensively within primary care settings. However, it is important to consider whether an individual's expectations align with the practical capacities of clinicians. Nevertheless, overall, these dynamics emphasise the urgent need for tailored approaches to address Long COVID within healthcare systems and fostering improved communication between both parties to align expectations with achievable outcomes.

Our study found that establishment and use of Long COVID-specific online groups (both patient-led and provider-led) emerge as a noteworthy peer-support mechanism, facilitating connection and information-sharing among people living with Long COVID. These findings resonate with current literature, highlighting the value of online platforms in fostering a sense of community and understanding among people with Long COVID. The positive impact observed in our study aligns with the observations from other studies(48,49) where they have emphasised the therapeutic benefits of peer support networks in Long COVID management, acknowledging their role in reducing feelings of isolation and offering practical insights. The potential for these digital spaces to serve as outlets for shared experiences and coping strategies reflects a promising avenue for people living with Long COVID navigating the complexities of this condition, affirming the significance of such platforms within the broader landscape of patient-centred care. Nevertheless, while these online groups can be an avenue for peer support, given their potential for a source of conflicting information and misinformation, there remains a demand for accurate, up-to-date information to ensure individuals have reliable and current data which could support medical management.(50,51)

Lastly, a lack of clinical understanding of Long COVID among healthcare providers, as reported (or experienced) by people with Long COVID, aligns with previous findings.(52) This underscores the persistent challenge in understanding Long COVID, signifying the need for continued research and standardised approaches to improve patient care and outcomes in this complex condition.

The third theme aligns with discussions in the literature surrounding specialised post-COVID services. This theme was divided into two perspectives: that of people with Long COVID and healthcare professionals providing care within the post-COVID service.

In our interviews, we found that diverse models of post-COVID services were being used, where some operate primarily face-to-face (post-COVID services a and c) and others adopt hybrid approaches (post-COVID services b, d, e, and f), suggesting flexibility to tailor care to individual needs. The hybrid nature of post-COVID services potentially reflects a responsiveness to patient requirements, ranging from entirely virtual to incorporating in-person components such as home visits when necessary. Additionally, considerations such as pandemic-related infection control measures and staffing constraints within these post-COVID

For peer review only

service models may have influenced preference and practicality of a virtual service for certain sites and individuals.(53)

For people with Long COVID, the positive perception of expert care within these clinics echoes the sentiments expressed by recent research(54) stressing the importance of multidisciplinary care teams in addressing the complex and varied symptomatology of Long COVID. While immediate clinical improvement may not always be observed, many people living with Long COVID described the transformative nature of their interactions with healthcare professionals in these specialised clinics. Importantly, for many, it was a place where they were given time to discuss their symptoms and where they were validated and understood. This finding agrees with existing literature,(55,56) reinforcing the crucial role of empathy in health and social care professionals. We underscore its inherent importance in any service, with a particular emphasis on its heightened relevance in situations marked by clinical uncertainty. Further, our findings resonate with existing literature that reflects the pivotal role that post-COVID services play in addressing the complex needs of people living with Long COVID. Our study's insights also align with recommendations from previous studies(57,58) where an emphasis on the importance of multidisciplinary care teams and specialised expertise in tackling the intricate symptomatology of Long COVID was given.

For the virtual post-COVID services, drawbacks potentially include limited medical examination, challenges in effective communication and the risk of exacerbating health inequalities due to disparities in technology access.(59) The lack of hands-on assessments in virtual care may lead to delays in diagnosis or treatment and the digital nature of interactions may limit emotional connections. Additionally, the digital divide could exclude individuals with limited access to technology, hindering inclusivity in these support networks.(60) Addressing these concerns is crucial for ensuring a comprehensive and equitable approach to managing the complexities of Long COVID.

For healthcare professionals working within the post-COVID service, they reported clinical uncertainty, 'learning on the job' and a lack of resources – issues all previously noted in other studies.(52,61) Nevertheless, given the largely positive reviews of the post-COVID services, there was a sense that the healthcare professionals in the post-COVID service were able to align their clinical approach with the diverse expectations of those under their care. Striking a balance between patient expectations and the intricacies of their conditions has been well reported in the literature – an essential approach for reducing patient dissatisfaction and potentially improving health outcomes.(52,62) Although there remains clinical uncertainty with respect to Long COVID, efforts to address best practices towards assessing, treating and rehabilitating have been made with a training programme developed by NHSE, which launched in June 2023.(63)

We agree with suggestions that as the body of Long COVID evidence and diagnostic criteria evolves, there may be a need to revise referral criteria for specialist services to ensure the most efficient allocation of resources.(52,64) Similarly, self-referral to post-COVID clinics could be effective if these clinics could adequately manage the demand, which hinges on factors such as staffing and other available resources. However, the viability of such an approach is likely to vary since it heavily relies on the specific capabilities of each post-COVID service and may not be universally applicable.

Limitations

For peer review only

We acknowledge potential limitations in our methodology. Voluntary participation and its potential to introduce self-selection bias (where individuals who participated differ in relevant clinical characteristics from those who do not) is a limitation.(65) Additionally, the focus on English-speaking participants may exclude those who primarily communicate in other languages, potentially overlooking unique perspectives within linguistically diverse communities. As this study recruited people attending post-COVID services only, this excludes opinions and experiences of those who could not access these services, but were/are living with Long COVID. This limitation could result in a lack of representativeness and underrepresentation of certain subgroups. Nevertheless, our sample's demographics appear to be aligned with the utilisation of post-COVID services by age, gender, ethnicity in England, as recorded by NHSE.(66)

However, the underrepresentation of males is a potential limitation, which might limit the comprehensiveness of our findings and hinder a complete understanding of gender-specific experiences. The overrepresentation of White British/White ethnicities potentially limits the generalisability of our findings to more diverse populations, particularly as evidence suggests that ethnic minorities have been disproportionately impacted by COVID-19(67) and Long COVID has been reported across various ethnic groups.(68) The high representation of individuals with higher education levels and healthcare employment introduces a potential bias, as experiences and access to healthcare resources may differ for those with varied educational backgrounds and occupational statuses.(69) Further, the limited age range of participants may not fully capture the experiences of younger or older individuals with Long COVID.

Overall, although this population is not necessarily representative of the populations who have developed Long COVID at high rates, (1) these findings offer a particular lens through which to understand Long COVID, providing valuable insights into the experiences of this specific subgroup.

Conclusions

Our study reveals examples of both good care practices and challenges within healthcare provision for people with Long COVID. We underscore the urgent need for a patient-centred approach to Long COVID, addressing its diverse impacts on physical, mental health, social and environmental factors. Effective communication, specialised expertise and comprehensive support systems are pivotal – elements requiring integration into tailored interventions and policies that can holistically address the needs of people with Long COVID. Ensuring continued, sustainable access to specialised care for people with Long COVID is needed to address their multifaceted needs. The success of post-COVID services hinges on the ability to offer ongoing rehabilitation that extends beyond immediate recovery, acknowledging the long-term impacts and complexities associated with this condition.

Footnotes

Participant Consent for Publication

Not Applicable.

Ethics Approval

This study involves human participants and ethical approval (IRAS Project ID: 303958) was granted by the Health Research Authority (HRA) and South Central—Berkshire Research

For peer review only

Ethics Committee (REC Reference: 22/SC/0047). All participants gave informed written consent to participate in the study before taking part.

Funding

This research was funded by NIHR (COV-LT2-0043) as part of the STIMULATE-ICP study. The funders had no role in the design of the study; in the collection, analyses, or interpretation of data; in the writing of the manuscript; or in the decision to publish the results.

Acknowledgements

Our sincere thanks to all participants in this study for their time and the information they provided.

This study is part of the STIMULATE-ICP study. The STIMULATE-ICP Consortium: Amitava Banerjee, Elizabeth Murray, Hakim-Moulay Dehbi, Hugh Montgomery, Sarah Clegg, Henry Goodfellow, Mel Ramasawmy, Yi Mu, Sampath Weerakkody, Ileana Selejan, David Sunkersing, Ashkan Dashtban, Paula Lorgelly, Melissa Heightman, Toby Hillman, Emma Wall, Caroline Leigh-Watkins, Denise Forshaw, Gordon Prescott, Gail Allsopp, Mark Gabbay, Gregory Lip, Dan Cuthbertson, Dan Wootton, Nefyn Williams, Mike G Crooks, Angela Green, Christina van der Feltz-Cornelis, Jenny Sweetman, Han-I Wang, Natalie Smith, Kamlesh Khunti, Lauren O'Mahoney, Rachael Evans, William D Strain, Rachel Botell, Nisreen Alwan, Donna Clutterbuck, Marija Pantelic, Chris Robson, Mike Brady, Rajarshi Banerjee, Cat Kelly, Angela Barone, Johannes Alberts, Rob Suriano, Lyth Hishmeh, Emily Attree, Jasmine Hayer, Rita Mallinson Cookson, Rachel Hext, Andrew Williams, Rachel Williams, Mag Leahy, Antony Loveless, Clare Loveless, Kim Horstmanshof, Ewen Brennan, Amanpreet Sarna, Manuel Gomes, Andrew Clegg, Valerio Benedetto, Melissa Dalton, Fauzia Begum, Fidan Turk, Michele Pansini, Anu Chandra, Gemma Clunie, Dominic Crocombe, Shane McAuliffe, Michelle Quaye, Farhat Gilani, Yusuf Jaami, and Nikki Cleary. An up-to-date version of Consortium members can be found on https://www.stimulate-icp.org/team. STIMULATE-ICP can be contacted at: info@stimulate-icp.org.

Disclaimer

The views expressed in this publication are those of the author(s) and not necessarily those of the National Institute for Health and Social Care Research or the Department for Health and Social Care.

Competing Interests

AB has received research funding from NIHR, British Medical Association, Astra Zeneca, National Institute of Aging and European Union. AB is a Trustee of Long COVID SoS. NAA has contributed in an advisory capacity to the World Health Organisation and the European Union Commission's Expert Panel on effective ways of investing in health meetings in relation to post-COVID-19 condition. MH is National Specialty Advisor for Long Covid, NHS England.

All other authors report no competing interests.

Supplementary Materials

1. Supplementary Material 1 - Patient Interview Schedule

For peer review only

2. Supplementary Material 2 - HCP Interview Schedule

Word Count (Excluding Tables and References): 4934.

Author Contributions: AB, MH and the STIMULATE-ICP Consortium conceived and designed the study. DS and MR conducted semi-structured interviews with both people with Long COVID and healthcare professionals. DS conducted data analysis and drafted the paper. MR, NA, DC, YM, KH, AB and MH provided constructive feedback on drafts of the manuscript and approved the final version. DS is the guarantor.

References:

1. Davis HE, McCorkell L, Vogel JM, Topol EJ. Long COVID: major findings, mechanisms and recommendations. Nature Reviews Microbiology 2023 21:3 [Internet]. 2023 Jan 13 [cited 2023 Sep 19];21(3):133–46. Available from: https://www.nature.com/articles/s41579-022-00846-2

2. Parker AM, Brigham E, Connolly B, McPeake J, Agranovich A V., Kenes MT, et al. Addressing the post-acute sequelae of SARS-CoV-2 infection: a multidisciplinary model of care. Lancet Respir Med [Internet]. 2021 Nov 1 [cited 2023 Sep 19];9(11):1328–41. Available from: https://pubmed.ncbi.nlm.nih.gov/34678213/

3. Callard F, Perego E. How and why patients made Long Covid. Soc Sci Med. 2021 Jan 1;268:113426.

4. Koc HC, Xiao J, Liu W, Li Y, Chen G. Long COVID and its Management. Int J Biol Sci [Internet]. 2022 [cited 2023 Sep 19];18(12):4768–80. Available from: https://pubmed.ncbi.nlm.nih.gov/35874958/

5. Walker S, Goodfellow H, Pookarnjanamorakot P, Murray E, Bindman J, Blandford A, et al. Impact of fatigue as the primary determinant of functional limitations among patients with post-COVID-19 syndrome: a cross-sectional observational study. BMJ Open [Internet]. 2023 Jun 1 [cited 2023 Sep 19];13(6):e069217. Available from: https://bmjopen.bmj.com/content/13/6/e069217

6. Aghaei A, Zhang R, Taylor S, Tam CC, Yang CH, Li X, et al. Impact of COVID-19 symptoms on social aspects of life among female long haulers: A qualitative study. Res Sq. 2022 Feb 10;

7. Mullard JCR, Kawalek J, Parkin A, Rayner C, Mir G, Sivan M, et al. Towards evidence-based and inclusive models of peer support for long covid: A hermeneutic systematic review. Soc Sci Med. 2023 Mar 1;320:115669.

8. Mantovani A, Morrone MC, Patrono C, Santoro MG, Schiaffino S, Remuzzi G, et al. Long Covid: where we stand and challenges ahead. Cell Death & Differentiation 2022 29:10 [Internet]. 2022 Sep 7 [cited 2023 Sep 19];29(10):1891–900. Available from: https://www.nature.com/articles/s41418-022-01052-6

9. Garner P. Paul Garner: For 7 weeks I have been through a roller coaster of ill health, extreme emotions, and utter exhaustion - The BMJ [Internet]. 2020 [cited 2024 Feb 3]. Available from: https://blogs.bmj.com/bmj/2020/05/05/paul-garner-people-who-have-a-more-protracted-illness-need-help-to-understand-and-cope-with-the-constantly-shifting-bizarre-symptoms/

10. BBC. Coronavirus: "Long Covid" patients need treatment programme, doctors say - BBC News [Internet]. 2020 [cited 2024 Feb 4]. Available from: https://www.bbc.co.uk/news/health-54031587

11. NHS England. NHS England » NHS to offer 'long covid' sufferers help at specialist centres [Internet]. 2020 [cited 2024 Feb 4]. Available from: https://www.england.nhs.uk/2020/10/nhs-to-offer-long-covid-help/

For peer review only

12. NHS England. National commissioning guidance for post COVID services [Internet]. 2022 [cited 2023 Oct 9]. Available from: https://www.england.nhs.uk/publication/national-commissioning-guidance-for-post-covid-services/

13. NICE. COVID-19 rapid guideline: managing the long-term effects of COVID-19 [Internet]. 2020 [cited 2023 Oct 9]. Available from: https://www.nice.org.uk/guidance/ng188

14. NHS. Long-term effects of COVID-19 (long COVID) - NHS [Internet]. 2024 [cited 2024 Feb 22]. Available from: https://www.nhs.uk/conditions/covid-19/long-term-effects-of-covid-19-long-covid/

15. Scenario: Management | Management | Long-term effects of coronavirus (long COVID) | CKS | NICE [Internet]. [cited 2024 Feb 22]. Available from: https://cks.nice.org.uk/topics/long-term-effects-of-coronavirus-long-covid/management/management/

16. World Health Organization. Guideline Clinical management of COVID-19: living guideline. 2023 [cited 2024 Feb 22]; Available from: http://apps.who.int/bookorders.

17. Baz SA, Fang C, Carpentieri JD, Sheard L. 'I don't know what to do or where to go'. Experiences of accessing healthcare support from the perspectives of people living with Long Covid and healthcare professionals: A qualitative study in Bradford, UK. Health Expectations [Internet]. 2023 Feb 1 [cited 2024 Feb 23];26(1):542–54. Available from: https://onlinelibrary.wiley.com/doi/full/10.1111/hex.13687

18. MacPherson K, Cooper K, Harbour J, Mahal D, Miller C, Nairn M. Experiences of living with long COVID and of accessing healthcare services: a qualitative systematic review. BMJ Open [Internet]. 2022 Jan 1 [cited 2024 Feb 23];12(1):e050979. Available from: https://bmjopen.bmj.com/content/12/1/e050979

19. BMA. Addressing the health challenge of long COVID Executive summary. 2022;

20. BMA. COVID-19: Impact of the pandemic on healthcare delivery [Internet]. 2023 [cited 2024 Feb 23]. Available from: https://www.bma.org.uk/advice-and-support/covid-19/what-the-bma-is-doing/covid-19-impact-of-the-pandemic-on-healthcare-delivery

21. Limb M. Staff and patients at risk from underinvestment in health and social care, says England's regulator. BMJ [Internet]. 2022 Oct 21 [cited 2024 Feb 23];379. Available from: https://www.bmj.com/content/379/bmj.o2537

22. Waitzman E. Staff shortages in the NHS and social care sectors [Internet]. 2022 [cited 2024 Feb 23]. Available from: https://lordslibrary.parliament.uk/staff-shortages-in-the-nhs-and-social-care-sectors/

23. Baz S, Fang C, Carpentieri J, Sheard L. Understanding the lived experience of Long Covid: A rapid literature review. 2021 [cited 2024 Feb 23]; Available from: https://www.ucl.ac.uk/covid-19-longitudinal-health-wellbeing/convalescence-long-covid-

24. Ladds E, Rushforth A, Wieringa S, Taylor S, Rayner C, Husain L, et al. Persistent symptoms after Covid-19: qualitative study of 114 "long Covid" patients and draft quality principles for services. BMC Health Serv Res [Internet]. 2020 Dec 1 [cited 2024 Feb 23];20(1). Available from: /pmc/articles/PMC7750006/

25. McNabb KC, Bergman AJ, Smith-Wright R, Seltzer J, Slone SE, Tomiwa T, et al. "It was almost like it's set up for people to fail" A qualitative analysis of experiences and unmet supportive needs of people with Long COVID. BMC Public Health [Internet]. 2023 Dec 1 [cited 2024 Feb 23];23(1):1–12. Available from: https://bmcpublichealth.biomedcentral.com/articles/10.1186/s12889-023-17033-4

For peer review only

26. Forshaw D, Wall EC, Prescott G, Dehbi HM, Green A, Attree E, et al. STIMULATE-ICP: A pragmatic, multi-centre, cluster randomised trial of an integrated care pathway with a nested, Phase III, open label, adaptive platform randomised drug trial in individuals with Long COVID: A structured protocol. PLoS One [Internet]. 2023 Feb 1 [cited 2023 Sep 19];18(2). Available from: https://pubmed.ncbi.nlm.nih.gov/36791116/

27. Schreier M. Qualitative Content Analysis in Practice. SAGE Publications. 2012. 24–25 p.

28. ATLAS.ti. Scientific Software Development GmbH; 2023.

29. Lumivero. NVivo (Version 12). 2017.

30. Hassan L, Edge D, Senior J, Shaw J. Staff and patient perspectives on the purpose of psychotropic prescribing in prisons: care or control? Gen Hosp Psychiatry. 2013 Jul 1;35(4):433–8.

31. Insua-Summerhays B, Hart A, Plummer E, Priebe S, Barnicot K. Staff and patient perspectives on therapeutic engagement during one-to-one observation. J Psychiatr Ment Health Nurs [Internet]. 2018 Nov 1 [cited 2024 Feb 9];25(9–10):546–57. Available from: https://onlinelibrary.wiley.com/doi/full/10.1111/jpm.12497

32. Michelen M, Manoharan L, Elkheir N, Cheng V, Dagens A, Hastie C, et al. Characterising long COVID: a living systematic review. BMJ Glob Health [Internet]. 2021 Sep 1 [cited 2023 Sep 19];6(9):e005427. Available from: https://gh.bmj.com/content/6/9/e005427

33. Houben-Wilke S, Goërtz YMJ, Delbressine JM, Vaes AW, Meys R, Machado FVC, et al. The Impact of Long COVID-19 on Mental Health: Observational 6-Month Follow-Up Study. JMIR Ment Health 2022;9(2):e33704 https://mental.jmir.org/2022/2/e33704 [Internet]. 2022 Feb 24 [cited 2023 Sep 19];9(2):e33704. Available from: https://mental.jmir.org/2022/2/e33704

34. Ziauddeen N, Gurdasani D, O'Hara ME, Hastie C, Roderick P, Yao G, et al. Characteristics and impact of Long Covid: Findings from an online survey. PLoS One. 2022;17(3 March).

35. Suran M. Long COVID Linked With Unemployment in New Analysis. Vol. 329, JAMA. 2023.

36. Samper-Pardo M, Oliván-Blázquez B, Magallón-Botaya R, Méndez-López F, Bartolomé-Moreno C, León-Herrera S. The emotional well-being of Long COVID patients in relation to their symptoms, social support and stigmatization in social and health services: a qualitative study. BMC Psychiatry [Internet]. 2023 Dec 1 [cited 2023 Sep 19];23(1):1–13. Available from: https://bmcpsychiatry.biomedcentral.com/articles/10.1186/s12888-022-04497-8

37. Schmachtenberg T, Müller F, Kranz J, Dragaqina A, Wegener G, Königs G, et al. How do long COVID patients perceive their current life situation and occupational perspective? Results of a qualitative interview study in Germany. Front Public Health [Internet]. 2023 [cited 2023 Sep 19];11. Available from: /pmc/articles/PMC10034079/

38. De Luca R, Bonanno M, Calabrò RS. Psychological and Cognitive Effects of Long COVID: A Narrative Review Focusing on the Assessment and Rehabilitative Approach. J Clin Med [Internet]. 2022 Nov 1 [cited 2023 Sep 19];11(21). Available from: https://pubmed.ncbi.nlm.nih.gov/36362782/

39. Zahouani I, Desmeules F, Perreault K, Campeau-Lecours A, Best K, Beaulieu-Bonneau S, et al. Physical and cognitive impairments in people suffering from long COVID: protocol for a longitudinal population-based cohort study. BMJ Open [Internet]. 2023 [cited 2023 Sep 19];13:64054. Available from: http://bmjopen.bmj.com/

40. Goyal DK, Mansab F, Naasan AP, Iqbal A, Millar C, Franklin G, et al. Restricted access to the NHS during the COVID-19 pandemic: Is it time to move away from the rationed clinical response? The

For peer review only

Lancet Regional Health - Europe [Internet]. 2021 Sep 1 [cited 2024 Feb 14];8:100201. Available from: http://www.thelancet.com/article/S2666776221001782/fulltext

41. McIntyre D, Chow CK. Waiting Time as an Indicator for Health Services Under Strain: A Narrative Review. Inquiry (United States) [Internet]. 2020 Apr 30 [cited 2024 Feb 14];57. Available from: https://journals.sagepub.com/doi/10.1177/0046958020910305

42. Purewal S, Byers K, Jamieson K, Zolfaghari N. The Conversation. 2023. People with long COVID continue to experience medical gaslighting more than 3 years into the pandemic.

43. Anonymous. As a doctor with long covid, I feel abandoned by the NHS. Vol. 380, BMJ (Clinical research ed.). 2023.

44. Au L, Capotescu C, Eyal G, Finestone G. Long covid and medical gaslighting: Dismissal, delayed diagnosis, and deferred treatment. SSM Qualitative research in health. 2022 Dec;2:100167.

45. Eriksen AA, Fegran L, Fredwall TE, Larsen IB. Patients' negative experiences with health care settings brought to light by formal complaints: A qualitative metasynthesis. J Clin Nurs [Internet]. 2023 Sep 1 [cited 2024 Feb 15];32(17–18):5816–35. Available from: https://onlinelibrary.wiley.com/doi/full/10.1111/jocn.16704

46. Hussain FA. Facilitating care: a biopsychosocial perspective on long COVID. British Journal of General Practice [Internet]. 2022 Jan 1 [cited 2023 Sep 19];72(714):30–1. Available from: https://bjgp.org/content/72/714/30

47. Kingstone T, Taylor AK, O'Donnell CA, Atherton H, Blane DN, Chew-Graham CA. Finding the "right" GP: a qualitative study of the experiences of people with long-COVID. BJGP Open [Internet]. 2020 Dec 1 [cited 2023 Sep 19];4(5):1–12. Available from: https://bjgpopen.org/content/4/5/bjgpopen20X101143

48. Russell D, Spence NJ, Chase JAD, Schwartz T, Tumminello CM, Bouldin E. Support amid uncertainty: Long COVID illness experiences and the role of online communities. SSM - Qualitative Research in Health. 2022 Dec 1;2:100177.

49. Day HLS. Exploring Online Peer Support Groups for Adults Experiencing Long COVID in the United Kingdom: Qualitative Interview Study. J Med Internet Res [Internet]. 2022 May 1 [cited 2023 Sep 19];24(5). Available from: /pmc/articles/PMC9128729/

50. Brown K, Yahyouche A, Haroon S, Camaradou J, Turner G. Long COVID and self-management. The Lancet. 2022 Jan;399(10322):355.

51. Aiyegbusi OL, Hughes SE, Turner G, Rivera SC, McMullan C, Chandan JS, et al. Symptoms, complications and management of long COVID: a review. J R Soc Med. 2021 Sep 15;114(9):428–42.

52. Ladds E, Rushforth A, Wieringa S, Taylor S, Rayner C, Husain L, et al. Developing services for long COVID: lessons from a study of wounded healers. Clin Med (Lond) [Internet]. 2021 Jan 1 [cited 2023 Sep 19];21(1):59–65. Available from: https://pubmed.ncbi.nlm.nih.gov/33479069/

53. Chung-Lee L, Catallo C. A new approach to digital health? Virtual COVID-19 care: A scoping review. Digit Health [Internet]. 2023 Jan 1 [cited 2024 Mar 1];9. Available from: /pmc/articles/PMC9926398/

54. Parkin A, Davison J, Tarrant R, Ross D, Halpin S, Simms A, et al. A Multidisciplinary NHS COVID-19 Service to Manage Post-COVID-19 Syndrome in the Community. J Prim Care Community Health [Internet]. 2021 [cited 2023 Sep 19];12. Available from: https://pubmed.ncbi.nlm.nih.gov/33880955/

For peer review only

55. Jeffrey D. Empathy, sympathy and compassion in healthcare: Is there a problem? Is there a difference? Does it matter? J R Soc Med [Internet]. 2016 Dec 1 [cited 2024 Feb 15];109(12):446–52. Available from: https://pubmed.ncbi.nlm.nih.gov/27923897/

56. Moudatsou M, Stavropoulou A, Philalithis A, Koukouli S. The Role of Empathy in Health and Social Care Professionals. Healthcare [Internet]. 2020 [cited 2024 Feb 15];8(1). Available from: /pmc/articles/PMC7151200/

57. Greenhalgh T, Sivan M, Delaney B, Evans R, Milne R. Long covid—an update for primary care. BMJ [Internet]. 2022 Sep 22 [cited 2023 Sep 19];378. Available from: https://www.bmj.com/content/378/bmj-2022-072117

58. Nalbandian A, Sehgal K, Gupta A, Madhavan M V., McGroder C, Stevens JS, et al. Post-acute COVID-19 syndrome. Nature Medicine 2021 27:4 [Internet]. 2021 Mar 22 [cited 2023 Sep 19];27(4):601–15. Available from: https://www.nature.com/articles/s41591-021-01283-z

59. Yao R, Zhang W, Evans R, Cao G, Rui T, Shen L. Inequities in Health Care Services Caused by the Adoption of Digital Health Technologies: Scoping Review. J Med Internet Res [Internet]. 2022 Mar 1 [cited 2024 Mar 1];24(3). Available from: /pmc/articles/PMC8981004/

60. Hernandez K, Roberts T. Leaving No One Behind in a Digital World. K4D [Internet]. 2018 [cited 2024 Mar 1]; Available from: https://assets.publishing.service.gov.uk/media/5c178371ed915d0b8a31a404/Emerging_Issues_LNOBDW_final.pdf

61. O'Hare AM, Vig EK, Iwashyna TJ, Fox A, Taylor JS, Viglianti EM, et al. Complexity and Challenges of the Clinical Diagnosis and Management of Long COVID. JAMA Netw Open [Internet]. 2022 Nov 1 [cited 2024 Feb 15];5(11):e2240332–e2240332. Available from: https://jamanetwork.com/journals/jamanetworkopen/fullarticle/2798146

62. Rushforth A, Ladds E, Wieringa S, Taylor S, Husain L, Greenhalgh T. Long Covid – The illness narratives. Soc Sci Med. 2021 Oct 1;286:114326.

63. NHS England. Post COVID Services [Internet]. 2023 [cited 2023 Oct 10]. Available from: https://www.e-lfh.org.uk/programmes/post-covid-services/

64. Devereaux A, Yang H, Seda G, Sankar V, Maves RC, Karanjia N, et al. Optimizing Scarce Resource Allocation During COVID-19: Rapid Creation of a Regional Health-Care Coalition and Triage Teams in San Diego County, California. Disaster Med Public Health Prep [Internet]. 2022 Feb 10 [cited 2023 Sep 19];16(1):321–7. Available from: https://www.cambridge.org/core/journals/disaster-medicine-and-public-health-preparedness/article/optimizing-scarce-resource-allocation-during-covid19-rapid-creation-of-a-regional-healthcare-coalition-and-triage-teams-in-san-diego-county-california/2C5495005A8CF15F4971884A2B80E578

65. Tripepi G, Jager KJ, Dekker FW, Zoccali C. Selection Bias and Information Bias in Clinical Research. Nephron Clin Pract [Internet]. 2010 Jun 1 [cited 2024 Feb 10];115(2):c94–9. Available from: https://dx.doi.org/10.1159/000312871

66. NHS England. Statistics » COVID-19 Post-Covid Assessment Service [Internet]. 2024 [cited 2024 Feb 17]. Available from: https://www.england.nhs.uk/statistics/statistical-work-areas/covid-19-post-covid-assessment-service/

67. Kirby T. Evidence mounts on the disproportionate effect of COVID-19 on ethnic minorities. Lancet Respir Med [Internet]. 2020 Jun 1 [cited 2024 Feb 10];8(6):547–8. Available from: http://www.thelancet.com/article/S2213260020302289/fulltext

For peer review only

68. Khullar D, Zhang Y, Zang C, Xu Z, Wang F, Weiner MG, et al. Racial/Ethnic Disparities in Post-acute Sequelae of SARS-CoV-2 Infection in New York: an EHR-Based Cohort Study from the RECOVER Program. J Gen Intern Med [Internet]. 2023 Apr 1 [cited 2024 Feb 10];38(5):1127–36. Available from: https://link.springer.com/article/10.1007/s11606-022-07997-1

69. McMaughan DJ, Oloruntoba O, Smith ML. Socioeconomic Status and Access to Healthcare: Interrelated Drivers for Healthy Aging. Front Public Health [Internet]. 2020 Jun 18 [cited 2024 Feb 10];8:231. Available from: /pmc/articles/PMC7314918/

For peer review only

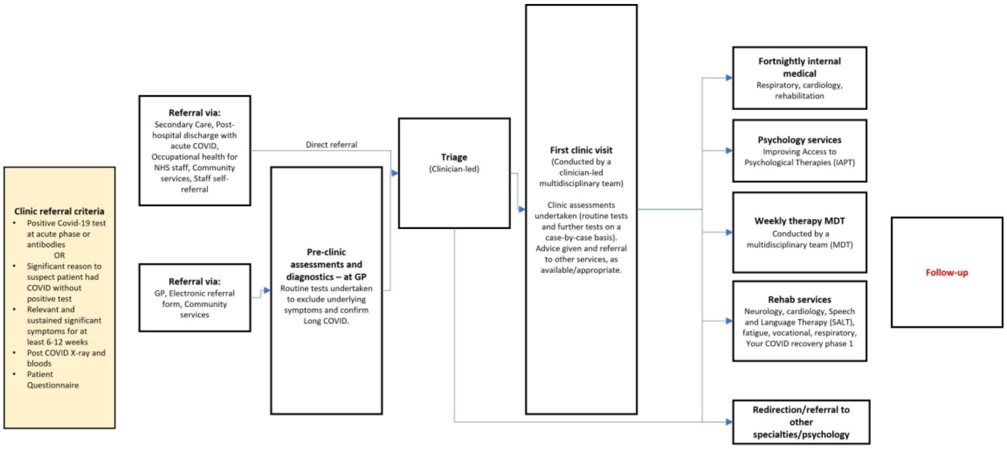

Figure 1. Long COVID Patient Pathway

298x132mm (330 x 330 DPI)

STIMULATE-ICP - WP1

Interview Schedule – Patients

Introduction

1. Review of Participant Information Sheet
2. Consent form
3. Timing and confidentiality
4. My name is
5. I am a researcher, I'm not a doctor or a psychologist and I don't have any clinical contact with your team.
6. I'm interested in finding out from you what your experiences have been with your care for Long-COVID
7. Overview of sections: your experiences, care you have received, your experiences of the service
8. There are no right answers
9. Appreciate symptoms include fatigue and brain fog, so let me know if you need to take a break etc.

**10. Precipitating events**	**Take me back, can you tell me about when you were first unwell with COVID-19 up to when you felt that you had Long Covid?**
Prompts	• Can you remember feeling unwell?<br>• Was there anything else you think may have contributed to how you were feeling? *What else going on at that time?*<br>• How do you think that experience relates to how you feel today?<br>• **Can you talk me through the support you received during this time?**<br>○ E.g. family, friends, GP?<br>○ How did you find out you had COVID-19? E.g. Who told you? When?<br>○ How did you feel when you found out it was COVID-19?<br>○ COVID-19 and other chronic conditions. Exacerbates other chronic conditions and affects management of it?<br>• When did you realise your symptoms weren't going away?<br>• **How did you find out about the post-COVID/Long Covid clinic**?
**11. Symptoms**	**Can you tell me about a time when you felt your Long-COVID was at its worst?**
Prompts	• How did it feel?<br>• How long did it last?<br>• Can you tell me how most days are for you at the moment?<br>• What's a good day for you?<br>• How long have you been feeling this way?<br>• Are there times when you don't feel this way? How long does this last?<br>• How has this impacted on your work and personal life

Patient Flow diagram

I would like to move on to think about the care you have received for your long-COVID. I'd like you to look at what some other people have experienced on their care journey at [name of clinic].

**Diagram discussion**	**When was it confirmed you had long covid? GP or after referral into clinic?**<br><br>**Has this been your experience of care?**

For peer review only - http://bmjopen.bmj.com/site/about/guidelines.xhtml

	**Do you recognise all the parts to this care diagram?**<br>**Are any parts of your experience missing?**<br>**What part of this care pathway do you think has been the most important to your experience?**

**12. Management of Long COVID**	**What treatment are you receiving for your long-COVID at the moment?**
Prompts	• Can you tell me about the care you have received/are receiving for your Long-COVID from doctors?<br>*e.g. who (GP, clinic nurse/doctor/ physiotherapist/ occupational therapist)*<br>*e.g. where (GP clinic, hospital clinic, community/third sector/private doctor)*<br>*e.g. when (routine/booked appointments, length of appointments)*<br>*e.g. what (medications/treatment/lifestyle advice/social prescriptions/physiotherapy/ vocational rehab/ psychology)*<br>• How did you find the treatment?<br>• How helpful has this treatment been? E.g. what was most helpful and least helpful?<br>• Diagnosis/attending appointments when you're not feeling well? *Better to go and show what you're like at your worst or not be able to make it at all.*
**13. Service evaluation**	• **Can you talk me through anything you would change about the long-COVID care you have received?**
Prompts	• How long did you have to wait to be seen by your GP/ by the PCAS service/ by onward rehabilitation services?<br>• Have any parts of your care been particularly helpful or unhelpful?<br>• Have you received the care you expected to when you were referred?<br>• Did you feel listened to and understood by your GP/ clinic doctors/ any therapist you have met<br>• What would you like to happen in the future?<br>• Were there any specific types of doctor you would have liked to have seen?<br>• Would you have like to have seen a psychologist<br>• Did you receive support form a physiotherapist or occupational therapist?<br>• Would you like to go back to the clinic in the future?<br>• Would you recommend the PCAS clinic / rehabilitation service to other patients<br>• **Where do you go to for information about long covid?**<br>• Do you feel like HCPs are knowledgeable / open to new evidence about LC?

Wrapping up

14. Is there anything else you would like to tell me?
15. How have you found this interview?
16. Do you have any questions?

For peer review only - http://bmjopen.bmj.com/site/about/guidelines.xhtml

17. Thank you for your time.
18. Give sources of further support handout.
19. Give baseline questionnaire.

STOP RECORDING

Baseline questions to complete at end of interview.

If they have already answered this during the interview, can move on to the next question, or confirm.

Script:

Thank you for participating in this interview. We need to collect some background information about you, so that we can describe which groups of people participated in our study. This will be stored separately from the transcript of this conversation and will not include identifying information, such as your name or date of birth. If there is any question you would prefer not to answer we can move on.

- What is your age?
- What is your gender?
- How would you describe your ethnic group?
- What languages do you speak?
- What is your highest level of educational qualification?
- What is the nature of your employment? (If you're no longer employed due to LC symptoms, what was your previous job)
- Aside from your Long Covid symptoms, do you have any longstanding illnesses, diseases or medical conditions? These can include conditions relating to your physical and mental health (e.g. asthma, depression, diabetes, previous heart attack, osteoporosis).

Information for interviewers

Do not recommend that patients should complain. However if they ask where to complain or raise concerns, you can direct them to their local PALS. Information for that is in the participant information sheet, or can offer to resend the details.

If patient is very distressed, you can recommend that they get in touch with their GP or post-covid service. Similarly, you can remind them that they can let the GP know if anything has changed. You can let them know that the post-covid service has offered that we can help them connect with them, if they would like to see them.

If you have an immediate concern that there is a risk of harm to the patient or to others, e.g. they express suicidal thoughts without prompting, you have a duty to report this. In this instance, you should phone the site clinical lead. You can inform the patient that it is your responsibility to report this, and go ahead without their consent. However you should only do this if you have a compelling reason to do so; otherwise do not breach confidentiality, suggest that the patient follows up with the GP or post-covid service.

For peer review only - http://bmjopen.bmj.com/site/about/guidelines.xhtml

Interview guide – Interview with HCP

n=24, up to four health professional categories will be nominated at each site, and recruitment will take place within those professionals. Max four interviews per site across the six sites.

The questions below are a guide but will be adapted based on HCP type and service delivery model.

**1. Introduction**	• Introductions<br>• Purpose of discussion is to understand experience of HCPs within each site to enable us to understand how usual care is being delivered.
**2. Individual background**	**Can you tell me about your clinical background?**
Prompts	• Background, speciality, grade, years since qualification
**3. Employment in current service**	**How did you come to work in the Post COVID service?**
Prompts	• E.g. applied, seconded (where did you come from?)<br>• How long is your contract or secondment?<br>• Do you work from home or on site?<br>• Did you feel that you had previous skills or experience that was relevant to this setting?<br>• Do you want to continue working in this field in the long term?<br>• Have you experienced any long COVID symptoms yourself?
**4. Training**	**Can you tell us about the induction, training and support you have received in this role?**
	• Were you offered any training when you entered the role? [*relative to what you would expect moving into another new department, specific to LC*]<br>• Are there resources available, and have you used them?<br>• For example, have you accessed the NHS Futures site for Long COVID, attended webinars?<br>• Do you feel able to keep up to date with developments?<br>• What additional training in Long-COVID care would you like? (How would you like it to be delivered)
**5. HCP experience**	**What are the main opportunities or challenges of your role?**
Prompts	• Do you feel you have adequate time in your job plan to do this work?<br>• Managing patient disappointments (waiting list, no clear treatment)

For peer review only - http://bmjopen.bmj.com/site/about/guidelines.xhtml

Workforce experience –Interview Guide

	• Keeping up with patient research/knowledge / responding to it / non-validated interventions and home remedies / patient safety *"This is a rapidly moving area, with patients doing a lot of their own research. What are your concerns and/or strategies for handling this?"*
**6. Patient presentation**	**What is the typical presentation of patients for the services you deliver?**
Prompts	• What symptoms do patients present with most frequently?<br>• What underlying comorbidities are typically seen?<br>• How has this changed over the course of the pandemic? (e.g. different waves, variants, vaccination, etc.)?<br>• *[pick up differences from service lead answers]*
**7. Patient experience of care**	**How would you describe a typical patient pathway in this service?**
Prompts	• **Introduce map.**<br>• What is the typical experience of referral, experience in clinic, and onward referral.<br>• What are factors that lead to a different experience?<br>• What would you describe as a good outcome or a poor outcome for a patient on the pathway? [audit, challenge]<br>• How would you describe patient access to rehab or psychology support?<br>• NHS Staff?<br>• Community vs previously hospitalised Long covid patients? Has this changed?
**8. Quality of care**	**Where do you feel the service delivers good care for patients?**
Prompts	• Have you had feedback from patients on what works well, or what they would like to improve?<br>• What do you feel are the barriers to delivering good care (if any)? E.g. high vacancy rate, high demand/low capacity?<br>• Can you identify any barriers to access to care?<br>• What are the opportunities to improve patient experience of care?<br>• How do you think patient outcomes might be improved?<br><br>*What do we mean? Quality vs effectiveness.*
**9. Inequalities**	**In your experience, has there been equitable uptake of the service across different groups in society e.g. those from socially disadvantaged backgrounds?**
Prompts	- Who is referred vs who turns up? How do you manage DNAs?<br>- Who is accessing the clinic?

For peer review only - http://bmjopen.bmj.com/site/about/guidelines.xhtml

Workforce experience –Interview Guide

	- Why do you think this may be?<br>- Do you think those most at need are accessing the service?<br>- Are there particular socio-demographic groups who struggle to access the clinic?<br>- What do you think may be causing the equal/unequal uptake?<br>- Is the clinic doing anything to try and actively access those most in need?
**10.**	**What kind of immediate improvements do you want to see in the service?**
	- Funding<br>- Staffing<br>- Evidence for change might lead to overloaded capacity
11. **Wrapping up**	**Opportunity to ask questions or add in further detail**

Stop recording

<u>Baseline questions to complete at end of interview.</u>

If they have already answered this during the interview, can move on to the next question, or just confirm details.

Script:

Thank you for participating in this interview. We need to collect some background information about you, so that we can describe which groups of people participated in our study. This will be stored separately from the transcript of this conversation and will not include identifying information, such as your name or date of birth. If there is any question you would prefer not to answer we can move on.

- What is your age?
- What is your gender?
- How would you describe your ethnic group?
- Can you provide the following details about your role:
  - Background -
  - Speciality –
  - Grade –
  - Years since qualification
- Have you had Long Covid yourself?

For peer review only - http://bmjopen.bmj.com/site/about/guidelines.xhtml
For peer review only

Standards for Reporting Qualitative Research (SRQR)*

http://www.equator-network.org/reporting-guidelines/srqr/

Title and abstract

	Page/line no(s).
**Title** - Concise description of the nature and topic of the study Identifying the study as qualitative or indicating the approach (e.g., ethnography, grounded theory) or data collection methods (e.g., interview, focus group) is recommended	1
**Abstract** - Summary of key elements of the study using the abstract format of the intended publication; typically includes background, purpose, methods, results, and conclusions	1

Introduction

**Problem formulation** - Description and significance of the problem/phenomenon studied; review of relevant theory and empirical work; problem statement	2
**Purpose or research question** - Purpose of the study and specific objectives or questions	2

Methods

**Qualitative approach and research paradigm** - Qualitative approach (e.g., ethnography, grounded theory, case study, phenomenology, narrative research) and guiding theory if appropriate; identifying the research paradigm (e.g., postpositivist, constructivist/ interpretivist) is also recommended; rationale**	2-3
**Researcher characteristics and reflexivity** - Researchers' characteristics that may influence the research, including personal attributes, qualifications/experience, relationship with participants, assumptions, and/or presuppositions; potential or actual interaction between researchers' characteristics and the research questions, approach, methods, results, and/or transferability	3
**Context** - Setting/site and salient contextual factors; rationale**	2-3
**Sampling strategy** - How and why research participants, documents, or events were selected; criteria for deciding when no further sampling was necessary (e.g., sampling saturation); rationale**	2-3
**Ethical issues pertaining to human subjects** - Documentation of approval by an appropriate ethics review board and participant consent, or explanation for lack thereof; other confidentiality and data security issues	19/20 (Ethics Approval)
**Data collection methods** - Types of data collected; details of data collection procedures including (as appropriate) start and stop dates of data collection and analysis, iterative process, triangulation of sources/methods, and modification of procedures in response to evolving study findings; rationale**	3 (Methods)

For peer review only

For peer review only - http://bmjopen.bmj.com/site/about/guidelines.xhtml

**Data collection instruments and technologies** - Description of instruments (e.g., interview guides, questionnaires) and devices (e.g., audio recorders) used for data collection; if/how the instrument(s) changed over the course of the study	4 (Data Analysis)
**Units of study** - Number and relevant characteristics of participants, documents, or events included in the study; level of participation (could be reported in results)	3 (Methods); 7(Table 1); 9 (Table 2)
**Data processing** - Methods for processing data prior to and during analysis, including transcription, data entry, data management and security, verification of data integrity, data coding, and anonymization/de-identification of excerpts	3-5 (Methods, Data Analysis)
**Data analysis** - Process by which inferences, themes, etc., were identified and developed, including the researchers involved in data analysis; usually references a specific paradigm or approach; rationale**	4 (Data Analysis)
**Techniques to enhance trustworthiness** - Techniques to enhance trustworthiness and credibility of data analysis (e.g., member checking, audit trail, triangulation); rationale**	4 (Data Analysis)

Results/findings

**Synthesis and interpretation** - Main findings (e.g., interpretations, inferences, and themes); might include development of a theory or model, or integration with prior research or theory	5-15
**Links to empirical data** - Evidence (e.g., quotes, field notes, text excerpts, photographs) to substantiate analytic findings	5-15

Discussion

**Integration with prior work, implications, transferability, and contribution(s) to the field -** Short summary of main findings; explanation of how findings and conclusions connect to, support, elaborate on, or challenge conclusions of earlier scholarship; discussion of scope of application/generalizability; identification of unique contribution(s) to scholarship in a discipline or field	15-19
**Limitations** - Trustworthiness and limitations of findings	19

Other

**Conflicts of interest** - Potential sources of influence or perceived influence on study conduct and conclusions; how these were managed	20
**Funding** - Sources of funding and other support; role of funders in data collection, interpretation, and reporting	20

*The authors created the SRQR by searching the literature to identify guidelines, reporting standards, and critical appraisal criteria for qualitative research; reviewing the reference lists of retrieved sources; and contacting experts to gain feedback. The SRQR aims to improve the transparency of all aspects of qualitative research by providing clear standards for reporting qualitative research.

For peer review only - http://bmjopen.bmj.com/site/about/guidelines.xhtml

**The rationale should briefly discuss the justification for choosing that theory, approach, method, or technique rather than other options available, the assumptions and limitations implicit in those choices, and how those choices influence study conclusions and transferability. As appropriate, the rationale for several items might be discussed together.

Reference:

O'Brien BC, Harris IB, Beckman TJ, Reed DA, Cook DA. **Standards for reporting qualitative research: a synthesis of recommendations.** *Academic Medicine*, Vol. 89, No. 9 / Sept 2014 DOI: 10.1097/ACM.0000000000000388

For peer review only

For peer review only - http://bmjopen.bmj.com/site/about/guidelines.xhtml
